# Postsynthetic modification of N-heterocyclic diazoolefins *via* backbone metallation

**DOI:** 10.1039/d6dt00943c

**Published:** 2026-07-02

**Authors:** Bastiaan Kooij, Sebastian Nicola Lange, Farzaneh Fadaei-Tirani, Rosario Scopelliti, Kay Severin

**Affiliations:** a Institut des Sciences et Ingénierie Chimiques, École Polytechnique Fédérale de Lausanne (EPFL) 1015 Lausanne Switzerland kay.severin@epfl.ch

## Abstract

Diazoolefins can be functionalized by postsynthetic modification *via* backbone metallation. Reactions with electrophiles allow the regioselective introduction of silyl, phosphino, stannyl, borate, and alcoholate groups. The methodology was also used for the synthesis of a silyl-bridged bis-diazoolefin.

## Abstract 2

Diazoolefins can be functionalized by postsynthetic modification *via* backbone metallation.

## Graphical abstract

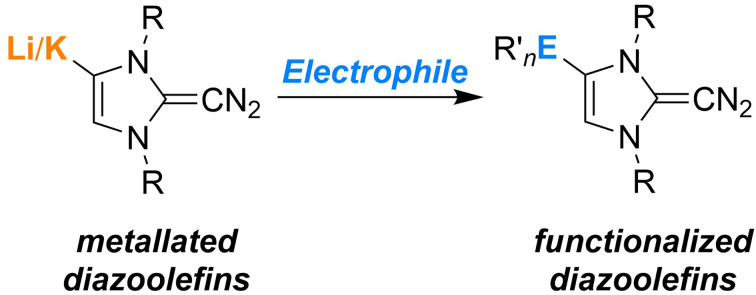

## Introduction

**Fig. 1:**
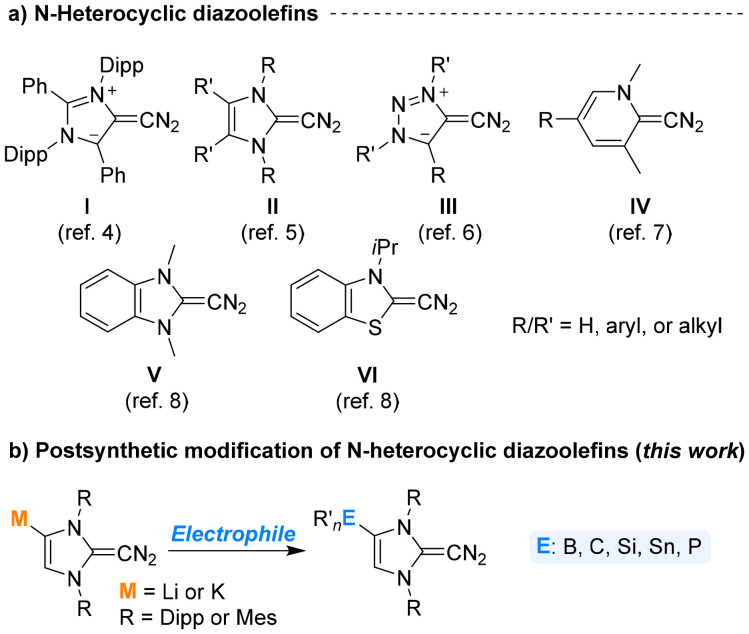
(a) Structures of N-heterocyclic diazoolefins reported so far (Dipp = 2,6-diisopropylphenyl), and (b) synthesis of diazoolefins by postsynthetic modification.

Diazoolefins had the reputation of being highly reactive compounds that rapidly lose dinitrogen, even at low temperatures.^1–3^ The situation changed in 2021, when Hansmann and co-workers reported the synthesis of the N-heterocyclic diazoolefin I (Fig. 1a).^4^ A unique feature of this mesoionic diazo compound is its good thermal stability, allowing for characterization and reactivity studies at room temperature. Soon after, we described structurally related diazoolefins of type II, which are likewise stable at room temperature and above.^5*a*^ Recent additions to the family of N-heterocyclic diazoolefins are a tetramethyl-substituted compound of type II,^5*b*^ triazole- and pyridine-based compounds of type III ^6^ and IV,^7^ as well as the benzannulated diazoolefins V and VI.^8^ The unusual thermal stability of the diazoolefins I–VI can be linked to electronic delocalization.^9,10^

N-Heterocyclic diazoolefins display diverse reactivity.^10^ The dinitrogen group can be replaced by isocyanides or carbon monoxide to give vinylidene ketenimines^4,8^ or alkylidene ketenes,^6*a*,*c*,11^ respectively. Similar to diazoalkanes, diazoolefins can engage in (2 + 3) cycloaddition reactions.^5*a*,12^ Diazo compounds of type II were reported to undergo a methanol-induced dimerization to give strongly reducing quinoidal tetrazines.^13^

The carbon atom of the CN_2_ group in I–IV has ylidic character. As a result, N-heterocyclic diazoolefins can form adducts with main group Lewis acids or with transition metal complexes.^4,5*a*,6*a*,14–16^ Photochemically-induced loss of dinitrogen from N-heterocyclic diazoolefins gives highly reactive vinylidenes, which can undergo C–H activation reactions.^4,5*a*,6*a*,*b*^ Diazoolefin-derived vinylidenes are also of interest as ligands for metal complexes.^6*a*,17–21^

The reported routes for the synthesis of N-heterocyclic diazoolefins rely on N-heterocyclic olefins^4–7^ or carbodicarbenes^8^ as starting materials. The conversion into diazo compounds is achieved by using either nitrous oxide^22^ or organic azides^7^ as diazo transfer agents.

To further advance the chemistry of N-heterocyclic diazoolefins, it is desirable to have a larger pool of stable diazoolefins, including compounds with functional groups. Below, we describe a postsynthetic modification approach that allows adding different substituents to the backbone of diazoolefins of type II (Fig. 1b).

## Results and discussion

**Scheme 1:**
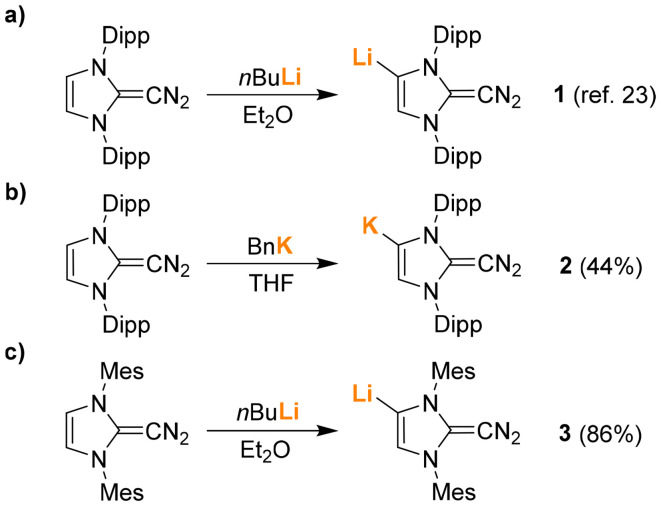
Synthesis of the metallated diazoolefins 1 (a), 2 (b), and 3 (c).

Recently, we have shown that a diazoolefin of type II with Dipp wingtip groups can be lithiated at C4 position with *n*BuLi (Scheme 1a).^23,24^ The resulting lithium salt, 1, was combined with BPh_3_ to give a diazoolefin with a negatively charged borate side group. We hypothesized that metallated diazoolefins such as 1 could be used to introduce a range of other substituents.

Organopotassium compounds can display a distinct reactivity when compared to organolithium compounds.^25^ Therefore, we have prepared salt 2 from the corresponding diazoolefin and benzyl potassium in THF (Scheme 1b). Moreover, we have made the lithium salt 3 using a diazoolefin with mesityl (Mes) instead of Dipp wingtip groups (Scheme 1c).

A single-crystal X-ray diffraction (XRD) analysis of salt 1 had revealed an intriguing 1D polymeric structure, with Li^+^ ions coordinated to the deprotonated heterocycle and to the exocyclic CN_2_ group.^23^ To examine if a coordination of the diazo group would also be observed for the analogous potassium salt 2, we performed an XRD analysis of 2.

**Fig. 2:**
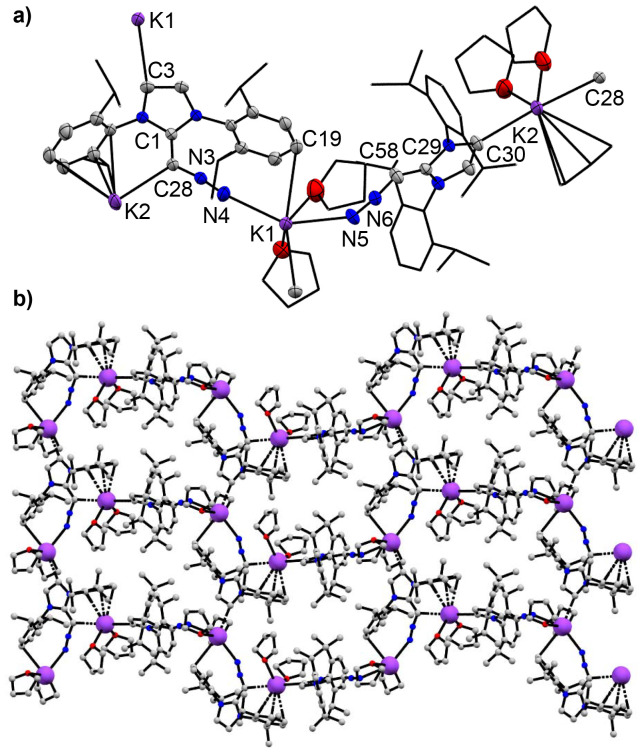
(a) Molecular structure of one repeat unit of the potassium salt 2 in the solid-state. Hydrogen atoms and uncoordinated solvent molecules are not shown for clarity. Thermal ellipsoids are drawn at the 50% probability level. (b) Ball and stick representation of the two-dimensional network structure of 2, as viewed along the crystallographic *a* axis.

Two distinct potassium cations are found in the solid-state structure of 2. One cation is bound to the terminal nitrogen atoms of two diazo groups (K1–N4 = 2.7798(14) Å, K1–N5 = 2.8259(14) Å), the deprotonated imidazole ring (K1–C3 = 2.8846(14) Å) and to two THF molecules (Fig. 2a). Moreover, there is a close contact to the *para* carbon atom of an adjacent Dipp group (K1⋯C4 = 3.400(2) Å). The second K^+^ is bound to the other deprotonated imidazole ring (K2–C30 = 2.835(2) Å), to the carbon atom of one exocyclic CN_2_ group (K2–C28 = 2.946(2) Å), and to two THF molecules, with additional cation–π contacts to the aromatic ring of an adjacent Dipp group.^26,27^ Since the diazoolefin anions act as polydentate bridging ligands, we observe overall a complex, two-dimensional network structure (Fig. 2b).

The fact that the CN_2_ groups in 2 have two distinct coordination modes is reflected in the solid-state IR spectrum, in which two *ν*(N_2_) bands are observed at 1972 and 1949 cm^−1^.

**Scheme 2:**
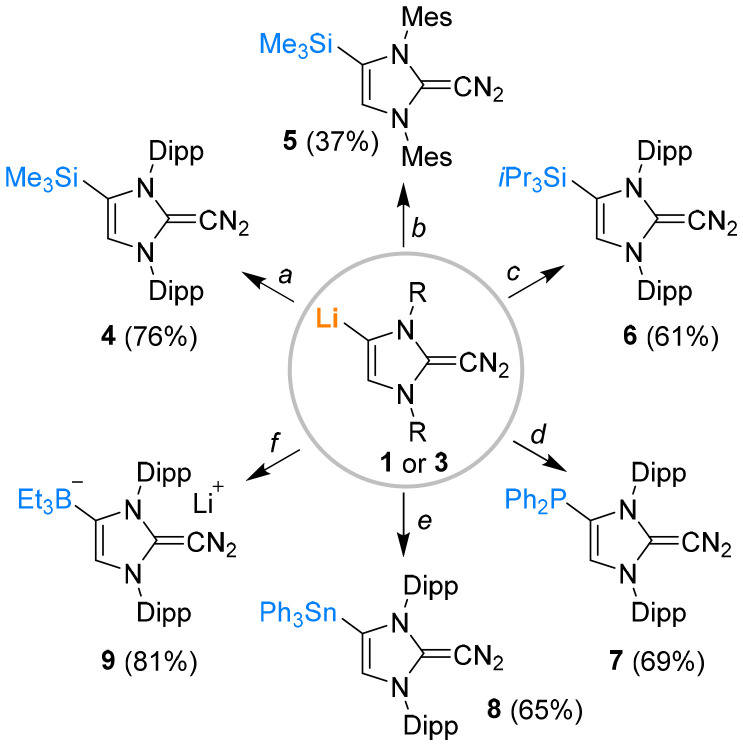
Synthesis of the backbone-functionalized diazoolefins 4–9. Conditions: (a) SiMe_3_Cl, Et_2_O. (b) SiMe_3_Cl, toluene. (c) *i*Pr_3_SiCl, THF. (d) PPh_2_Cl, Et_2_O. (e) SnPh_3_Cl, Et_2_O. (f) BEt_3_, Et_2_O.

With three different metallated diazoolefins in hand, we started to investigate backbone functionalization reactions. For first test reactions, we used trimethylsilyl chloride (TMSCl) as the electrophile. The regioselectivity of the reaction was considered to be a potential problem, because the ylidic carbon atom of the excocyclic CN_2_ group represents an alternative reaction center.^23^ However, when the lithiated diazoolefin 1 was combined with TMSCl in diethyl ether, the desired backbone-functionalized diazoolefin 4 was formed cleanly (Scheme 2). A similar silylation reaction could be accomplished using the diazoolefin 3 with mesityl instead of Dipp N-wingtip groups. The resulting coupling product 5 could be isolated in 37% yield. Changing the trimethylsilyl group for the more sterically demanding triisopropylsilyl gave diazoolefin 6 in 61% yield.

N-Heterocyclic diazoolefins are typically insoluble in highly apolar solvents. However, silylation significantly improved their solubility, as diazoolefins 4–6 could be readily extracted with pentane.

The use of chlorodiphenylphosphine as electrophilic coupling partner allowed access to the Ph_2_P-functionalized diazoolefin 7 in 69% yield. This compound is of potential interest as a bifunctional ligand for metal complexes.

Diazoolefin 8 with a triphenylstannyl group was prepared in 65% yield by reaction of 1 with Ph_3_SnCl in diethyl ether.

In analogy to the previously reported anionic diazoolefin with a triphenylborate substituent,^23^ we were able to prepare the anionic diazoolefin 9 in good yields by combining salt 1 with the alkyl borane BEt_3_ in diethyl ether.

**Fig. 3:**
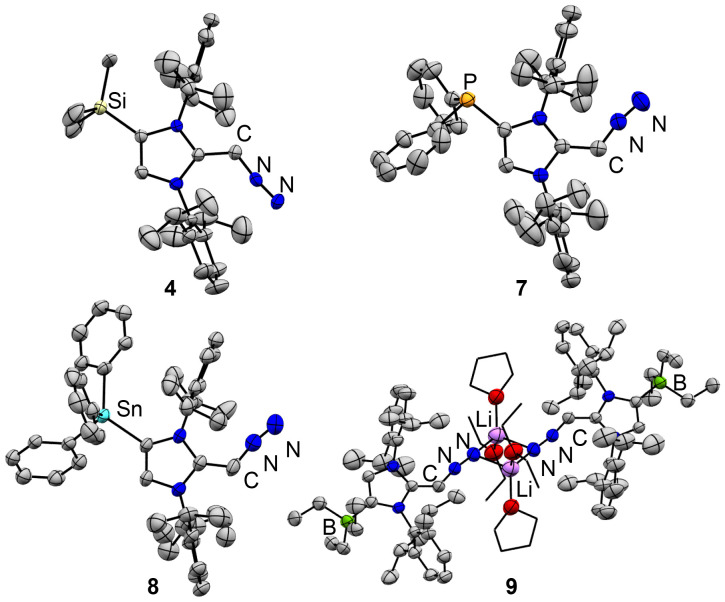
Molecular structures of diazoolefins 4 and 7–9 in the solid state. Hydrogen atoms and uncoordinated solvent molecules are not shown for clarity. Thermal ellipsoids are drawn at the 50% probability level.

The functionalized diazoolefins 4–9 were characterized by multinuclear NMR spectroscopy, mass spectrometry, and IR spectroscopy. In addition, the structures 4, 7, 8, and 9 in the solid-state were determined by single-crystal XRD (Fig. 3). The crystallographic analyses confirmed the attachment of the new functional groups to the imidazole backbone.

The metric parameters of the CN_2_ groups in the neutral diazoolefins 4, 7, and 8 are all similar, despite the presence of different substituents on the heterocycle (Table 1). For the anionic diazoolefin 9, on the other hand, the C–N_2_ bond was found to be markedly shorter (1.217(6) Å), while the N–N bond was found to be elongated (1.190(5) Å) (Table 1). The unique structural parameters of the CN_2_ group in 9 are reflected in the IR spectrum of 9. Whereas *ν*(N_2_) bands between 1972 and 1975 cm^−1^ were observed for 4, 7, and 8, a value of 2085 cm^−1^ was found for 9 (Table 1). The structural and spectroscopic differences can be linked to the fact that the CN_2_ group in 9 is coordinated *via* the terminal N-atoms to two lithium cations, forming a dimer in the solid state (Fig. 3). This solid-state structure contrasts the structure of the previously reported diazoolefin with a triphenylborate substituent, which exists as a monomer.^23^

**Table 1:** Key structural and spectroscopic data for the diazoolefins IPrCN_2_,^a^4, and 7–12

	C–CN_2_ (Å)	C–N_2_ (Å)	N–N (Å)	C–C–N (°)	*δ* (CN_2_) (ppm)	*ν* (N_2_) (cm^−1^)
IPrCN_2_ a	1.397(5)	1.285(5)	1.147(5)	121.3(4)	34.8	1984
4	1.393(2)	1.288(15)^a^	1.155(15)^a^	122.0(8)^a^	35.5	1972
7	1.377(19)	1.277(19)	1.149(18)	123.1(14)	36.7	1977
8	1.380(8)	1.287(8)	1.158(8)	122.4(6)	35.9	1975
9	1.395(6)	1.217(6)	1.190(5)	133.6(4)	22.1	2085
10 b	1.385(4)	1.277(4)	1.153(4)	123.2(3)	37.9	1971
11 b	1.389(3)	1.256(4)	1.158(4)	126.2(3)	33.2	1974
12	1.376(3)	1.289(2)	1.152(2)	123.3(17)	36.9	1974

a From ref. 5*a*. IPr = 1,3-bis(2,6-diisopropylphenyl)-imidazol-2-ylidene.

b Average values are given.

**Scheme 3:**
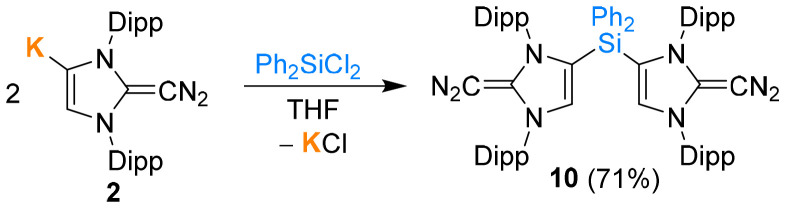
Synthesis of the Ph_2_Si-bridged diazoolefin 10.

Next, we investigated if we could link two diazoolefins *via* a silyl group. Initial attempts to couple the lithium salt 1 with R_2_SiCl_2_ (R = Me, Ph) resulted in a mixture of products. In contrast, we were able to obtain the desired bis-diazoolefin 10 by combining potassium salt 2 with Ph_2_SiCl_2_ in THF (Scheme 3).

**Fig. 4:**
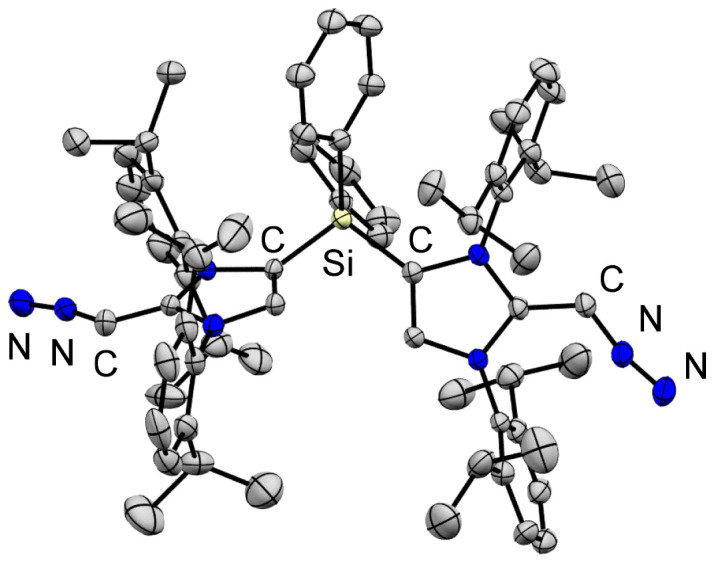
Molecular structure of 10 in the crystal. Hydrogen atoms and solvent molecules are not shown for clarity. Thermal ellipsoids are drawn at the 30% probability level.

The formation of 10 was corroborated by both mass spectrometry and infrared spectroscopy. The successful incorporation of two diazoolefin moieties was further supported by NMR spectroscopy, where integration of the proton signals indicated a 2 : 1 ratio of diazoolefin to SiPh_2_. The structure of 10 was finally established by single-crystal XRD (Fig. 4). The structural analysis revealed two independent bis-diazoolefins in the asymmetric unit with related structural parameters. Possible future applications of a dimer such as 10 include its use as a ligand or ligand precursor for dinuclear metal complexes.

**Scheme 4:**
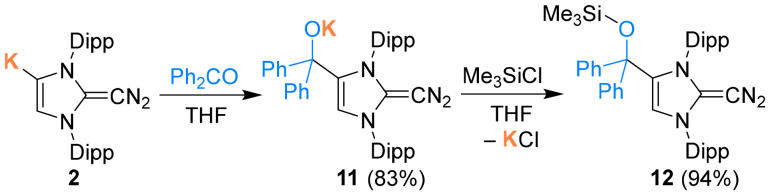
Synthesis of the diazoolefins 11 and 12.

Having established that the silyl, phosphino, stannyl and borate groups can be attached to the imidazole backbone, we next explored backbone functionalization by C–C bond formation. Combining the potassium salt 2 ^28^ with benzophenone in THF led to the formation of the alcoholate 11 in high yield (Scheme 4). The latter could be converted cleanly into the neutral diazoolefin 12 by silylation with TMSCl.

**Fig. 5:**
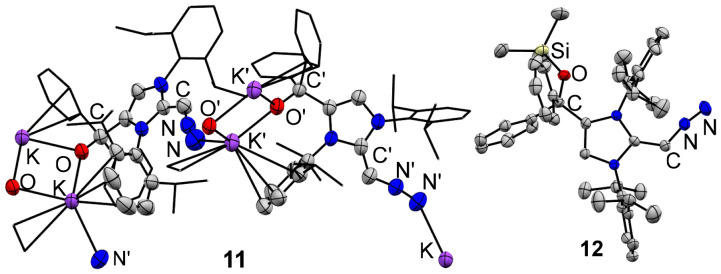
Molecular structure of one repeat unit of the potassium salt 11 in the solid-state and the molecular structure of 12 in the crystal. Hydrogen atoms and solvent molecules are not shown for clarity. Thermal ellipsoids are drawn at the 50% probability level.

The diazoolefins 11 and 12 were analyzed by NMR and IR spectroscopy, mass spectrometry, and single-crystal XRD (Fig. 4). In the solid state, the potassium cation in 11 is linked to the terminal nitrogen atom of the CN_2_ group and to two bridging alcoholate groups (Fig. 5). In addition, there are cation–π interactions with one Dipp group and with one phenyl group of the diphenylmethanolate substituent.

Interestingly, the bond lengths and angles of the CN_2_ groups in 11 and 12 are similar (Table 1), despite the fact that the K^+^ ion in 11 is coordinated to the terminal nitrogen atom of the CN_2_ group. Apparently, the cation-induced polarization of the diazoolefin group is not very pronounced.

## Conclusions

N-Heterocyclic diazoolefins are interesting compounds for applications in synthetic organic and inorganic chemistry. Thus far, one bottleneck for further developments has been the synthesis of these diazo compounds. The critical diazo transfer step requires careful adjustment of the reaction conditions. As a consequence, the structural diversity of this new compound class has been limited. Herein, we describe a method for the postsynthetic modification of imidazole-based diazoolefins. Metallation of the heterocycle was achieved by deprotonation with either *n*BuLi or BnK. The resulting salts could be coupled with a range of electrophiles, allowing the introduction of silyl, phosphino, stannyl, borate, and alcoholate groups. For the present work, we have focused mostly on reactions with a diazoolefin featuring Dipp wingtip groups. However, the successful synthesis of salt 3 and its conversion to the silylated diazoolefin 5 shows that other substituents are tolerated as well. Overall, we think that the methodology presented herein represents an appealing approach to diversifying diazoolefin chemistry.

## Author contributions

B. K. and K. S. initiated the study, B. K. and S. N. L. performed the experiments and analyzed the data, F. F.-T. and R. S. collected and processed the X-ray data, and B. K., S. N. L. and K. S. co-wrote the manuscript. All authors discussed the results and commented on the manuscript.

## Conflicts of interest

There are no conflicts to declare.

## Supplementary Material

DT-055-D6DT00943C-s001

DT-055-D6DT00943C-s002

## Data Availability

Details about the experimental data for this manuscript are provided in the supplementary information (SI). Supplementary information: containing synthetic procedures and experimental details. See DOI: https://doi.org/10.1039/d6dt00943c. CCDC 2497210, 2497211, 2530220, 2533230, 2533231, 2533232, 2533233, and 2533234 contain the supplementary crystallographic data for this paper.^29*a*–*h*^
