# Supplementary material for: Postsynthetic modification of N-heterocyclic diazoolefins via backbone metallation
Source: Dalton Trans. 2026 Jul 2;55(28):10632–6. doi: 10.1039/d6dt00943c (PMC13326623; doi:10.1039/d6dt00943c)
Supplement: DT-055-D6DT00943C-s001 [file DT-055-D6DT00943C-s001.pdf]

Supplementary Information (SI) for Dalton Transactions.
This journal is © The Royal Society of Chemistry 2026

Supplementary Information

Postsynthetic modification of N-heterocyclic diazoolefins *via* backbone metallation

Bastiaan Kooij,[a] Sebastian Nicola Lange,[a] Farzaneh Fadaei-Tirani,[a] Rosario Scopelliti,[a] and Kay Severin*[a]

[a]Institute of Chemical Sciences and Engineering, École Polytechnique Fédérale de Lausanne (EPFL), CH-1015 Lausanne, Switzerland

e-mail: kay.severin@epfl.ch

Contents

1. General 2
2. Syntheses.. 3
3. NMR Data... 14
4. ATR-IR Data... 43
5. XRD Analyses 49
6. References 68

1. General

Unless stated otherwise, reactions were performed under an atmosphere of dry dinitrogen using a glove box. All modified N-heterocyclic diazoolefins were stored in a dark glovebox freezer at –40 °C. Solvents were purchased dry and were stored under 4 Å molecular sieves for a minimum of two days prior to use. Diazoolefins and benzyl potassium were synthesised as described in literature.[1–3] If not stated otherwise, all other reagents were obtained from commercial sources and used without further purification.

The NMR spectra were measured on a Bruker Avance DPX-400 (^{1}H: 400 MHz), Bruker Avance NEO-500 (^{1}H: 500 MHz), Bruker Avance IIIHD-600 (^{1}H: 600 MHz), or Bruker Avance II (^{1}H: 800 MHz) spectrometer with BBFOz ATMA probe. Chemical shifts are given in parts per million (ppm) relative to their solvent signals [C_6D_6, 7.16 (^{1}H NMR), 128.06 (^{13}C NMR); d_8-THF, 1.72 and 3.58 (^{1}H NMR), 25.31 and 67.21 (^{13}C NMR)].

FT-IR spectra were acquired on a Perkin-Elmer Spectrum-One instrument with diamond-anvil configuration. Mass spectrometry measurements were performed on a LTQ Orbitrap FTMS instrument (LTQ Orbitrap Elite FTMS, Thermo Scientific) operated in the positive mode coupled with a robotic chip-based nano-ESI source (TriVersa Nanomate, Advion Biosciences). A standard data acquisition and instrument control system was utilised (Thermo Scientific) whereas the ion source was controlled by Chipsoft 8.3.1 software (Advion BioScience). Samples were loaded onto a 96-well plate within an injection volume of 5 µl. The experimental conditions for the ionisation voltage were +1.4 kV and the gas pressure was set at 0.30 psi. The temperature of ion transfer capillary was 200 °C. FTMS spectra were obtained in the 100-1000 *m*/*z* range in the reduce profile mode with a resolution set to 120,000. In all spectra, 1 microscan was acquired with a maximum injection time value of 1000 ms.

2. Syntheses

Note: *Although we have not encountered any problems, attention must be given to the fact that diazo compounds can liberate dinitrogen. Hence, we recommend that special safety precautions are taken when synthesising or working with diazoolefins.*

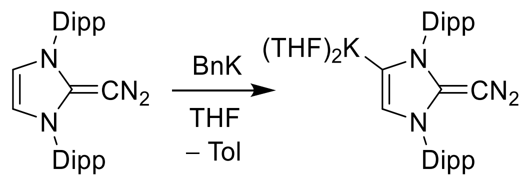

2 (44%)

Compound 2: $IDippCN_2$ (100 mg, 233 µmol, 1.00 equiv.) and benzyl potassium (30.4 mg, 233 µmol, 1.00 equiv.) were combined in a vial and cold THF (4 mL) was added at –40 °C while stirring. The mixture was stirred vigorously at –40 °C for 15 min, then warmed to RT, and stirred for 1 h. Diethyl ether (2 mL) was added to the resulting suspension to precipitate the product. The suspension was filtered and the solids were washed with diethyl ether (2 x 2 mL) and pentane (5 mL), affording compound **2** as a yellow solid after drying under reduced pressure (63 mg, 44%, *the calculated yield accounts for 2 THF molecules coordinated to K*).

Crystals, suitable for single-crystal XRD analysis, were grown by layering a concentrated THF solution of **2** with diethyl ether over the course of 5 days. The crystals appeared yellow in color.

^{1}H NMR (600 MHz, d_8-THF): δ 6.71 – 6.57 (m, 2H, CH_{arom}, Dipp), 6.57 – 6.33 (m, 4H, CH_{arom}, Dipp), 5.33 (s, 1H, $CH_{imidazole}$), 2.67 – 2.37 (double broad, 4H, $C\underline{H}(CH_3)_2$, Dipp), 0.69 (d, J = 6.9 Hz, 12H, CH_3, Dipp), 0.60 (d, J = 6.9 Hz, 12H, CH_3, Dipp). *Peaks were broadened, likely due to exchange reactions with the solvent molecules.*
^{13}C{^{1}H} NMR (101 MHz, d_8-THF): δ 151.7 ($\underline{C}=CN_2$), 147.5 (C_q, Dipp), 123.2 (CH_{arom}, Dipp), 28.8 ($C\underline{H}(CH_3)_2$, Dipp), 24.2 (CH_3, Dipp), 24.0 (CH_3, Dipp). Several peaks were missing due to broadening of the signals.
FT-IR (solid, ν, cm^{-1}): 1972 (N_2), 1949 (shoulder, N_2).
HRMS (nanochip-ESI/LTQ-Orbitrap) *m/z*: $[M + H]^+$ Calcd for $C_{28}H_{36}KN_4^+$ 467.2572; Found 467.2575. Calcd for $C_{28}H_{36}N_4^+$ ($IDippCN_2$) 429.3013; Found 429.3018. *The anionic $IDippCN_2$ fragment was not detected.*

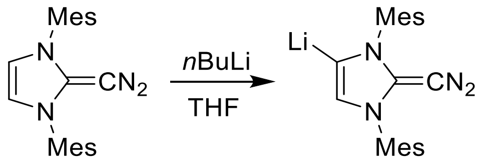

3 (86%)

Compound 3: IMesCN$_2$ (20.0 mg, 58.1 µmol, 1.00 equiv.) was suspended in diethyl ether (3 mL) and cooled to –40 °C. Then, *n*-butyllithium (2.5 M in hexanes, 29.8 µL, 74.4 µmol, 1.28 equiv.) was added at –40 °C. The mixture was stirred at RT for 1 h, after which it was filtered. The dark residue was washed with diethyl ether (2 × 1 mL), and the product was then extracted from the residue with THF (3 x 1 mL). The THF extracts were combined and the solvent was removed under reduced pressure. The remaining solids were washed with pentane (1 x 3 mL) and dried under reduced pressure to afford **3** as a dark-brown powder (17.5 mg, 86%).

^{1}H NMR (400 MHz, d_8-THF): δ 6.83 (s, 2H, CH$_{arom}$, Mes), 6.81 (s, 2H, CH$_{arom}$, Mes), 5.84 (s, 1H, CH$_{imidazole}$), 2.26 (s, 3H, CH$_3$, Mes, *para*), 2.25 (s, 3H, CH$_3$, Mes, *para*), 2.12 (s, 6H, CH$_3$, Mes, *ortho*), 2.11 (s, 6H, CH$_3$, Mes, *ortho*).
^{13}C{^{1}H} NMR (151 MHz, d_8-THF): δ 150.0 ($\underline{C}$=CN$_2$), 142.0 (C$_q$, Mes), 137.3 (C$_q$, imidazole), 137.3 (C$_q$, Mes), 137.1 (C$_q$, Mes), 136.7 (C$_q$, Mes), 136.3 (C$_q$, Mes), 129.0 (CH$_{arom}$, Mes), 128.7 (CH$_{arom}$, Mes), 122.8 (CH$_{imidazole}$), 25.0 (CN$_2$), 21.4 (CH$_3$, Mes), 21.3 (CH$_3$, Mes), 18.7 (CH$_3$, Mes), 18.5 (CH$_3$, Mes).
FT-IR (solid, ν, cm^{-1}): 1968 (N$_2$).
HRMS (ESI/QTOF) *m*/*z*: [M]$^+$ Calcd for $C_{22}H_{25}N_4^+$ (IMesCN$_2$) 345.2074; Found 345.2057,
[M]$^-$ Calcd for $C_{22}H_{23}N_4^-$ 343.1928; Found 343.1913.

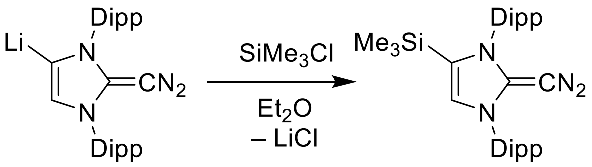

1 **4**

Compound 4: Diazoolefin **1** (8.8 mg, 20 µmol, 1.0 equiv.) was suspended in diethyl ether (0.5 mL) and $SiMe_3Cl$ (2.6 µL, 20 µmol, 1.0 equiv.) was added to the mixture. The suspension was stirred for 15 min and the solvent was removed under reduced pressure. The product was extracted with pentane (2 × 2 mL), and the pentane extracts were combined. The solvent was removed under reduced pressure, affording **4** as a light-yellow solid (7.7 mg, 76%).

Crystals, suitable for single-crystal XRD analysis, were grown by placing a pentane solution of **12** in a freezer at –40 °C. The crystals appeared light-yellow in color.

^{1}H NMR (600 MHz, C_6D_6) δ 7.26 (dt, *J* = 7.8, 7.8 Hz, 2H, CH_{arom}, Dipp), 7.13 (d, *J* = 7.7 Hz, 2H, CH_{arom}, Dipp), 7.10 (d, *J* = 7.8 Hz, 2H, CH_{arom}, Dipp), 6.27 (s, 1H, CH, imidazole), 3.03 (hept, *J* = 6.9 Hz, 2H, C<u>H</u>(CH_3)$_2$, Dipp), 2.97 (hept, *J* = 6.8 Hz, 2H, C<u>H</u>(CH_3)$_2$, Dipp), 1.55 (d, *J* = 6.9 Hz, 6H, CH_3, Dipp), 1.43 (d, *J* = 6.9 Hz, 6H, CH_3, Dipp), 1.23 (d, *J* = 6.8 Hz, 6H, CH_3, Dipp), 1.15 (d, *J* = 6.9 Hz, 6H, CH_3, Dipp), -0.16 (s, 9H, CH_3, $SiMe_3$).
^{13}C{^{1}H} NMR (151 MHz, C_6D_6) δ 154.2 (<u>C</u>=CN_2), 147.6 (C_q, Dipp), 147.2 (C_q, Dipp), 134.3 (C_q, Dipp), 133.3 (C_q, Dipp), 130.6 (CH_{arom}, Dipp), 130.5 (CH_{arom}, Dipp), 129.0 (C_q–Si), 127.1 (CH, imidazole), 124.2 (CH_{arom}, Dipp), 124.2 (CH_{arom}, Dipp), 35.5 (CN_2), 29.3 (C<u>H</u>(CH_3)$_2$, Dipp), 29.2 (C<u>H</u>(CH_3)$_2$, Dipp), 25.1 (CH_3, Dipp), 24.1 (CH_3, Dipp), 23.9 (CH_3, Dipp), 23.2 (CH_3, Dipp), –0.5 (CH_3, $SiMe_3$).
FT-IR (solid, *ν*, cm^{-1}): 1972 (N_2).
HRMS (nanochip-ESI/LTQ-Orbitrap) *m/z*: $[M + H]^+$ Calcd for $C_{31}H_{45}N_4Si^+$ 501.3408; Found 501.3410.

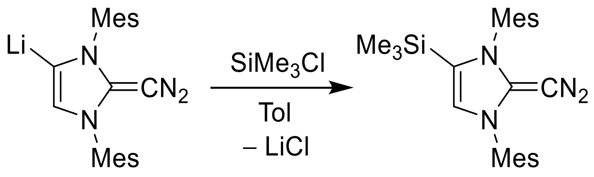

3 **5** (37%)

Compound 5: Diazoolefin **3** (12.1 mg, 34.5 µmol, 1.00 equiv.) was suspended in toluene (1 mL) and $SiMe_3Cl$ (4.5 µL, 35 µmol, 1.0 equiv.) was added at –40 °C. The solution was warmed up to RT and stirred for 30 min. The resulting suspension was filtered and the solvent of the filtrate was removed under reduced pressure. The product was then extracted with pentane (5 mL), after which the extract was filtered. The volatiles of the filtrate were evaporated under reduced pressure, affording **5** as a beige solid (5.3 mg, 37%).

^{1}H NMR (400 MHz, C_6D_6) δ 6.78 (s, 2H, CH_{arom}, Mes), 6.75 (s, 2H, CH_{arom}, Mes), 6.09 (s, 1H, $CH_{imidazole}$), 2.18 (d, J = 1.5 Hz, 12H, CH_3, Mes), 2.12 (s, 3H, CH_3, Mes), 2.07 (s, 3H, CH_3, Mes), -0.16 (s, 9H, $SiMe_3$).

^{13}C{^{1}H} NMR (101 MHz, C_6D_6) δ 152.2 (<u>C</u>=CN2), 139.4 (C_q, Mes), 139.2 (C_q, Mes), 137.3 (C_q, Mes), 136.5 (C_q, Mes), 134.0 (C_q, Mes), 133.5 (C_q, Mes), 129.3 (CH_{arom}, Mes), 129.3 (CH_{arom}, Mes), 128.2 (<u>C_q</u>–Si), 125.5 ($CH_{imidazole}$), 33.2 (<u>C</u>N_2), 21.2 (CH_3, Mes), 21.2 (CH_3, Mes), 18.2 (CH_3, Mes), 18.0 (CH_3, Mes), –1.4 ($SiMe_3$).

FT-IR (solid, ν, cm^{-1}): 1966 (N_2).

HRMS (nanochip-ESI/LTQ-Orbitrap) *m/z*: $[M + H]^+$ Calcd for $C_{25}H_{33}N_4Si^+$ 417.2469; Found 417.2480.

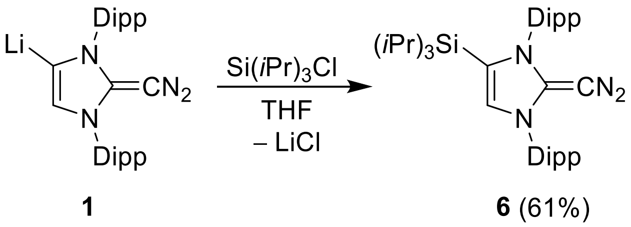

Compound 6: Diazoolefin **1** (20.0 mg, 46.0 μmol, 1.00 equiv.) was dissolved in THF (2 mL) and Si(*i*Pr$_3$)Cl (10.2 μL, 46.3 μmol, 1.01 equiv.) was added at –40 °C. The solution was stirred for 3 h. The solvent was removed under reduced pressure, after which the product was extracted with pentane (2 x 3 mL). The pentane extracts were combined and the solvent was removed under reduced pressure, affording **6** as a light-yellow solid (16.5 mg, 61%).

^{1}H NMR (600 MHz, C_6D_6) δ 7.27 (dt, *J* = 7.8, 3.7 Hz, 2H, CH_{arom}, Dipp), 7.13 (d, *J* = 7.7 Hz, 2H, CH_{arom}, Dipp), 7.10 (d, *J* = 7.7 Hz, 2H, CH_{arom}, Dipp), 6.45 (s, 1H, CH, imidazole), 3.08 (hept, *J* = 6.9 Hz, 2H, C<u>H</u>(CH_3)$_2$, Dipp), 3.00 (hept, *J* = 6.8 Hz, 2H, C<u>H</u>(CH_3)$_2$, Dipp), 1.58 (d, *J* = 6.8 Hz, 6H, CH_3, Dipp), 1.42 (d, *J* = 6.8 Hz, 6H, CH_3, Dipp), 1.27 (d, *J* = 6.8 Hz, 6H, CH_3, Dipp), 1.21 (d, *J* = 6.9 Hz, 6H, CH_3, Dipp), 0.98 (hept, 3H, C<u>H</u>(CH_3)$_2$, Si(*i*Pr$_3$)), 0.88 (d, *J* = 7.3 Hz, 18H, CH_3, Si(*i*Pr$_3$)).
^{13}C{^{1}H} NMR (151 MHz, C_6D_6) δ 154.2 (<u>C</u>=CN_2), 148.0 (C_q, Dipp), 147.3 (C_q, Dipp), 135.1 (C_q, Dipp), 133.2 (C_q, Dipp), 130.7 (CH_{arom}, Dipp), 130.5 (CH_{arom}, Dipp), 128.6 (CH, imidazole), 125.6 (C_q–Si), 124.4 (CH_{arom}, Dipp), 124.2 (CH_{arom}, Dipp), 35.8 (CN_2), 29.4 (C<u>H</u>(CH_3)$_2$, Dipp), 29.2 (C<u>H</u>(CH_3)$_2$, Dipp), 25.3 (CH_3, Dipp), 24.4 (CH_3, Dipp), 23.8 (CH_3, Dipp), 23.1 (CH_3, Dipp), 18.6 (CH_3, Si(*i*Pr$_3$)), 11.9 (<u>C</u>H(CH_3)$_2$, Si(*i*Pr$_3$)).
FT-IR (solid, *ν*, cm^{-1}): 1969 (N_2).
HRMS (nanochip-ESI/LTQ-Orbitrap) *m/z*: $[M + H]^+$ Calcd for $C_{37}H_{57}N_4Si^+$ 585.4347; Found 585.4347.

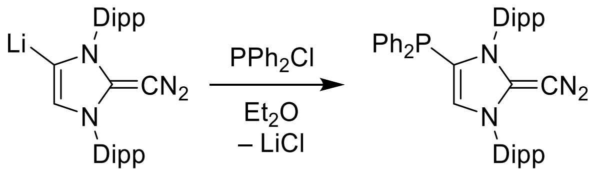

1 **7** (69%)

Compound 7: A solution of chlorodiphenylphosphine (8.3 µL, 46 µmol, 1.0 equiv.) in diethyl ether (1 mL) was added dropwise to a solution of diazoolefin **1** (20.0 mg, 46.0 µmol, 1.00 equiv.) in diethyl ether (2 mL). The mixture was stirred vigorously for 15 min, and then it was filtered. The filtrate was collected and the solvent was evaporated under reduced pressure. The crude product was washed with pentane (5 × 3 mL) and dried under reduced pressure to afford **7** as a beige powder (19 mg, 69%).

Crystals, suitable for single-crystal XRD analysis, were obtained by placing a concentrated diethyl ether solution of **7** in a freezer at –40 °C. These crystals appeared orange in color.

^{1}H NMR (400 MHz, C_6D_6): δ 7.36 – 7.30 (m, 4H, CH_{arom}, Ph), 7.30 – 7.21 (m, 2H, CH_{arom}, Dipp), 7.12 (d, *J* = 7.7 Hz, 2H, CH_{arom}, Dipp), 7.08 (d, *J* = 7.8 Hz, 2H, CH_{arom}, Dipp), 7.04 – 6.95 (m, 6H, CH_{arom}, Ph), 6.14 (s, 1H, $CH_{imidazole}$), 3.09 (hept, *J* = 6.9 Hz, 2H, $C\underline{H}(CH_3)_2$, Dipp), 2.95 (hept, *J* = 6.8 Hz, 2H, $C\underline{H}(CH_3)_2$, Dipp), 1.51 (d, *J* = 6.8 Hz, 6H, CH_3, Dipp), 1.42 (d, *J* = 6.8 Hz, 6H, CH_3, Dipp), 1.12 (d, *J* = 6.9 Hz, 6H, CH_3, Dipp), 1.01 (d, *J* = 6.8 Hz, 6H, CH_3, Dipp).
^{13}C{^{1}H} NMR (101 MHz, C_6D_6): δ 153.7 ($\underline{C}=CN_2$), 148.2 (C_q, Dipp), 147.0 (C_q, Dipp), 135.3 (d, *J* = 8.4 Hz, C_q, Ph), 133.6 (C_q, Dipp), 133.4 (C_q, Dipp), 131.1 (CH_{arom}, Dipp), 130.5 (CH_{arom}, Dipp), 129.5 (CH_{arom}, Ph), 129.0 (d, *J* = 7.0 Hz, CH_{arom}, Ph), 128.8 (d, *J* = 7.3 Hz, C_q, imidazole), 128.6 (CH_{arom}, Ph), 126.5 (d, *J* = 4.5 Hz, $CH_{imidazole}$), 124.3 (CH_{arom}, Dipp), 124.2 (CH_{arom}, Dipp), 36.7 (CN_2), 29.8 ($\underline{C}H(CH_3)_2$), 29.4 ($\underline{C}H(CH_3)_2$), 24.8 (CH_3, Dipp), 24.1 (CH_3, Dipp), 23.7 (CH_3, Dipp), 23.1 (CH_3, Dipp).
{^{1}H}^{31}P NMR (162 MHz, C_6D_6) δ –38.5 (s).
FT-IR (solid, *ν*, cm^{-1}): 1977 (N_2).
HRMS (ESI/QTOF) *m*/*z*: $[M + H]^+$ Calcd for $C_{40}H_{46}N_4P^+$ 613.3460; Found 613.3483.

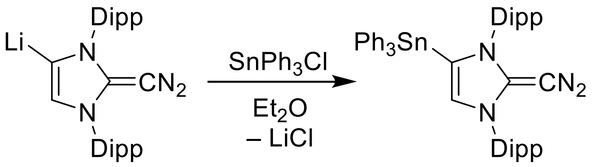

1 **8** (65%)

Compound 8: Diazoolefin **1** (9.9 mg, 23 µmol, 1.0 equiv.) was dissolved in diethyl ether (2 mL), and the solution was added to a suspension of triphenyltin chloride (9.0 mg, 23 µmol, 1.0 equiv.) in diethyl ether (2 mL) at –40 °C. The mixture was stirred for 2 h, after which it was filtered. The solvent of the filtrate was removed under reduced pressure and the remaining solids were washed with pentane (1 x 2 mL). The solids were dried under reduced pressure, affording compound **8** as a beige solid (12 mg, 65%).

Crystals, suitable for single-crystal XRD analysis, were obtained by placing a concentrated diethyl ether solution of **8** in a freezer at –40 °C. These crystals appeared yellow in color.

^{1}H NMR (500 MHz, C_6D_6) δ 7.25 (dt, *J* = 6.5, 1.7 Hz, 5H, CH_{arom}, Ph), 7.19 (t, *J* = 7.7 Hz, 2H, CH_{arom}, Dipp), 7.14 – 7.01 (m, 12H, CH_{arom}, Dipp and Ph), 6.95 (d, *J* = 7.8 Hz, 2H, CH_{arom}, Dipp), 6.38 (s, 1H, $CH_{imidazole}$), 3.14 (hept, *J* = 7.0 Hz, 2H, C<u>H</u>(CH_3)$_2$, Dipp), 3.02 (hept, *J* = 6.9 Hz, 2H, C<u>H</u>(CH_3)$_2$, Dipp), 1.46 (d, *J* = 6.9 Hz, 6H, CH_3, Dipp), 1.44 (d, *J* = 6.9 Hz, 6H, CH_3, Dipp), 1.21 (d, *J* = 6.9 Hz, 6H, CH_3, Dipp), 0.67 (d, *J* = 6.9 Hz, 6H, CH_3, Dipp).
^{13}C{^{1}H} NMR (126 MHz, C_6D_6) δ 154.6 (<u>C</u>=CN_2), 147.8 (C_q, Dipp), 147.1 (C_q, Dipp), 137.2 (CH_{arom}, Dipp), 136.0 (C_q, Ph), 135.1 (C_q, Dipp), 133.3 (C_q, Dipp), 130.6 (CH_{arom}, Dipp), 130.3 (CH_{arom}, Dipp), 129.9 (CH_{arom}, Ph), 129.1 (CH_{arom}, Ph), 129.0 ($CH_{imidazole}$), 126.9 ($C_{imidazole}$–Sn), 124.5 (CH_{arom}, Dipp), 124.2 (CH_{arom}, Dipp), 35.9 (CN_2), 29.4 (<u>C</u>H(CH_3)$_2$, Dipp), 29.3 (<u>C</u>H(CH_3)$_2$, Dipp), 24.6 (CH_3, Dipp), 24.2 (CH_3, Dipp), 23.8 (CH_3, Dipp), 22.6 (CH_3, Dipp).
FT-IR (solid, *ν*, cm^{-1}): 1975 (N_2).
HRMS (ESI/QTOF) *m/z*: $[M + H]^+$ Calcd for $C_{46}H_{51}N_4Sn^+$ 779.3130; Found 779.3158.

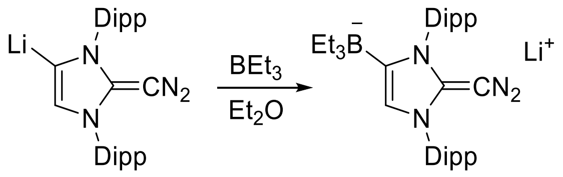

1 **9** (81%)

Compound 9: Compound **1** (15.0 mg, 34.5 μmol, 1.00 equiv.) was dissolved in diethyl ether (3 mL) and triethylborane (1 M in THF, 60.0 μL, 60.0 μmol, 1.74 equiv.) was added while stirring. The mixture was then stirred for 4 h at RT. The volatiles were evaporated under reduced pressure, and the remaining solids were washed with pentane (3 x 1 mL) to afford **9** as an orange solid (14.7 mg, 81%).

Crystals, suitable for single-crystal XRD analysis, were obtained by placing a concentrated diethyl ether solution of **9** in a freezer at –40 °C. These crystals appeared light-yellow in color.

^{1}H NMR (500 MHz, THF) δ 7.29 (t, *J* = 7.7 Hz, 1H, CH_{arom}, Dipp), 7.20 (t, *J* = 7.7 Hz, 1H, CH_{arom}, Dipp), 7.17 (d, *J* = 7.7 Hz, 2H, CH_{arom}, Dipp), 7.07 (d, *J* = 7.7 Hz, 2H, CH_{arom}, Dipp), 6.17 (s, 1H, $CH_{imidazole}$), 3.64 – 3.59 (m, 4H, THF), 2.96 (hept, *J* = 6.9 Hz, 4H, C<u>H</u>$(CH_3)_2$, Dipp), 1.81 – 1.74 (m, 4H, THF), 1.31 (d, *J* = 6.7 Hz, 6H, CH_3, Dipp), 1.26 (d, *J* = 6.8 Hz, 6H, CH_3, Dipp), 1.23 (d, *J* = 6.9 Hz, 6H, CH_3, Dipp), 1.19 (d, *J* = 6.9 Hz, 6H, CH_3, Dipp), 0.57 (t, *J* = 7.6 Hz, 9H, CH_3, BEt_3), -0.10 (q, *J* = 7.7 Hz, 6H, CH_2, BEt_3).

^{13}C{^{1}H} NMR (126 MHz, THF) δ 148.2 (C_q, Dipp), 147.6 (C_q, Dipp), 146.2 (<u>C</u>=CN_2), 137.9 (C_q, Dipp), 135.2 (C_q, Dipp), 129.4 (CH_{arom}, Dipp), 128.7 (CH_{arom}, Dipp), 123.7 (CH_{arom}, Dipp), 123.0 (CH_{arom}, Dipp), 121.4 ($CH_{imidazole}$), 68.0 (THF), 29.0 (<u>C</u>H$(CH_3)_2$, Dipp), 29.0 (<u>C</u>H$(CH_3)_2$, Dipp), 26.2 (THF), 25.5 (CH_3, Dipp), 24.4 (CH_3, Dipp), 24.0 (CH_3, Dipp), 23.4 (CH_3, Dipp), 22.1 (CN_2), 15.5 (broad, CH_2, BEt_3), 11.3 (CH_3, BEt_3). *The carbon peak (C_q–B) was not observed due to broadening as a result of the coupling with boron.*

^{11}B NMR (128 MHz, THF) δ -15.47.

FT-IR (solid, *ν*, cm^{-1}): 2085 (N_2).

HRMS (ESI/QTOF) *m/z*: $[M]^-$ Calcd for $C_{34}H_{50}BN_4^-$ 525.4134; Found 525.4123.

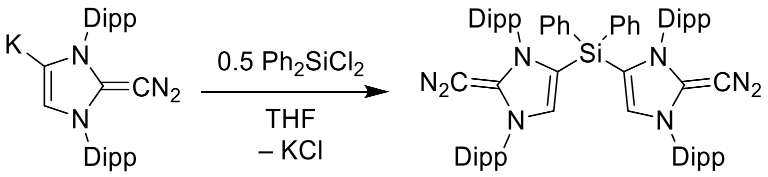

2 **10** (71%)

Compound 10: Compound **2** (30.0 mg, 49.1 µmol, 1.00 equiv.) was dissolved in THF (1 mL) and the solution was cooled to –40 °C. A cold solution of dichlorodiphenylsilane (5.2 µL, 25 µmol, 0.51 equiv.) in THF (1 mL) was added dropwise and the mixture was stirred at –40 °C for 15 min, warmed up to RT, and stirred for another 15 min. The resulting suspension was filtered and the filtrate was evaporated under reduced pressure. After extraction with diethyl ether and removal of solvents under reduced pressure, the crude product was washed with pentane (5 × 2 mL) and dried under reduced pressure to afford **10** as dark-yellow powder (18 mg, 71%).

Crystals, suitable for single-crystal XRD analysis, were grown by layering a THF solution of **10** with pentane. These crystals appeared light-yellow in color.

^{1}H NMR (400 MHz, C_6D_6) δ 7.27 (t, *J* = 7.7 Hz, 2H, CH_{arom}, Dipp), 7.21 (s, 2H, $CH_{imidazole}$), 7.14 – 7.09 (m, 8H, CH_{arom}, Dipp, Ph), 7.00 – 6.92 (m, 4H, CH_{arom}, Dipp and Ph), 6.85 (t, *J* = 7.5 Hz, 4H, CH_{arom}, Ph), 6.68 (d, *J* = 7.8 Hz, 4H, CH_{arom}, Dipp), 3.17 (hept, *J* = 6.9 Hz, 4H, $C\underline{H}(CH_3)_2$), 2.75 (hept, *J* = 6.8 Hz, 4H, $C\underline{H}(CH_3)_2$), 1.39 (d, *J* = 6.8 Hz, 12H, CH_3), 1.39 (d, *J* = 6.9 Hz, 12H, CH_3), 1.17 (d, *J* = 7.0 Hz, 12H, CH_3), 0.57 (d, *J* = 6.8 Hz, 12H, CH_3).
^{13}C{^{1}H} NMR (101 MHz, C_6D_6) δ 155.1 ($\underline{C}$=CN_2), 147.4 (C_q, Dipp), 147.2 (C_q, Dipp), 136.7 (C_q, Ph), 133.4 (C_q, Dipp), 132.8 (C_q, Dipp), 131.2, 130.9, 130.6, 130.4, 128.7, 128.6, 124.4 (CH_{arom}, Ph), 123.9 (CH_{arom}, Dipp), 122.8 ($CH_{imidazole}$), 37.9 (CN_2), 29.4 ($\underline{C}H(CH_3)_2$), 29.4 ($\underline{C}H(CH_3)_2$), 25.2 (CH_3), 24.4 (CH_3), 23.7 (CH_3), 22.3 (CH_3). *We were not able to assign all the peaks due to overlap in the 2d spectra.*
FT-IR (solid, ν, cm^{-1}): 1974 (N_2).
HRMS (ESI/QTOF) *m/z*: $[M + H]^+$ Calcd for $C_{68}H_{81}N_8Si^+$ 1037.6348; Found 1037.6304.

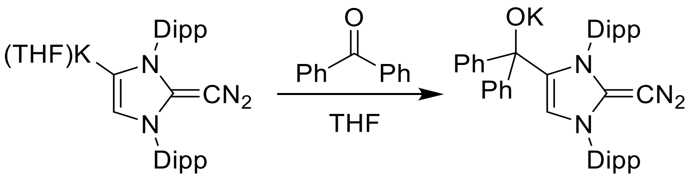

2 **11** (83%)

Compound 11: Diazoolefin **2** (19.0 mg, 35.3 µmol, 1.00 equiv.) and benzophenone (7.4 mg, 41 µmol, 1.2 equiv.) were combined in THF (1.5 mL). The solution was stirred at RT for 15 min, followed by removal of the solvent under reduced pressure. The crude product was washed with diethyl ether (3 × 2 mL) and pentane (2 × 2 mL), and the residue was dried under reduced pressure to afford **11** as beige powder (19 mg, 83%).

Crystals, suitable for single-crystal XRD analysis, were grown by slow vapor diffusion of diethyl ether onto a solution of **11** in THF. These crystals appeared orange in color.

^{1}H NMR (400 MHz, *d_8*-THF) δ 7.33 (d, *J* = 7.6 Hz, 4H, CH_{arom}, Ph), 7.29 (t, *J* = 7.7 Hz, 1H, CH_{arom}, Dipp), 7.22 (t, *J* = 7.6 Hz, 1H, CH_{arom}, Dipp), 7.18 – 7.05 (m, 6H, CH_{arom}, Ph), 7.16 (d, *J* = 7.8 Hz, 2H, CH_{arom}, Dipp), 7.15 (d, *J* = 7.6 Hz, 2H, CH_{arom}, Dipp), 5.59 (s, 1H, $CH_{imidazole}$), 3.02 (hept, *J* = 6.9 Hz, 2H, $C\underline{H}(CH_3)_2$), 2.77 (hept, *J* = 6.9 Hz, 2H, $C\underline{H}(CH_3)_2$), 1.29 (d, *J* = 6.9 Hz, 6H, CH_3), 1.25 (d, *J* = 6.8 Hz, 6H, CH_3), 1.18 (d, *J* = 6.9 Hz, 6H, CH_3), 1.04 (d, *J* = 6.8 Hz, 6H, CH_3).

^{13}C{^{1}H} NMR (101 MHz, *d_8*-THF) δ 157.0 (C_q, Ph), 152.3 ($\underline{C}=CN_2$), 149.3 (C_q, Dipp), 147.9 (C_q, Dipp), 142.4 (C_q, imidazole), 137.7 (C_q, Dipp), 134.5 (C_q, Dipp), 130.4 (CH_{arom}, Dipp), 129.5 (CH_{arom}, Dipp), 128.6 (CH_{arom}, Ph), 128.0 (CH_{arom}, Ph), 126.3 (CH_{arom}, Ph), 124.3 (CH_{arom}, Dipp), 124.0 (CH_{arom}, Dipp), 118.6 ($CH_{imidazole}$), 84.3 ($C\text{-}O^-$), 33.2 (CN_2), 30.3 ($\underline{C}H(CH_3)_2$), 29.7 ($\underline{C}H(CH_3)_2$), 24.9 (CH_3), 24.6 (CH_3), 24.0 (CH_3). *Only three CH_3 signals are observed due to peak overlap.*

FT-IR (solid, *ν*, cm^{-1}): 1971 (N_2).

HRMS (ESI/QTOF) *m/z*: $[M - K + 2H]^+$ Calcd for $C_{41}H_{47}N_4O$ 611.3750; Found 611.3742. Anionic compound **11** could not be detected by mass spectrometry.

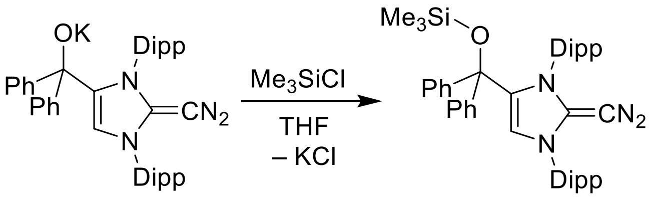

11 **12** (94%)

Compound 12: Compound **11** (20.0 mg, 25.2 µmol, 1.00 equiv.) was dissolved in THF (1.5 mL). Chlorotrimethylsilane (6.5 µL, 50 µmol, 2.0 equiv.) was added and the mixture was stirred for 30 min at RT. The volatiles were then removed under reduced pressure, and the residue was extracted with benzene. After filtration, the solvent was removed under reduced pressure. The crude product was washed with pentane (5 × 2 mL) and dried under reduced pressure to afford **12** as a brown powder (16 mg, 94%).

Crystals, suitable for single-crystal XRD analysis, were grown by slow vapor diffusion of pentane onto a solution of **12** in diethyl ether. These crystals appeared pale-yellow in color.

^{1}H NMR (400 MHz, C_6D_6) δ 7.37 – 7.33 (m, 4H, CH_{arom}, Ph), 7.31 (t, J = 7.8 Hz, 1H, CH_{arom}, Dipp), 7.23 (t, J = 7.7 Hz, 1H, CH_{arom}, Dipp), 7.13 (d, J = 7.7 Hz, 2H, CH_{arom}, Dipp), 7.08 (d, J = 7.7 Hz, 2H, CH_{arom}, Dipp), 7.06 – 7.02 (m, 6H, CH_{arom}, Ph), 5.90 (s, 1H, $CH_{imidazole}$), 3.07 (hept, J = 6.9 Hz, 2H, $C\underline{H}(CH_3)_2$), 2.71 (hept, J = 6.8 Hz, 2H, $C\underline{H}(CH_3)_2$), 1.54 (d, J = 6.8 Hz, 6H, CH_3, Dipp), 1.37 (d, J = 6.8 Hz, 6H, CH_3, Dipp), 1.15 (d, J = 6.8 Hz, 6H, CH_3, Dipp), 1.12 (d, J = 6.9 Hz, 6H, CH_3, Dipp), -0.35 (s, 9H, $Si(C\underline{H}_3)_3$).
^{13}C{^{1}H} NMR (101 MHz, C_6D_6) δ 153.0 ($\underline{C}=CN_2$), 147.8 (C_q, Dipp), 147.1 (C_q, Dipp), 144.1 (C_q, Ph), 136.4 (C_q, imidazole), 134.8 (C_q, Dipp), 133.3 (C_q, Dipp), 130.3 (CH_{arom}, Ph), 130.0 (CH_{arom}, Ph), 129.7 (CH_{arom}, Ph), 128.6 (CH_{arom}, Dipp), 128.5 (CH_{arom}, Dipp), 124.1 (CH_{arom}, Dipp), 124.1 (CH_{arom}, Dipp), 119.2 ($CH_{imidazole}$), 83.2 ($\underline{C}$-$OSiMe_3$), 36.9 (CN_2), 29.6 ($\underline{C}H(CH_3)_2$), 29.1 ($\underline{C}H(CH_3)_2$), 25.3 (CH_3, Dipp), 24.3 (CH_3, Dipp), 24.1 (CH_3, Dipp), 23.7 (CH_3, Dipp), 2.3 ($Si(\underline{C}H_3)_3$).
FT-IR (solid, ν, cm^{-1}): 1974 (N_2).
HRMS (ESI/QTOF) m/z: $[M + H]^+$ Calcd for $C_{44}H_{55}N_4OSi^+$ 683.4140; Found 683.4152.

3. NMR Data

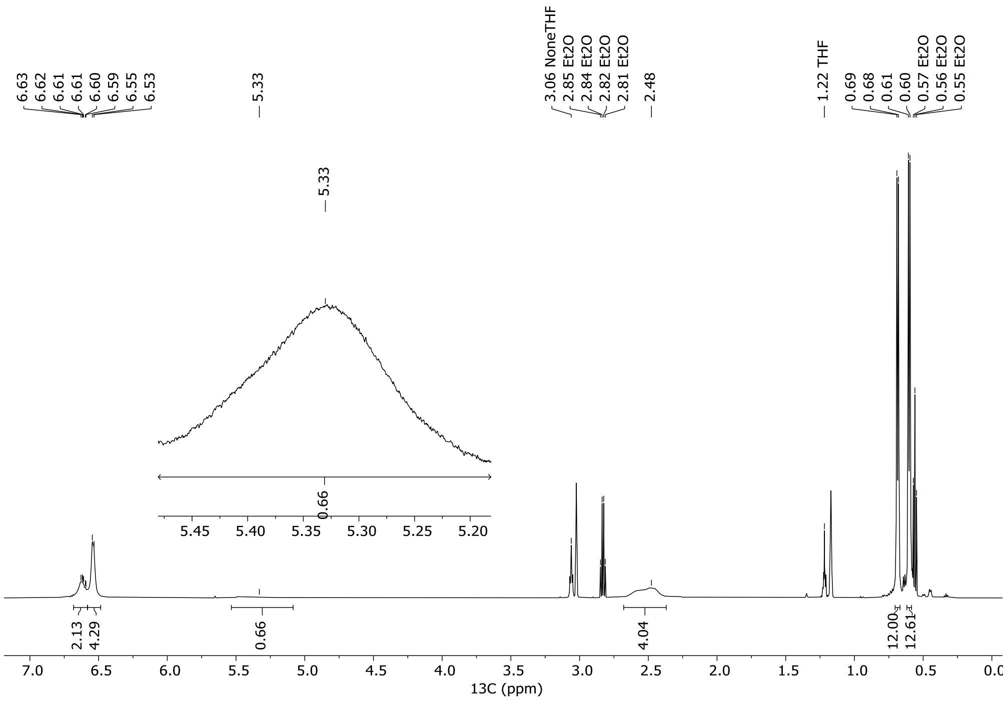

Figure S1. ^{1}H NMR (600 MHz, d_8-THF) spectrum of **2**.

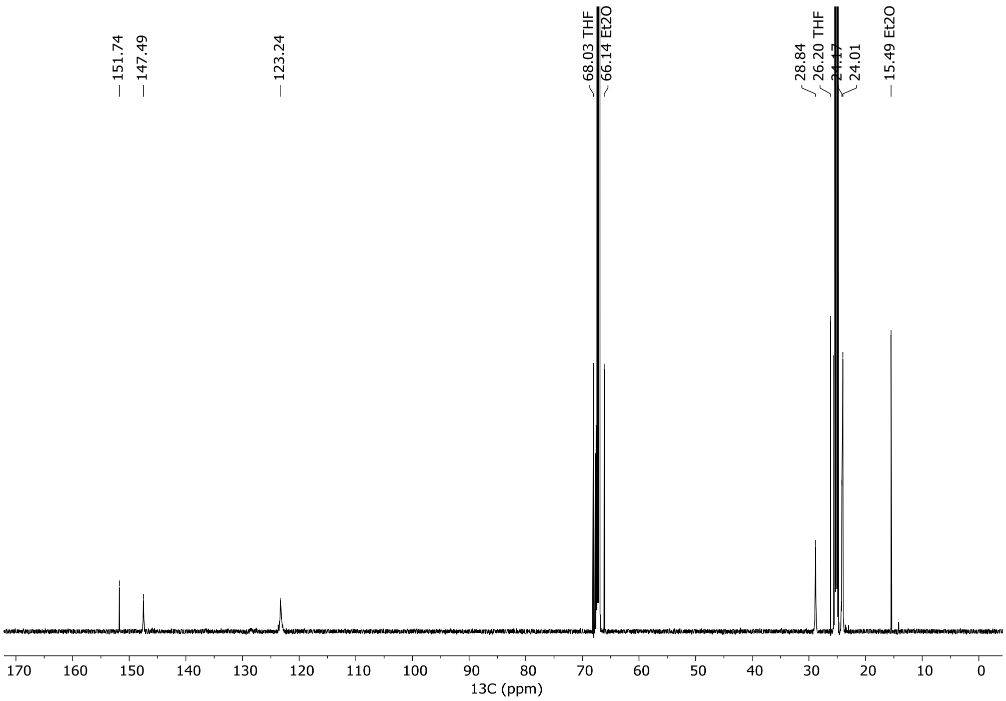

Figure S2. {^{1}H}^{13}C NMR (151 MHz, d_8-THF) spectrum of **2**.

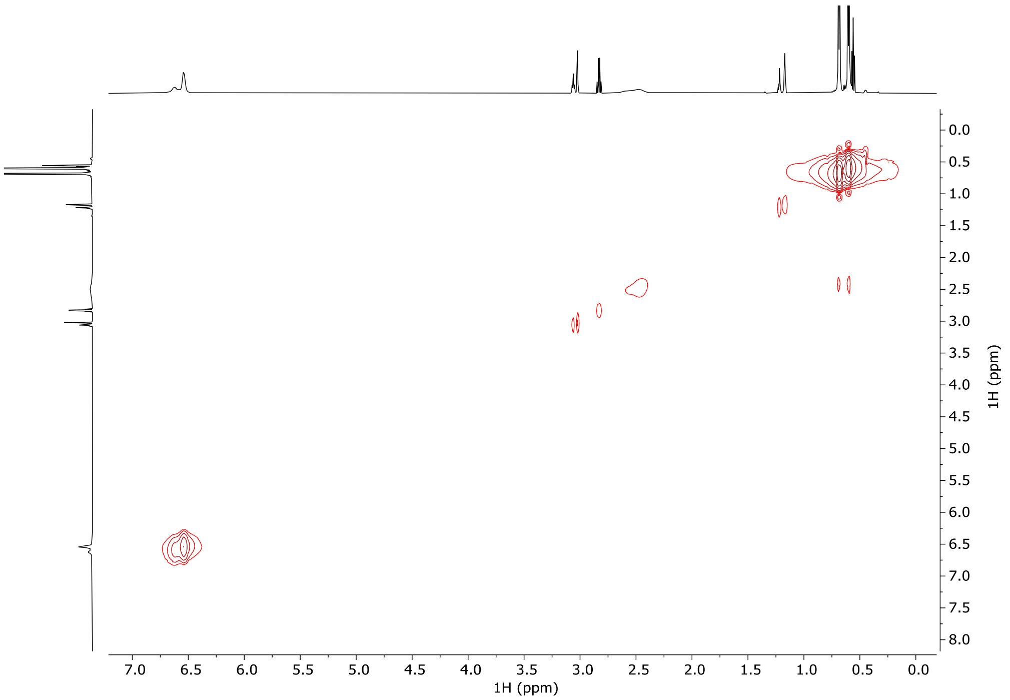

Figure **S3**. COSY NMR (d_8-THF) spectrum of **2**.

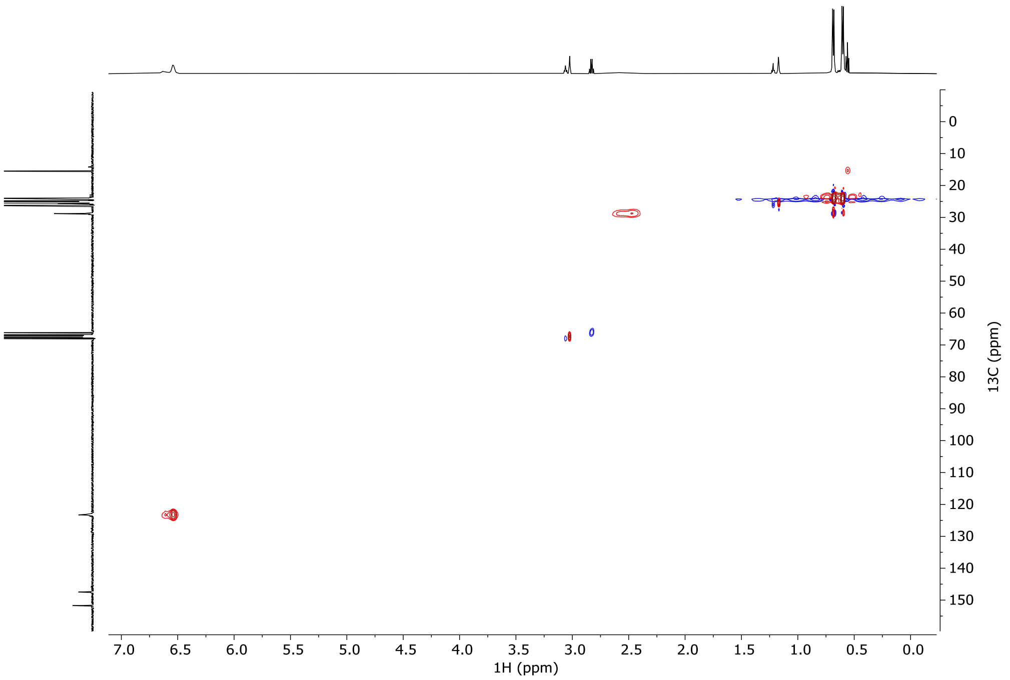

Figure **S4**. HSQC NMR (d_8-THF) spectrum of **2**.

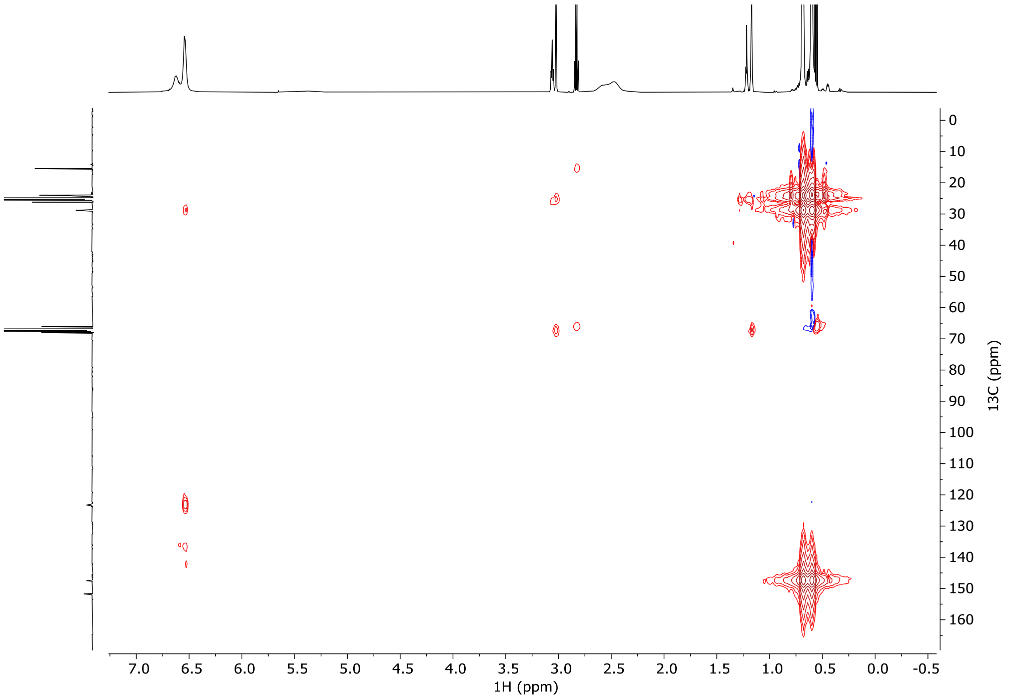

Figure **S5**. HMBC NMR (d_8-THF) spectrum of **2**.

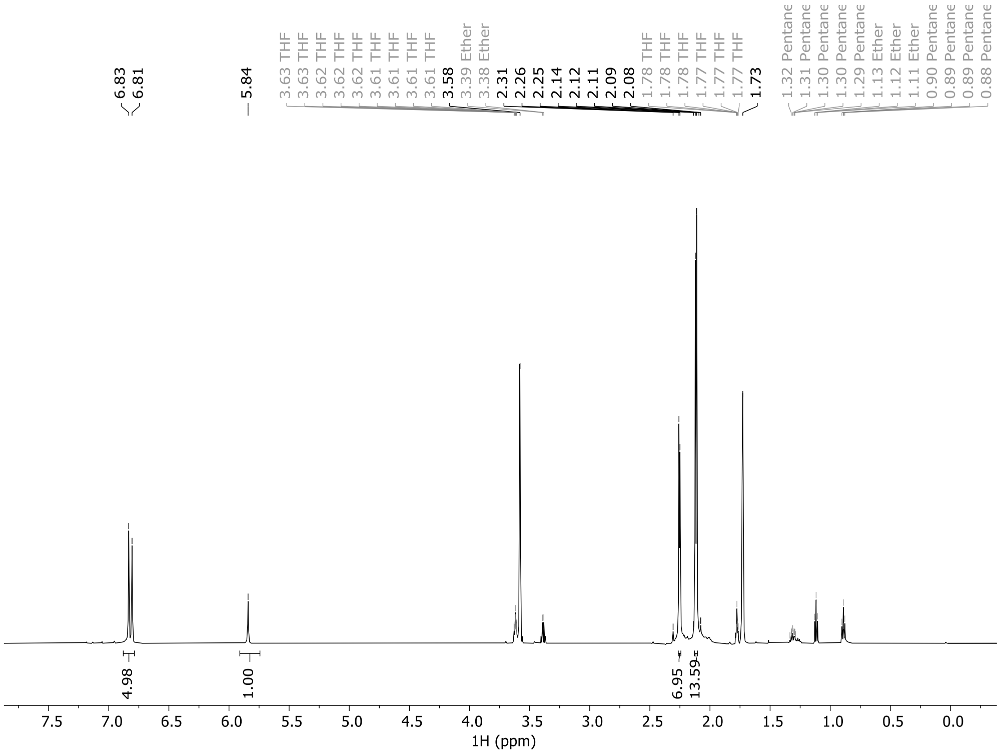

Figure S6. ¹H NMR (600 MHz, d_8-THF) spectrum of **3**.

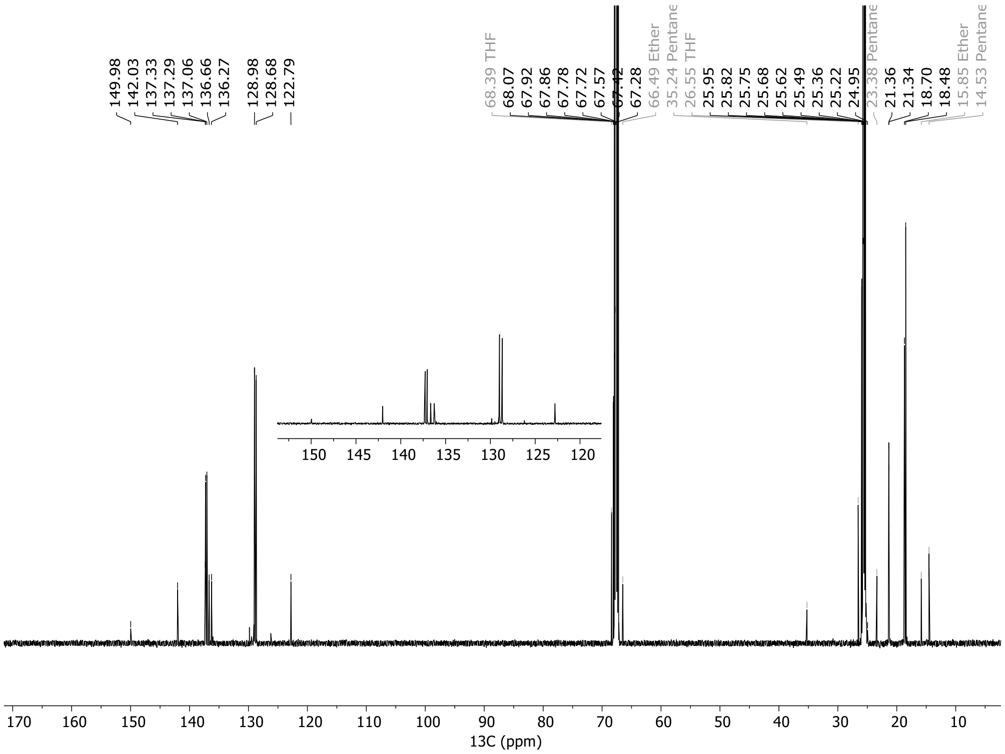

Figure S7. {^{1}H}^{13}C NMR (151 MHz, d_8-THF) spectrum of **3**.

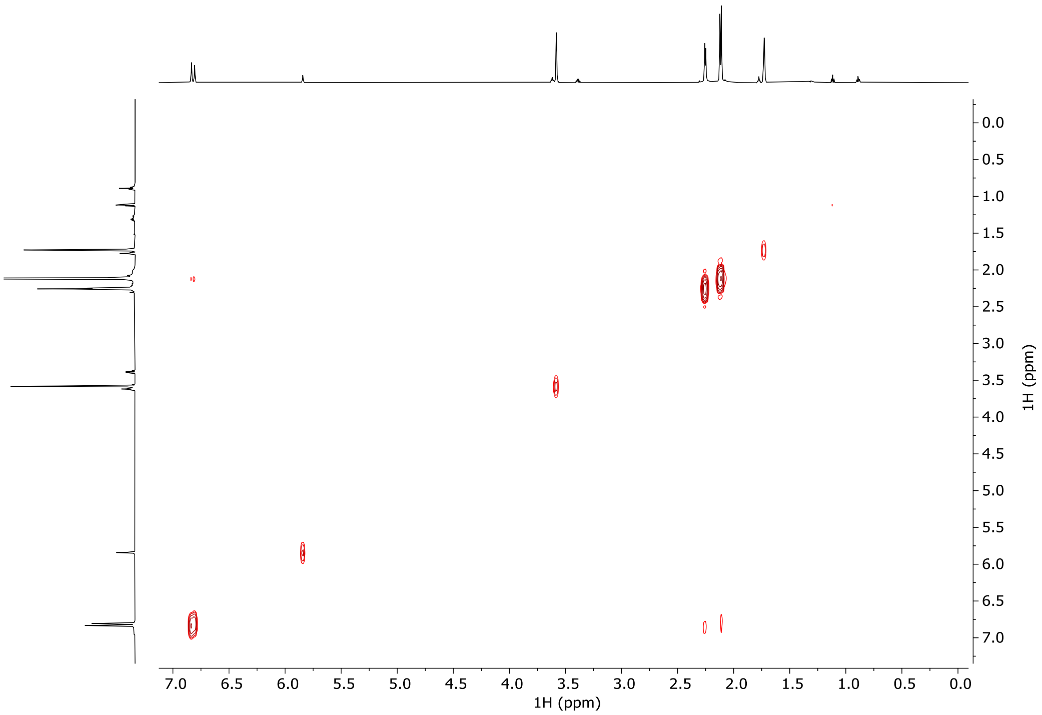

Figure **S8**. COSY NMR (d_8-THF) spectrum of **3**.

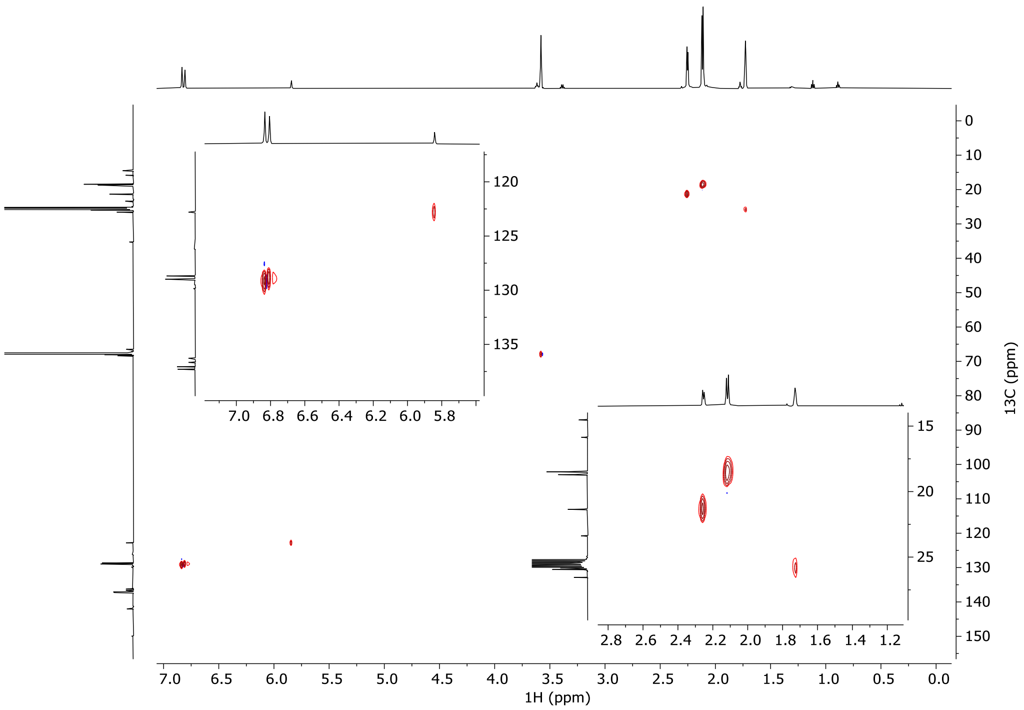

Figure **S9**. HSQC NMR (d_8-THF) spectrum of **3**.

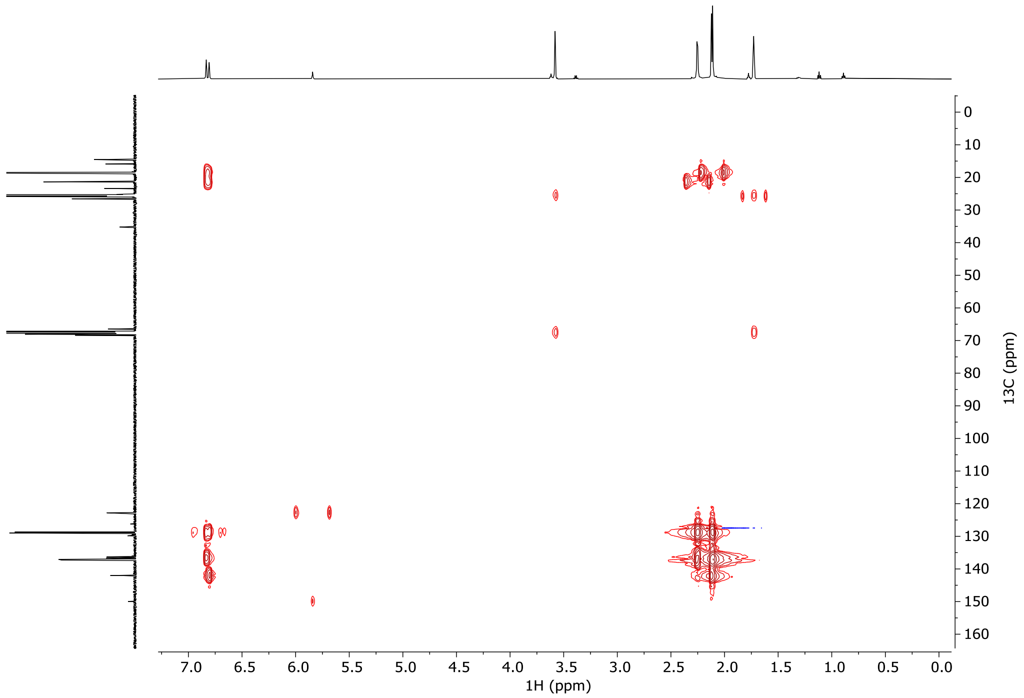

Figure **S10**. HMBC NMR (d_8-THF) spectrum of **3**.

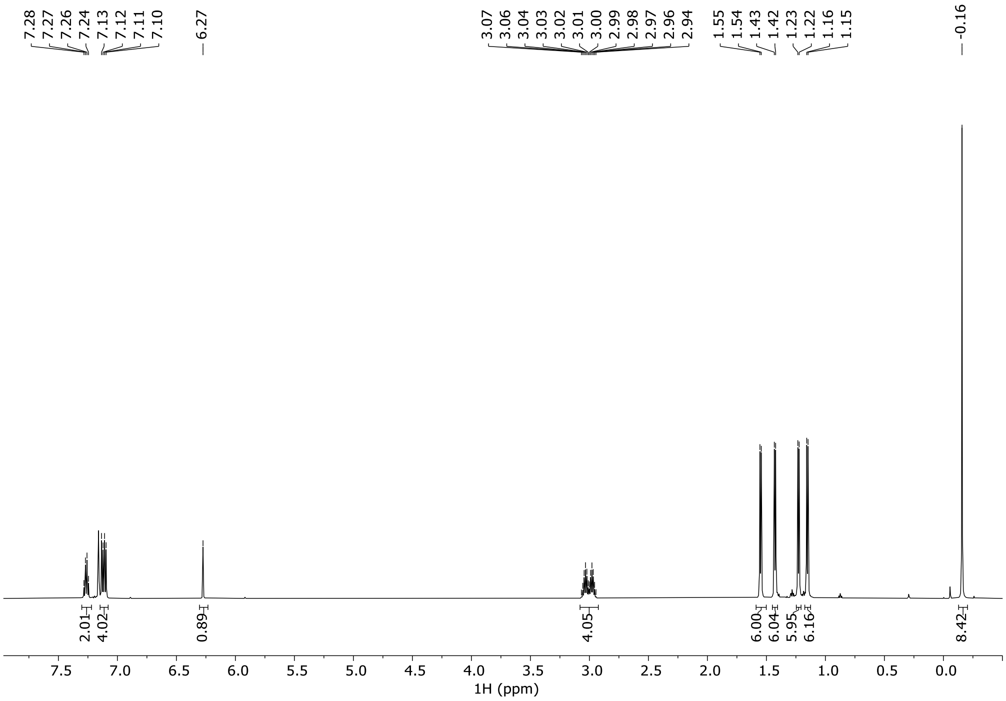

Figure S11. ^{1}H NMR (600 MHz, C_6D_6) spectrum of **4**.

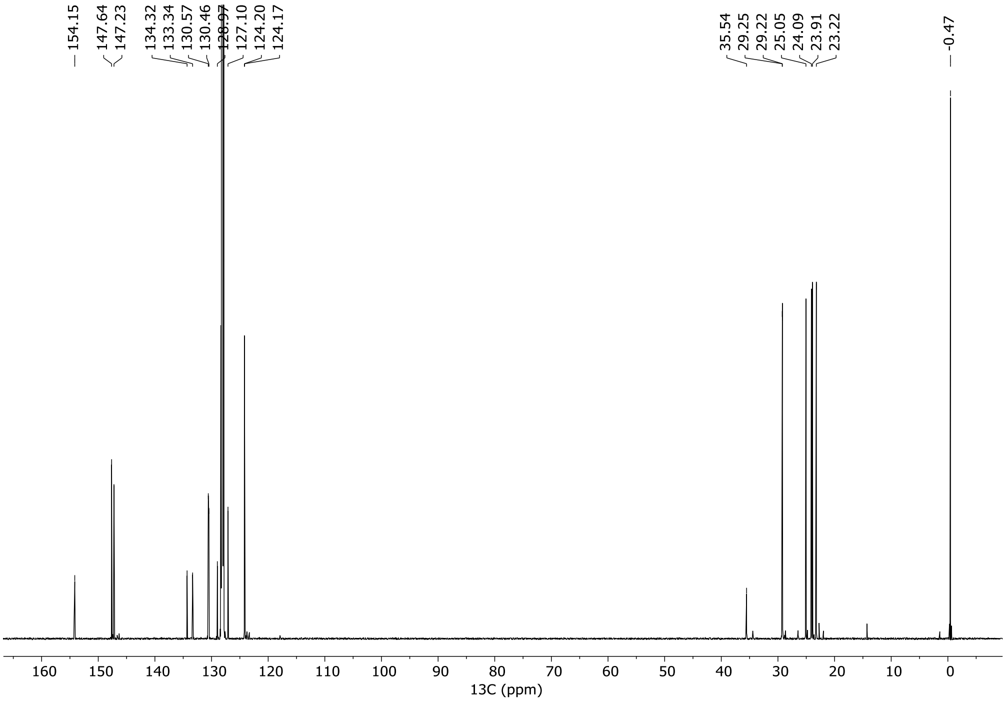

Figure S12. {^{1}H}^{13}C NMR (151 MHz, C_6D_6) spectrum of **4**.

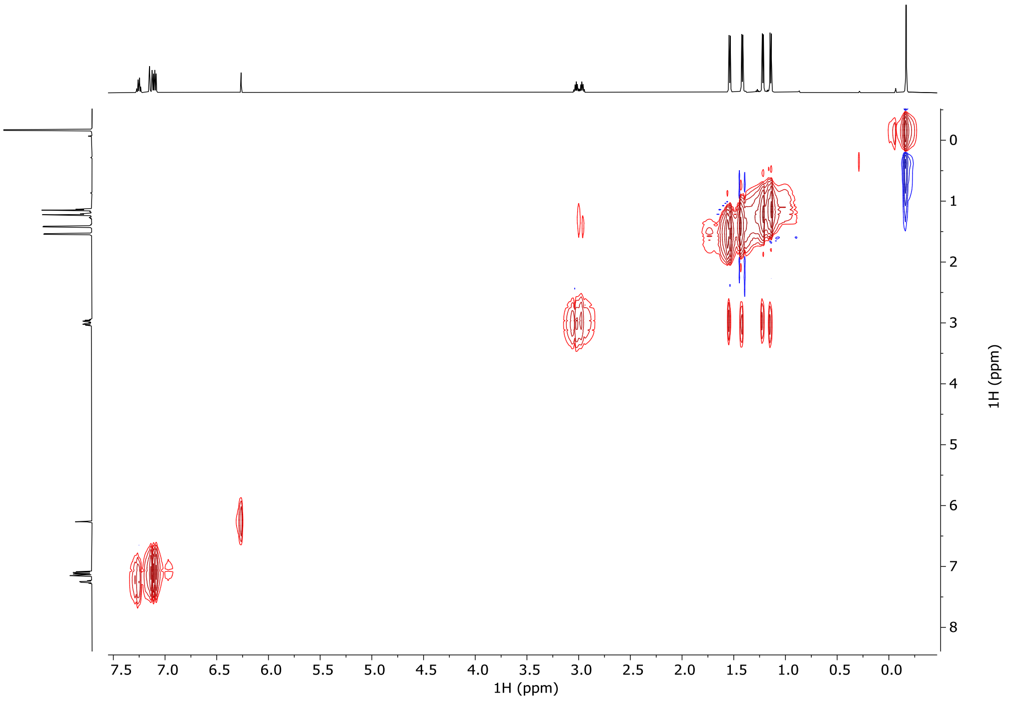

Figure S13. COSY NMR (C_6D_6) spectrum of **4**.

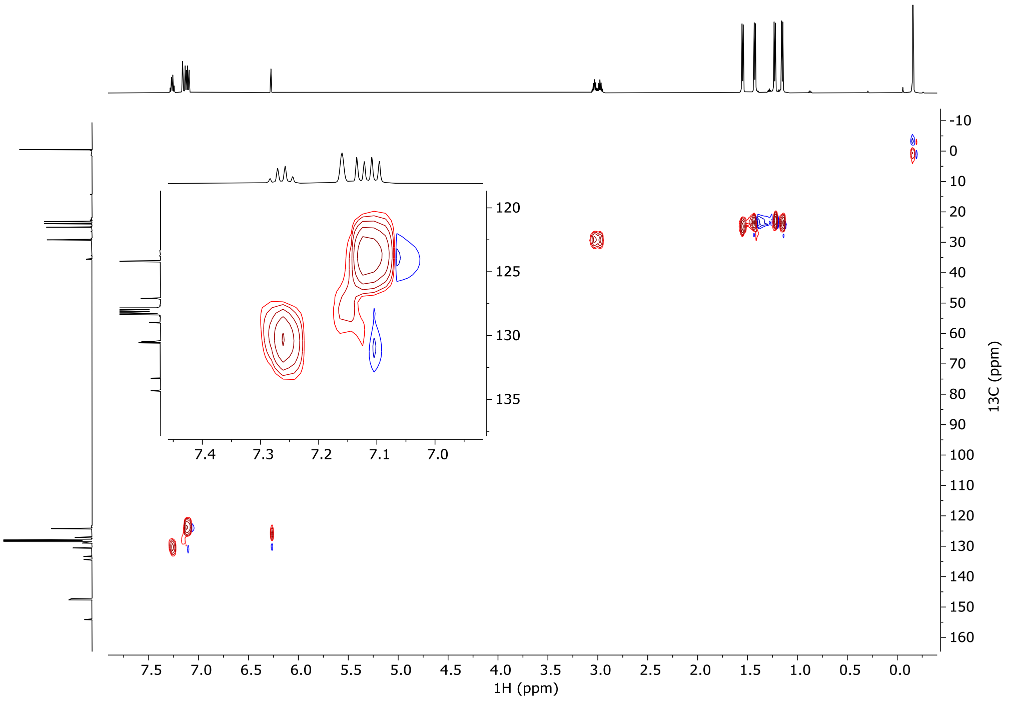

Figure S14. HSQC NMR (C_6D_6) spectrum of **4**.

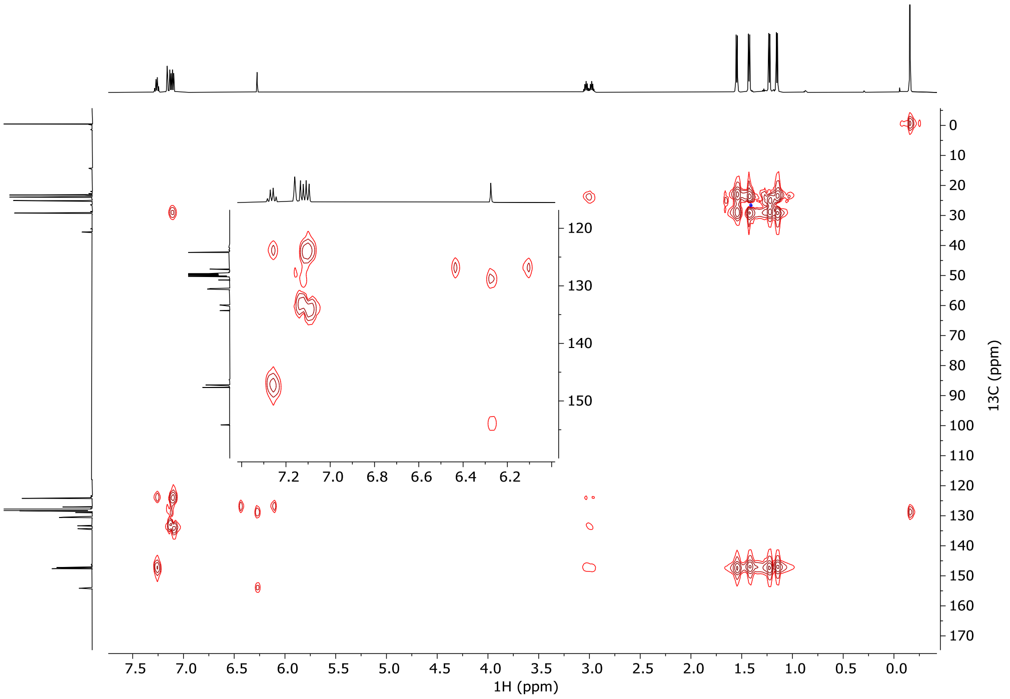

Figure S15. HMBC NMR (C_6D_6) spectrum of **4**.

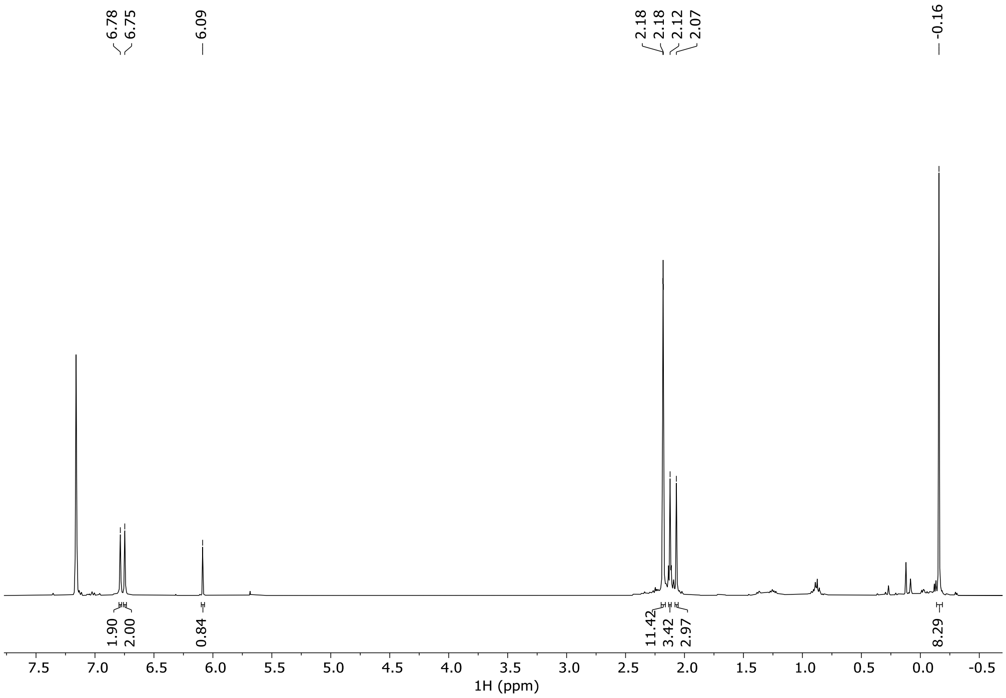

Figure S16. ^{1}H NMR (400 MHz, C_6D_6) spectrum of **5**.

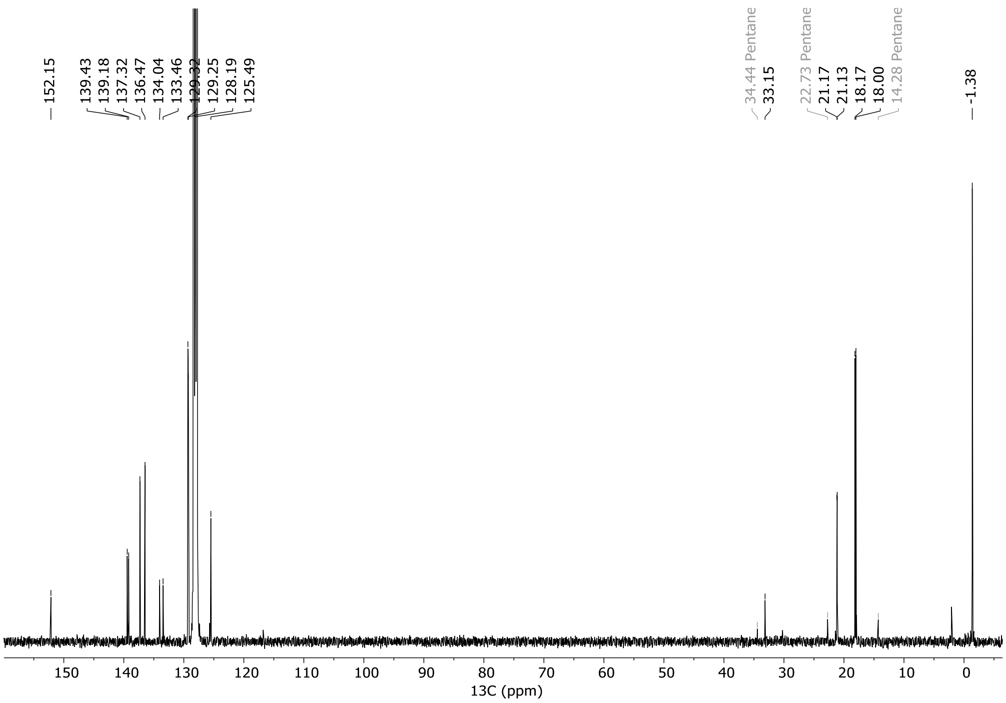

Figure S17. {^{1}H}^{13}C NMR (101 MHz, C_6D_6) spectrum of **5**.

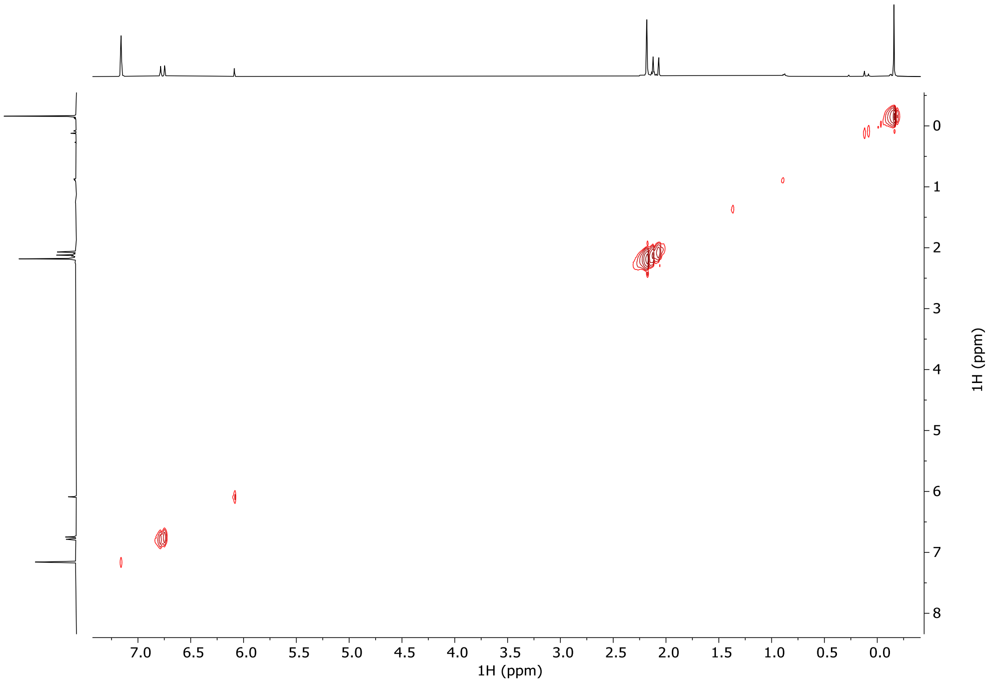

Figure S18. COSY NMR (400 MHz, C_6D_6) spectrum of **5**.

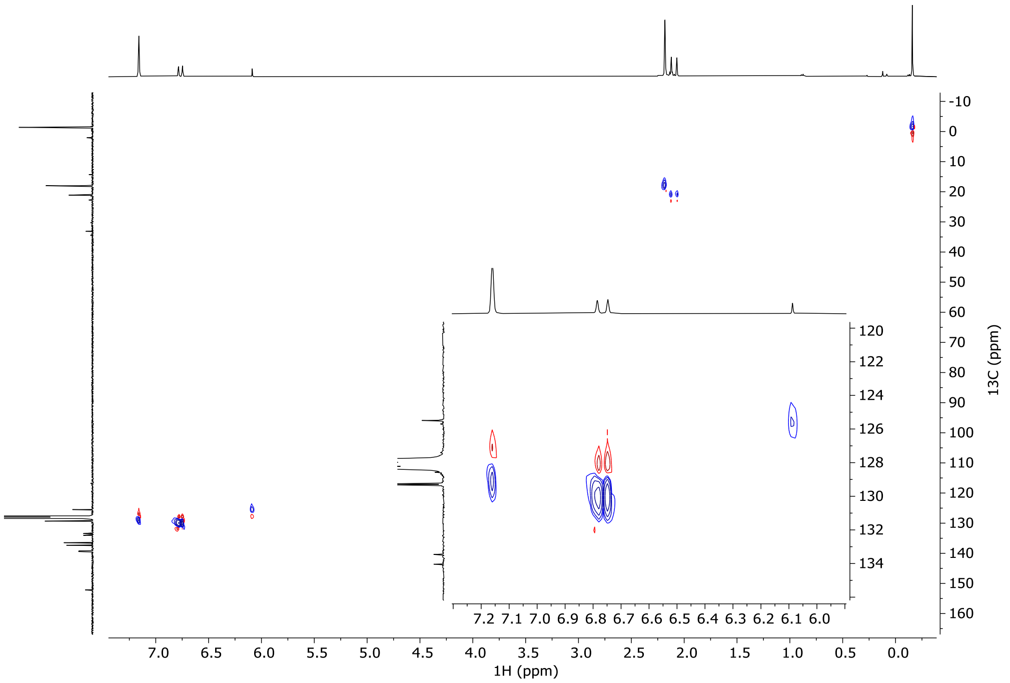

Figure S19. HSQC NMR (400 MHz, C_6D_6) spectrum of **5**.

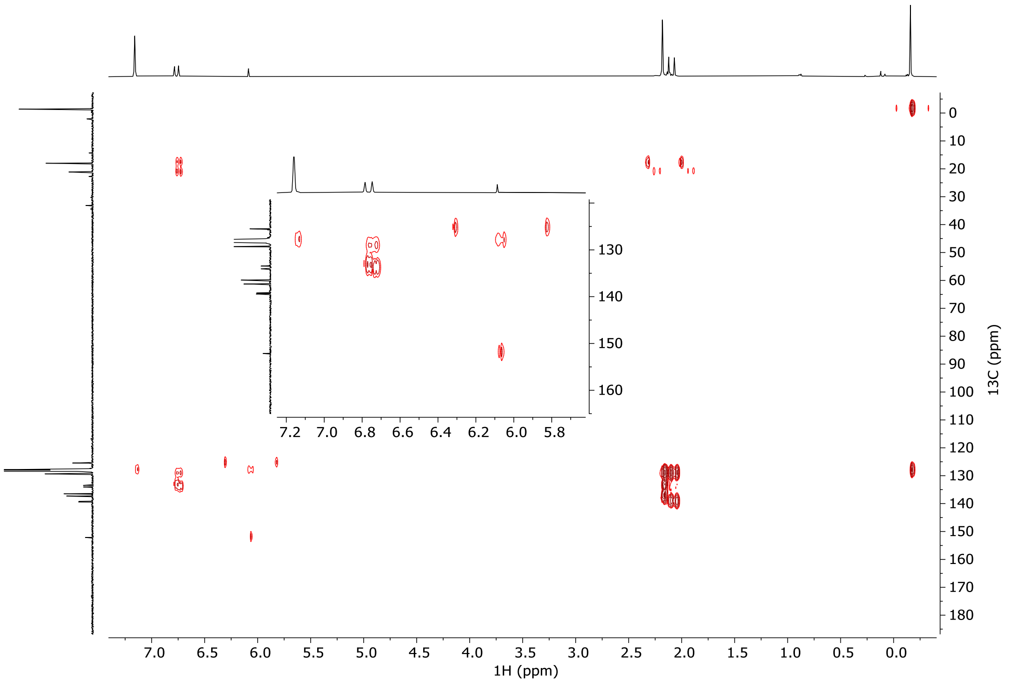

Figure S20. HMBC NMR (400 MHz, C_6D_6) spectrum of **5**.

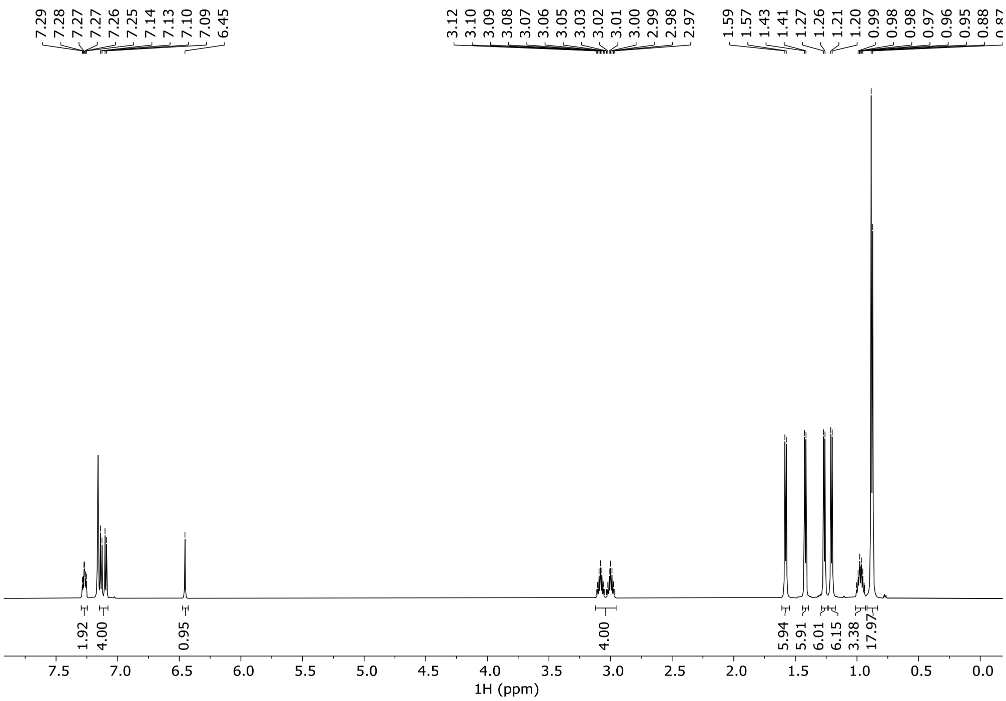

Figure S21. ^{1}H NMR (600 MHz, C_6D_6) spectrum of **6**.

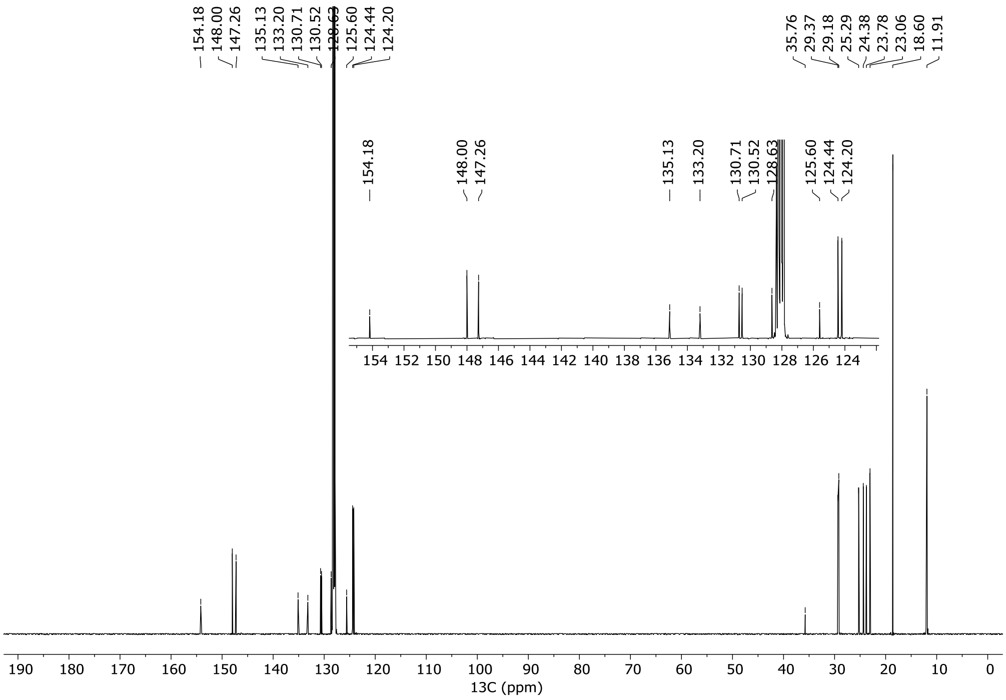

Figure S22. $\{^{1}H\}^{13}C$ NMR (151 MHz, C_6D_6) spectrum of **6**.

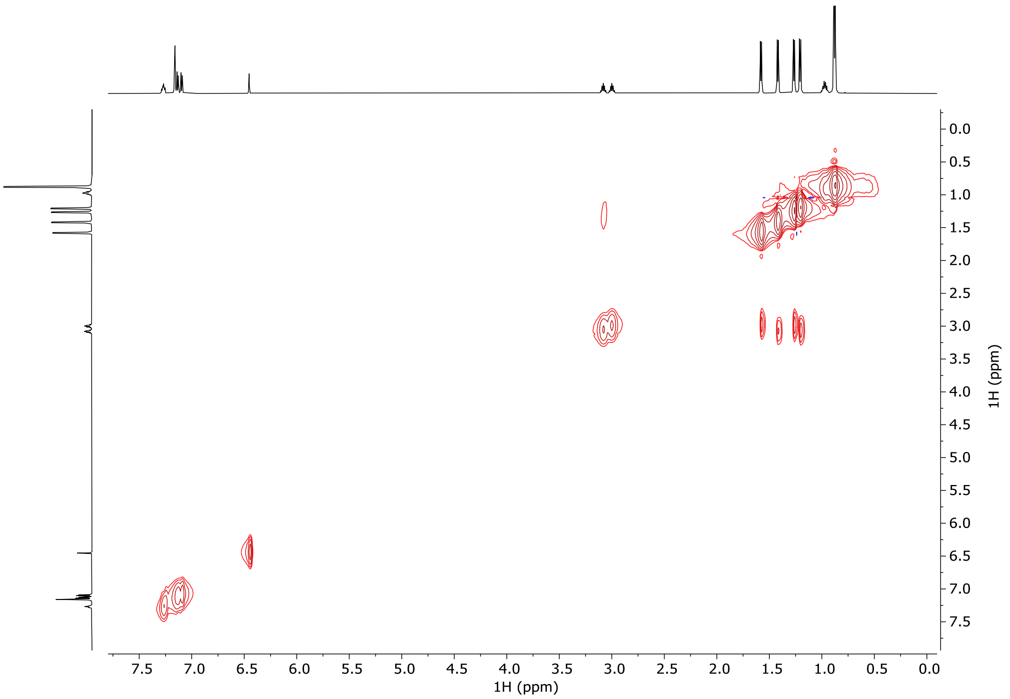

Figure S23. COSY NMR (C_6D_6) spectrum of **6**.

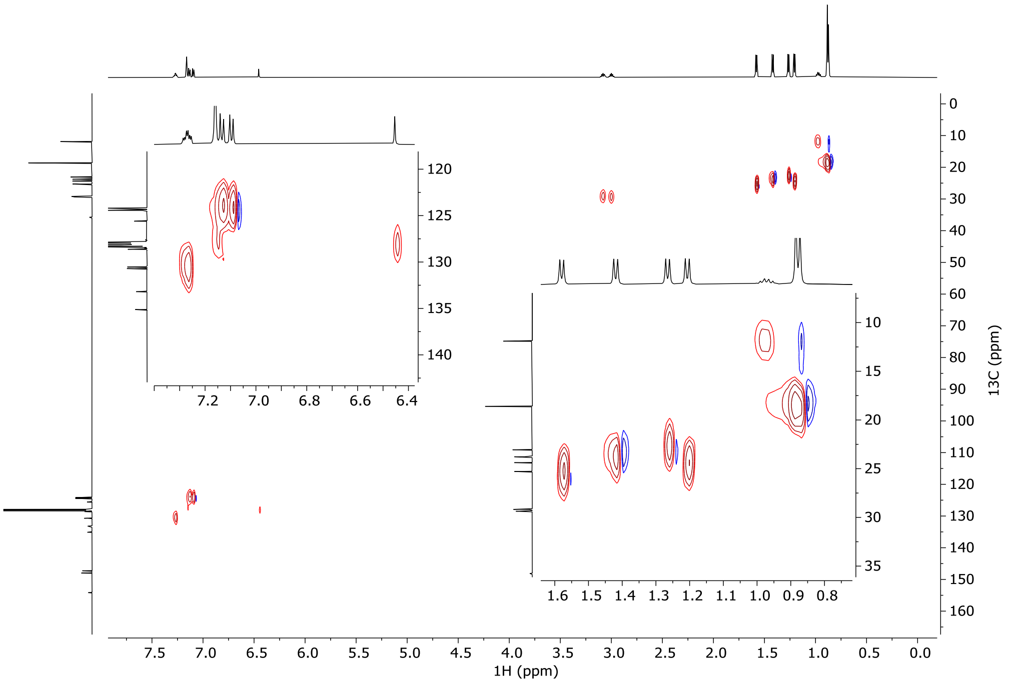

Figure S24. HSQC NMR (C_6D_6) spectrum of **6**.

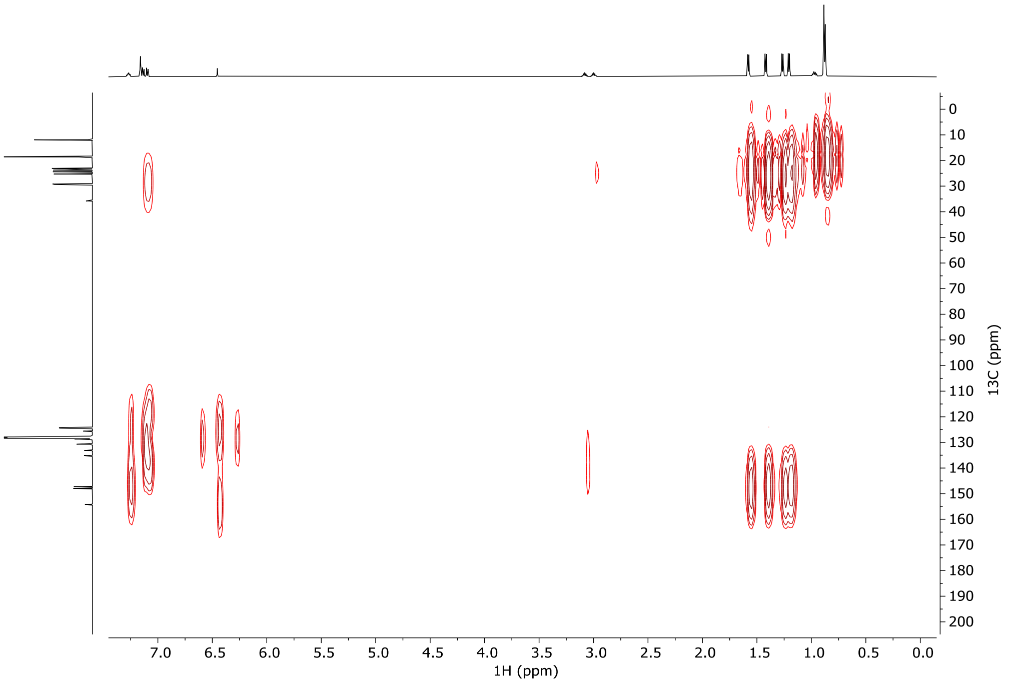

Figure S25. HMBC NMR (C_6D_6) spectrum of **6**.

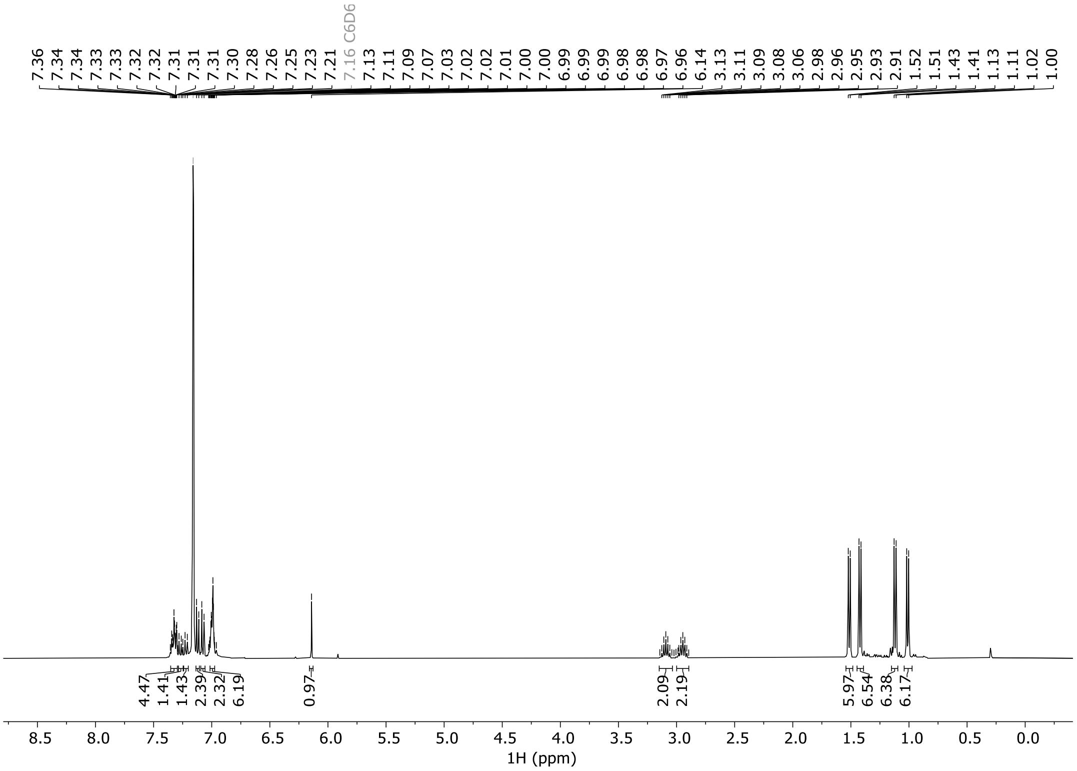

Figure **S26**. ${}^{1}H$ NMR (C_6D_6) spectrum of **7**.

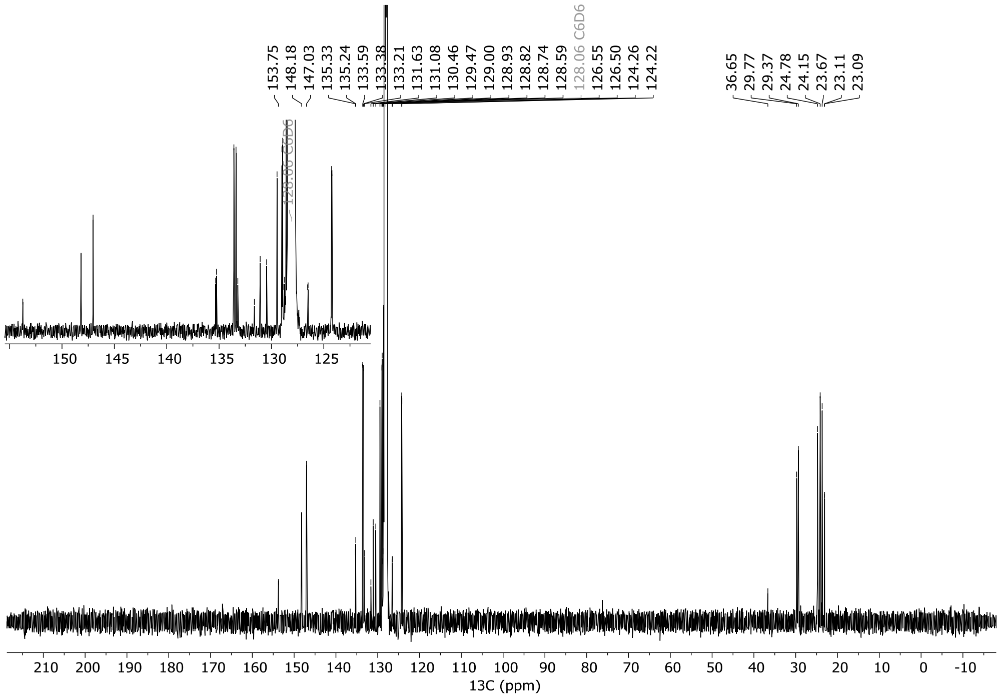

Figure S27. {¹H}^{13}C NMR (C_6D_6) spectrum of **7**.

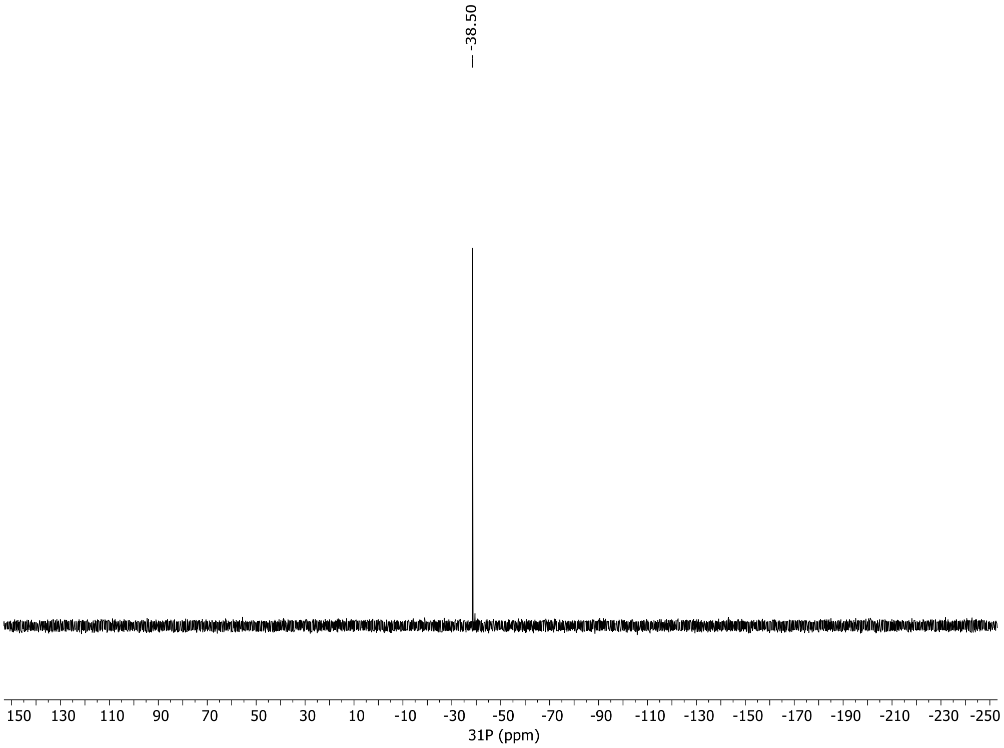

Figure S28. {¹H}^{31}P NMR (C_6D_6) spectrum of **7**.

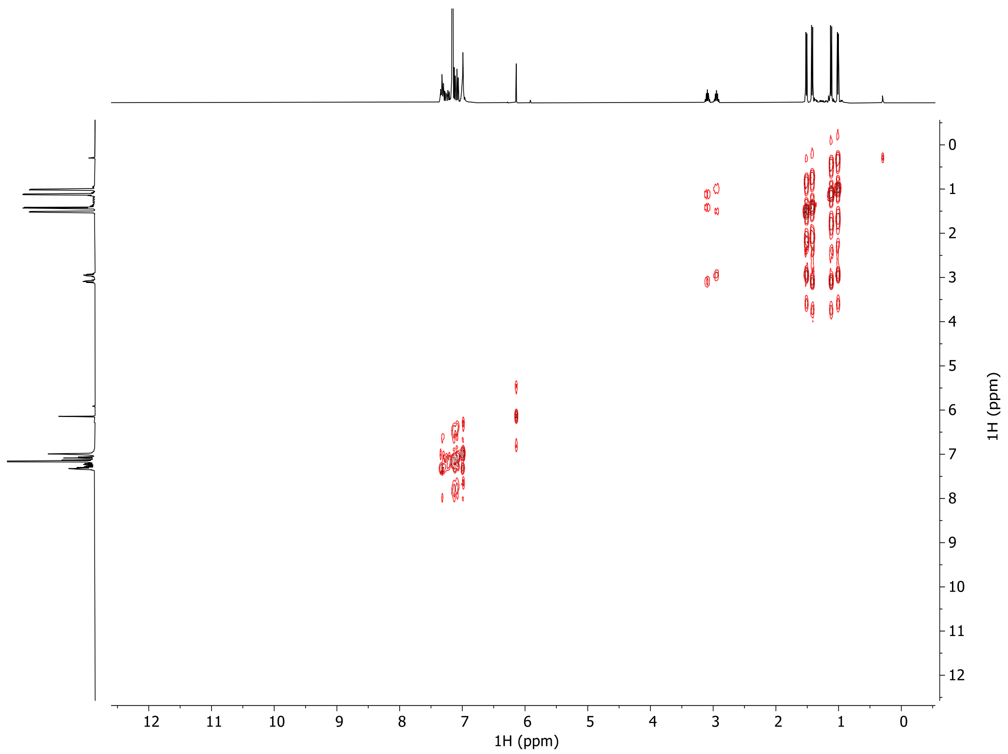

Figure **S29**. COSY NMR (C_6D_6) spectrum of **7**.

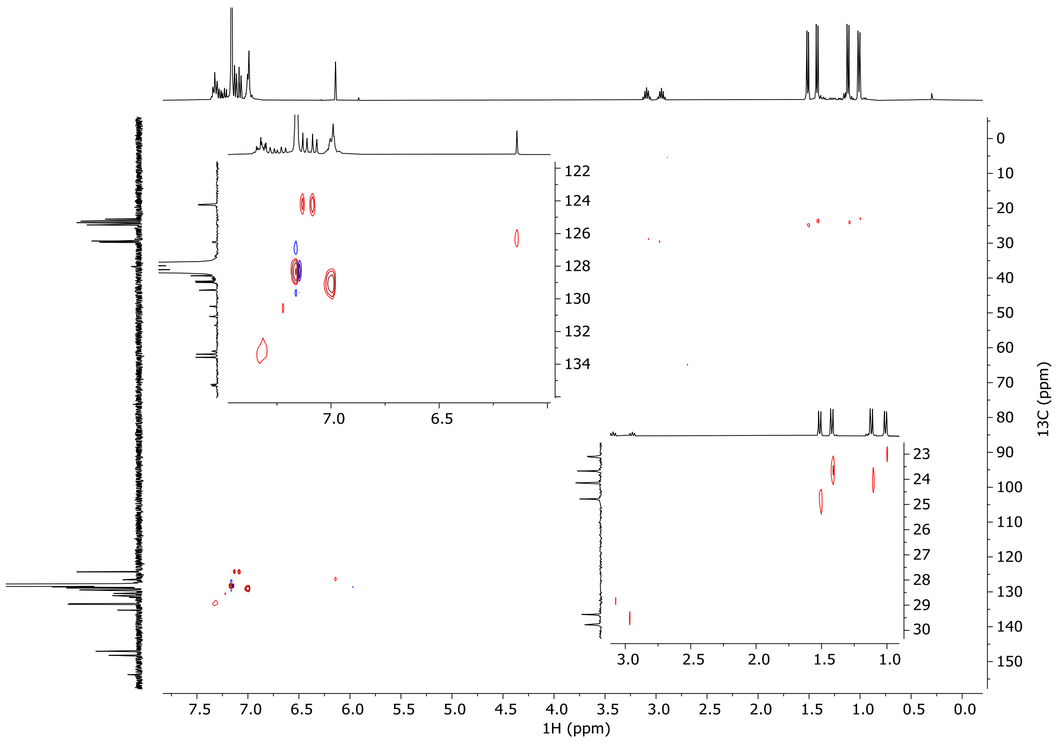

Figure **S30**. HSQC NMR (C_6D_6) spectrum of **7**.

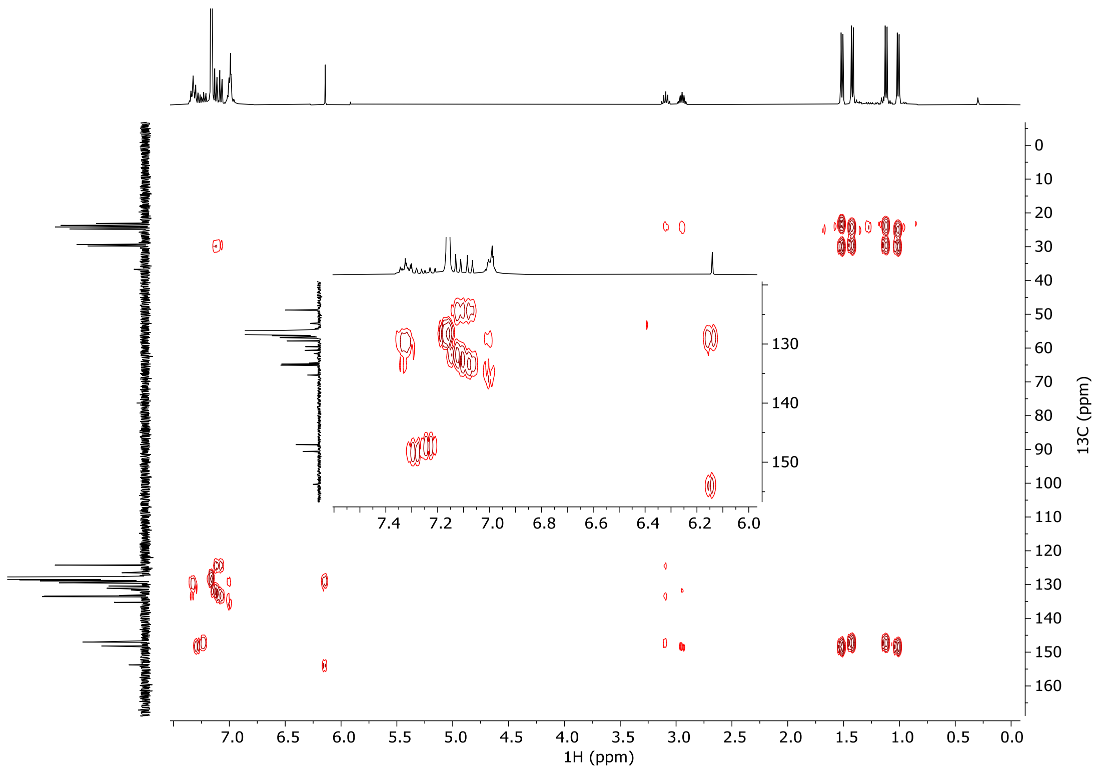

Figure **S31**. HMBC NMR (C_6D_6) spectrum of **7**.

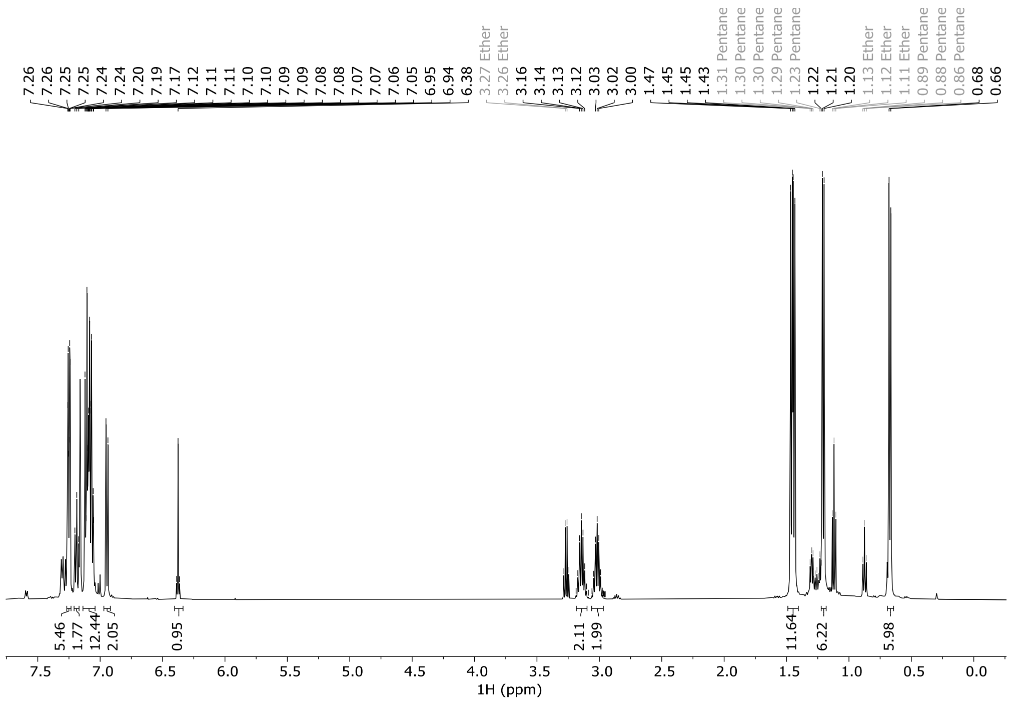

Figure S32. ^1H NMR (500 MHz, C_6D_6) spectrum of **8**.

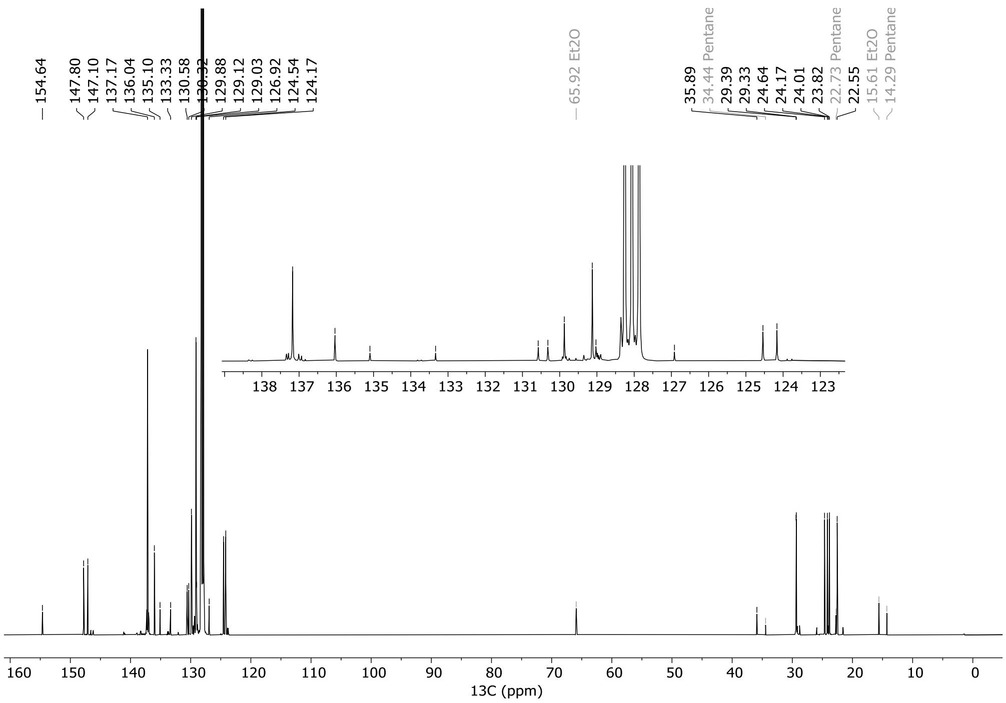

Figure S33. $\{^{1}H\}^{13}C$ NMR (126 MHz, C_6D_6) spectrum of **8**.

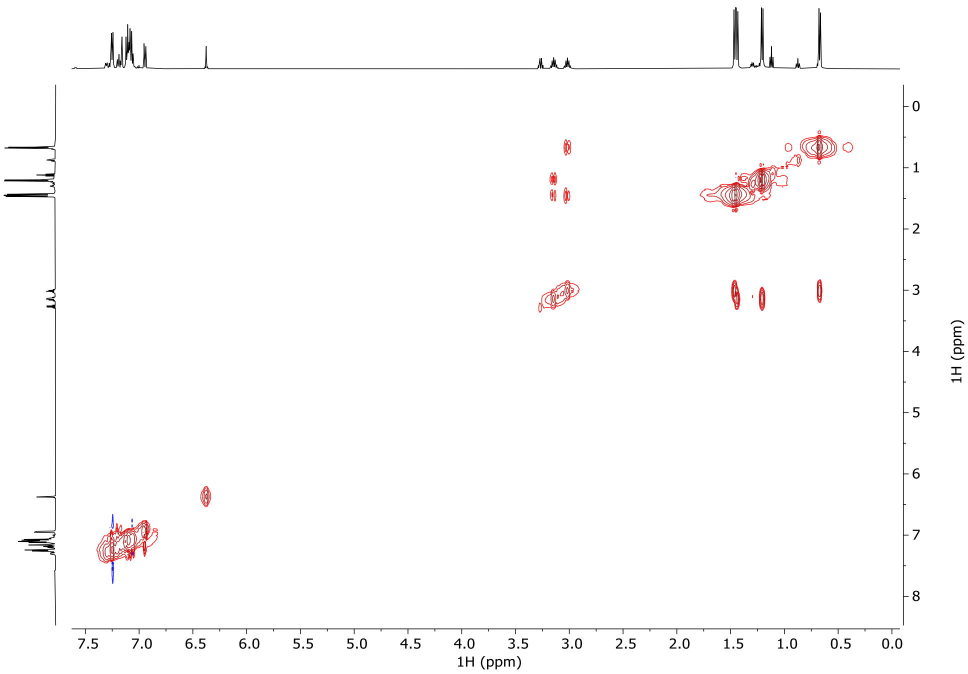

Figure S34. COSY NMR (C_6D_6) spectrum of **8**.

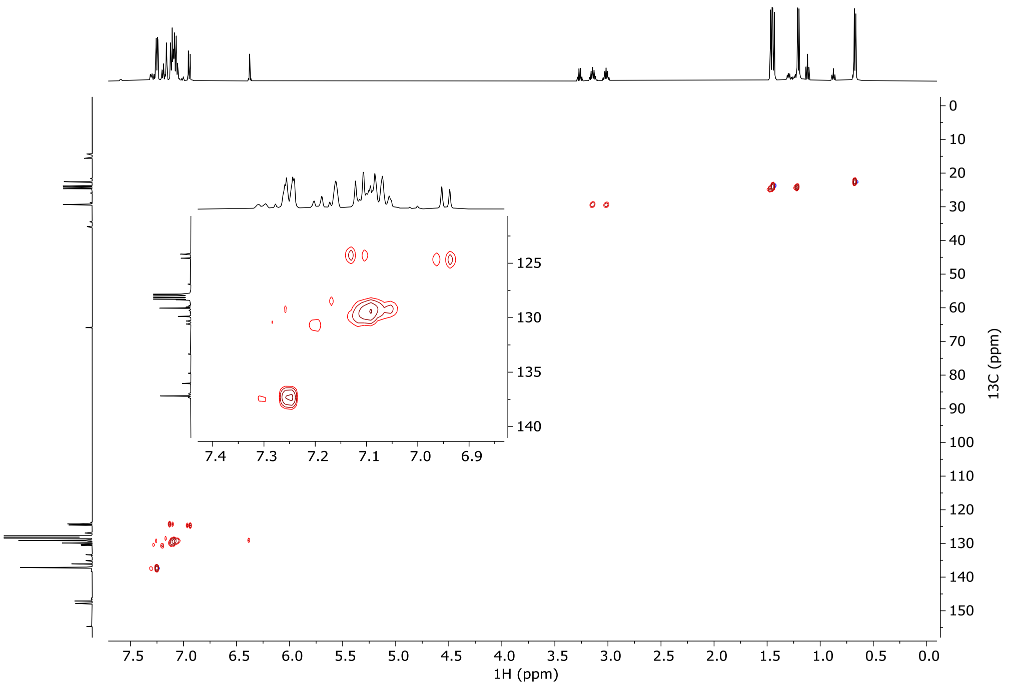

Figure S35. HSQC NMR (C_6D_6) spectrum of **8**.

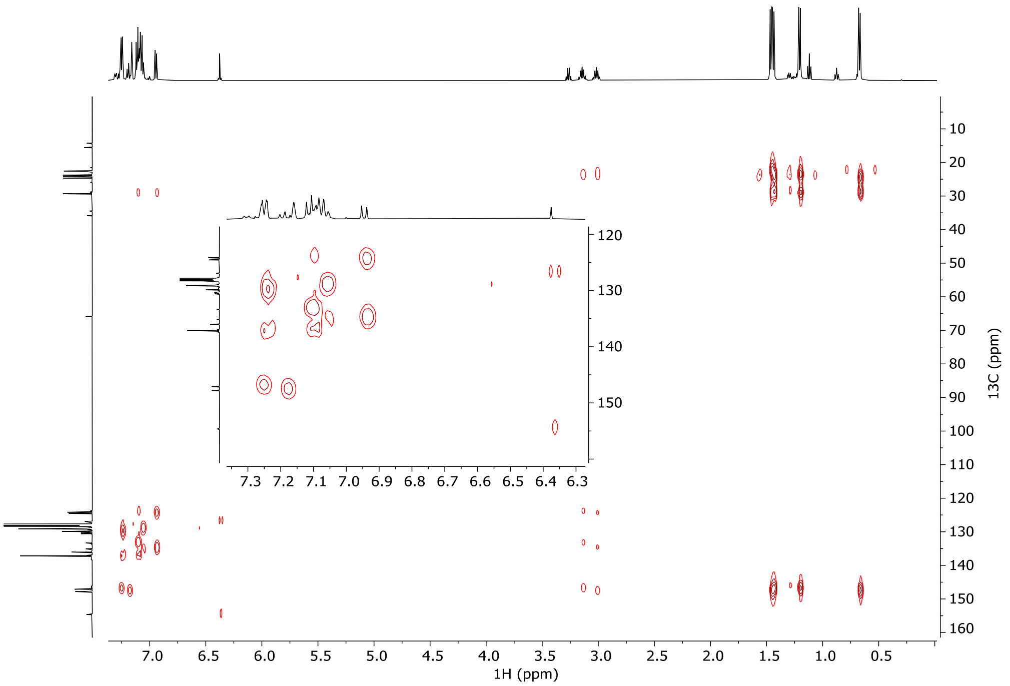

Figure S36. HMBC NMR (C_6D_6) spectrum of **8**.

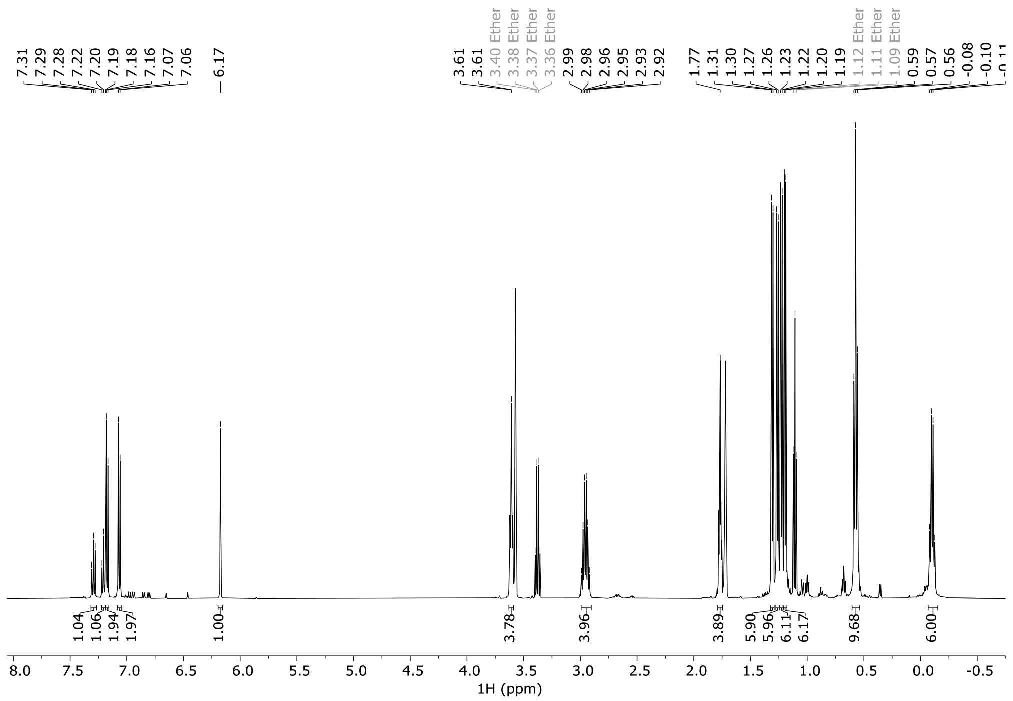

Figure S37. ^{1}H NMR (500 MHz, d_8-THF) spectrum of **9**.

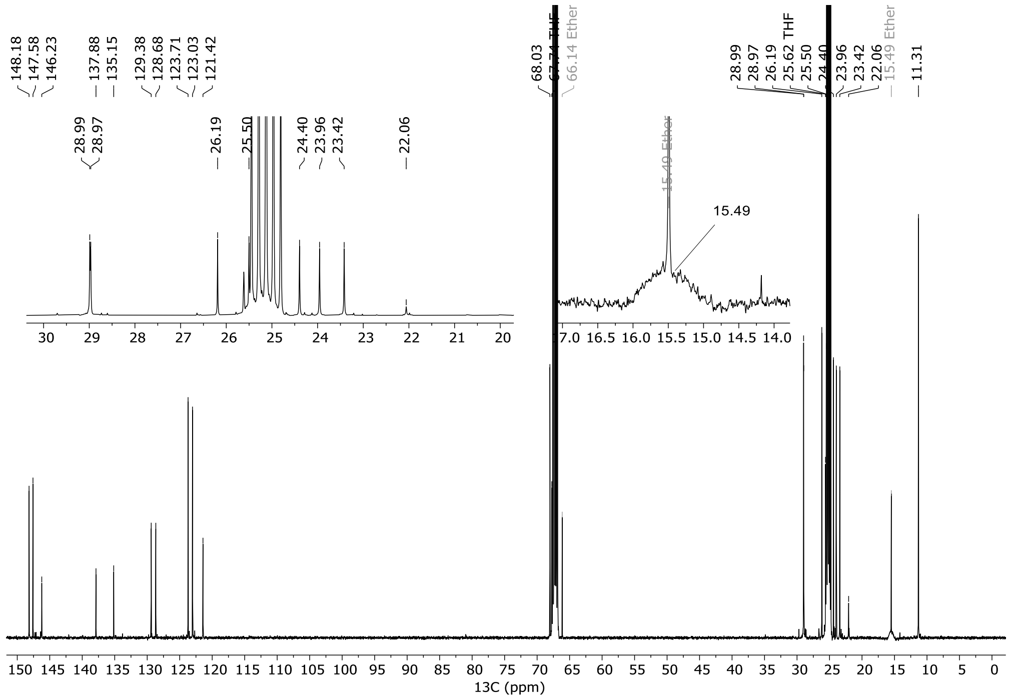

Figure S38. {^{1}H}^{13}C NMR (126 MHz, d_8-THF) spectrum of **9**.

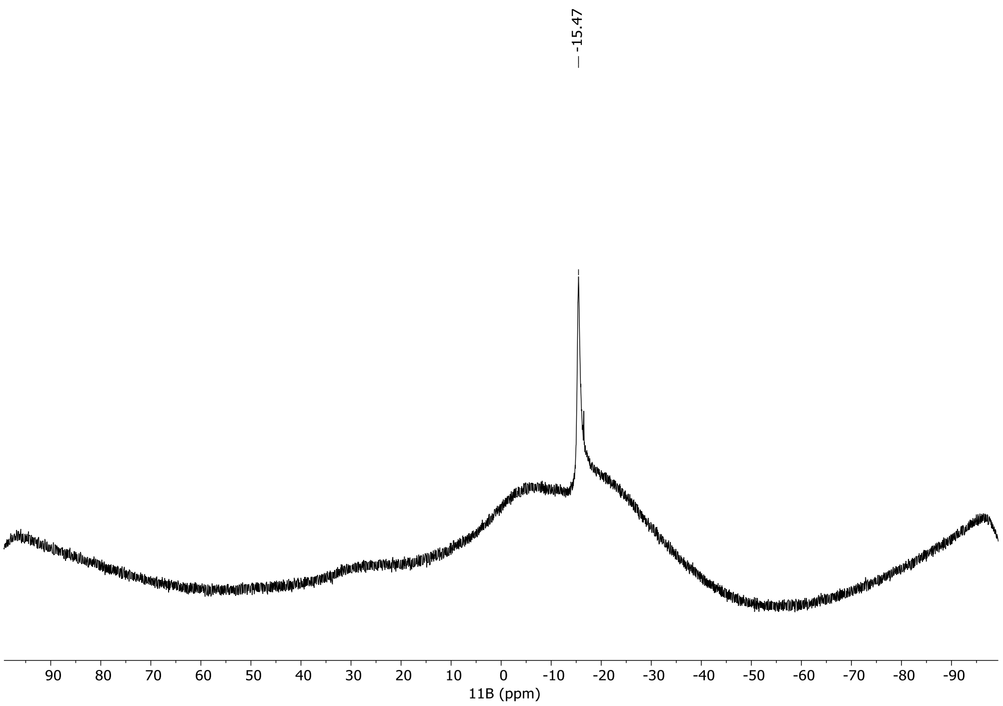

Figure S39. ^{11}B NMR (128 MHz, d_8-THF) spectrum of **9**.

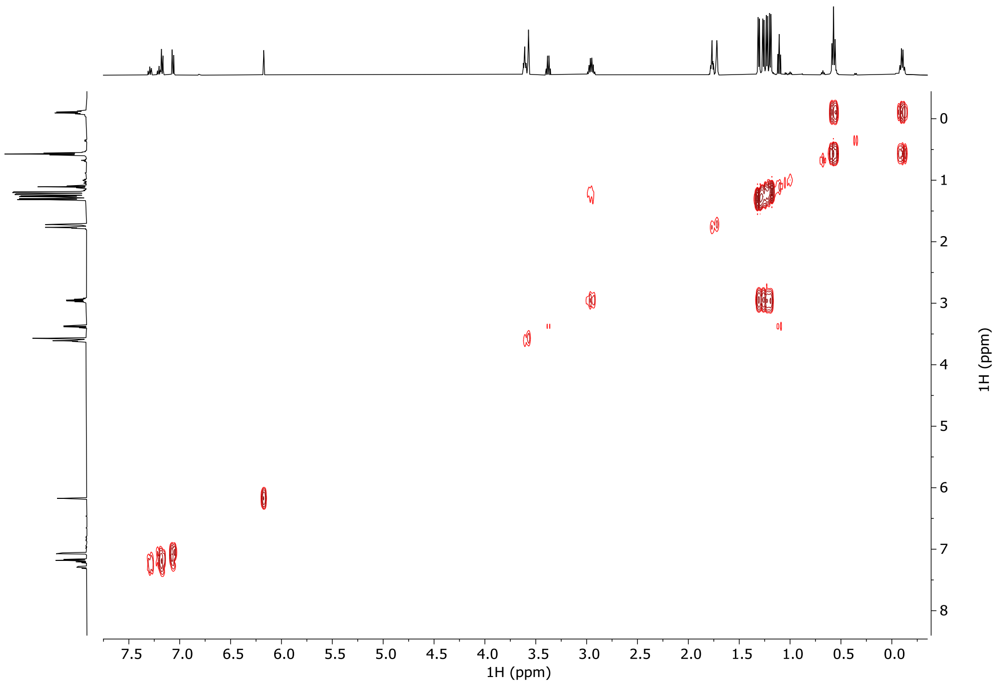

Figure S40. COSY NMR (d_8-THF) spectrum of **9**.

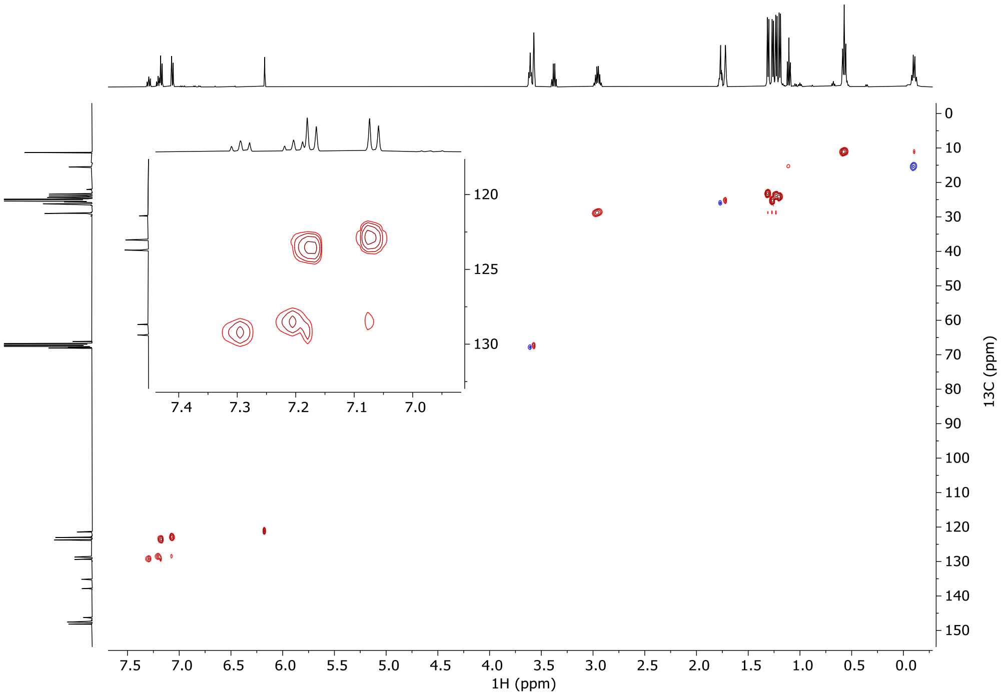

Figure S41. HSQC NMR (d_8-THF) spectrum of **9**.

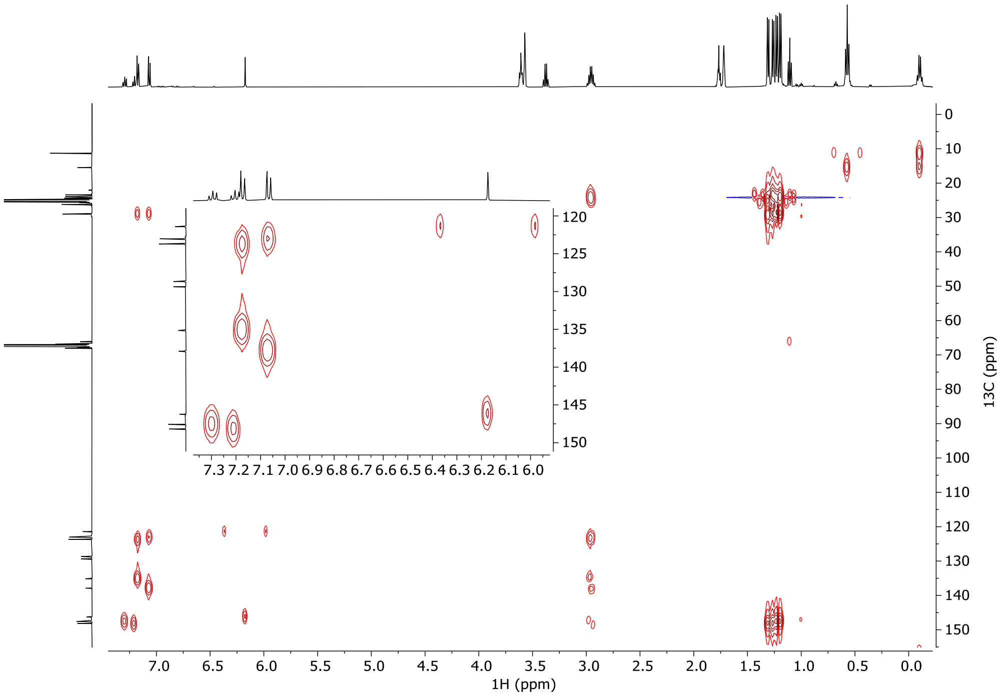

Figure S42. HMBC NMR (d_8-THF) spectrum of **9**.

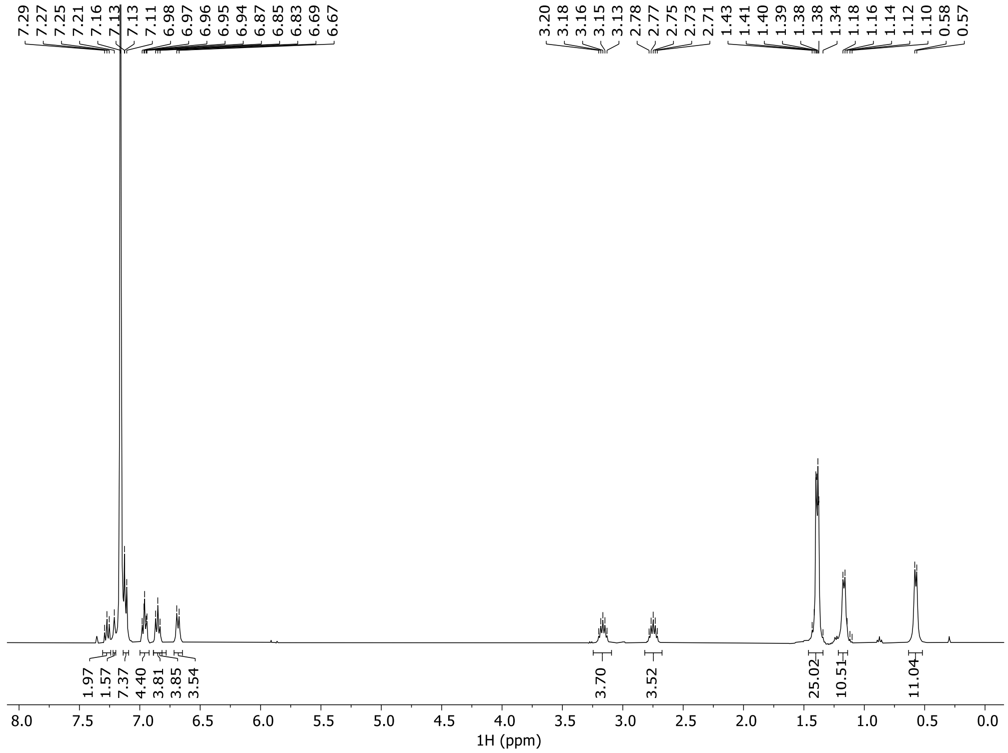

Figure S43. ^{1}H NMR (400 MHz, C_6D_6) spectrum of **10**.

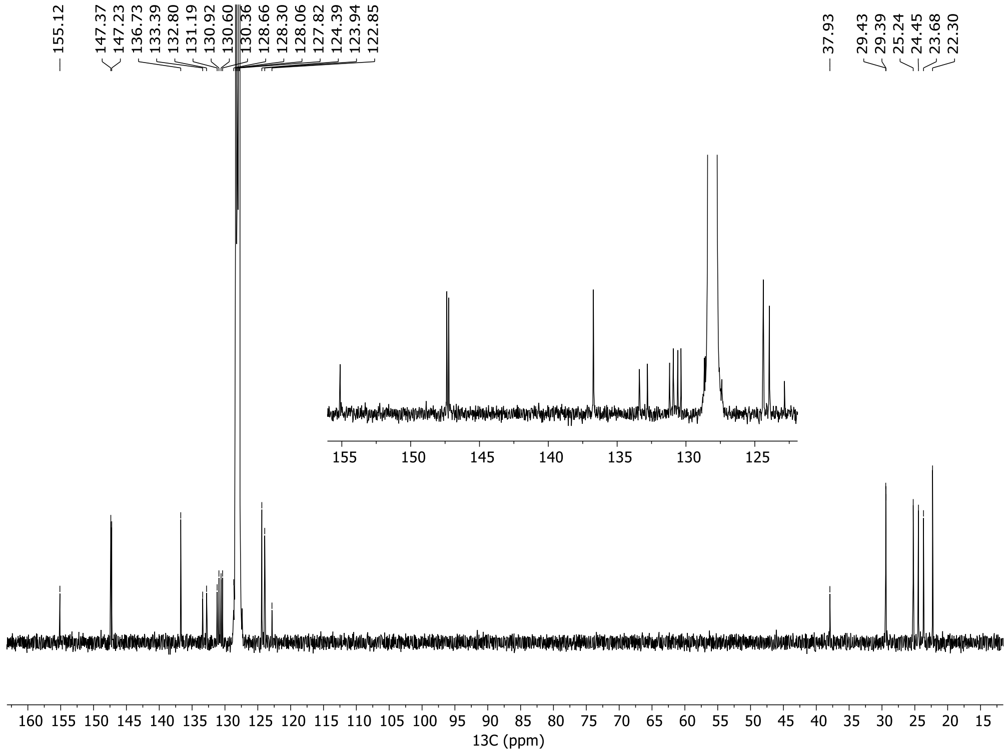

Figure S44. {^{1}H}^{13}C NMR (101 MHz, C_6D_6) spectrum of **10**.

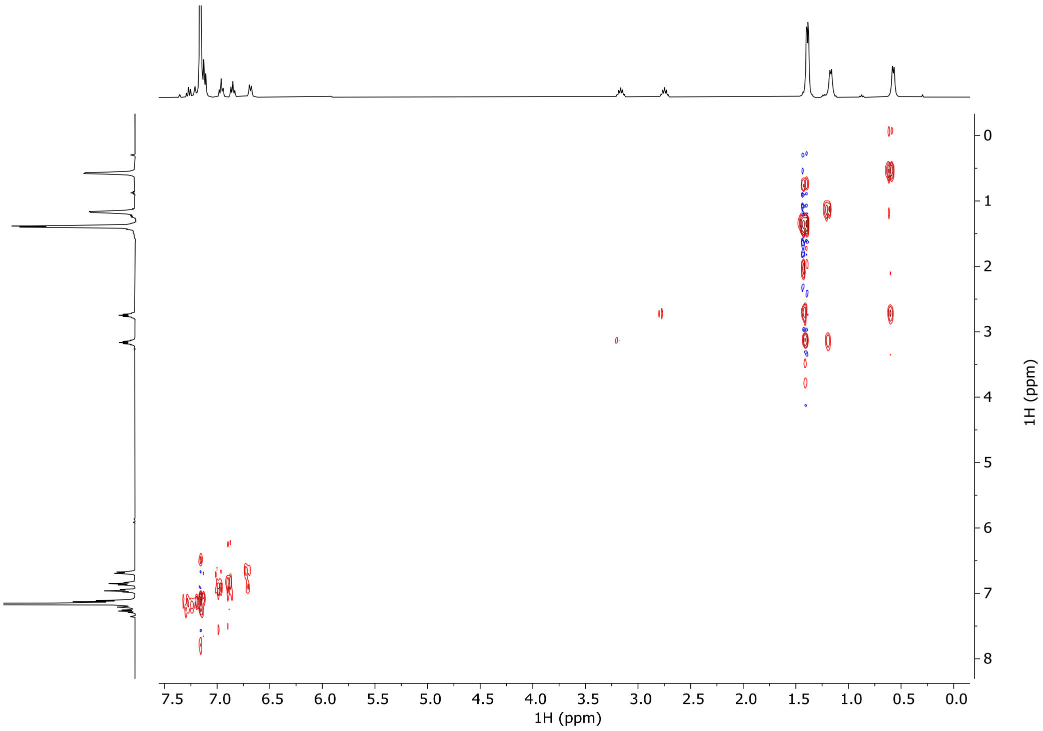

Figure S45. ^{1}H COSY NMR (C_6D_6) spectrum of **10**.

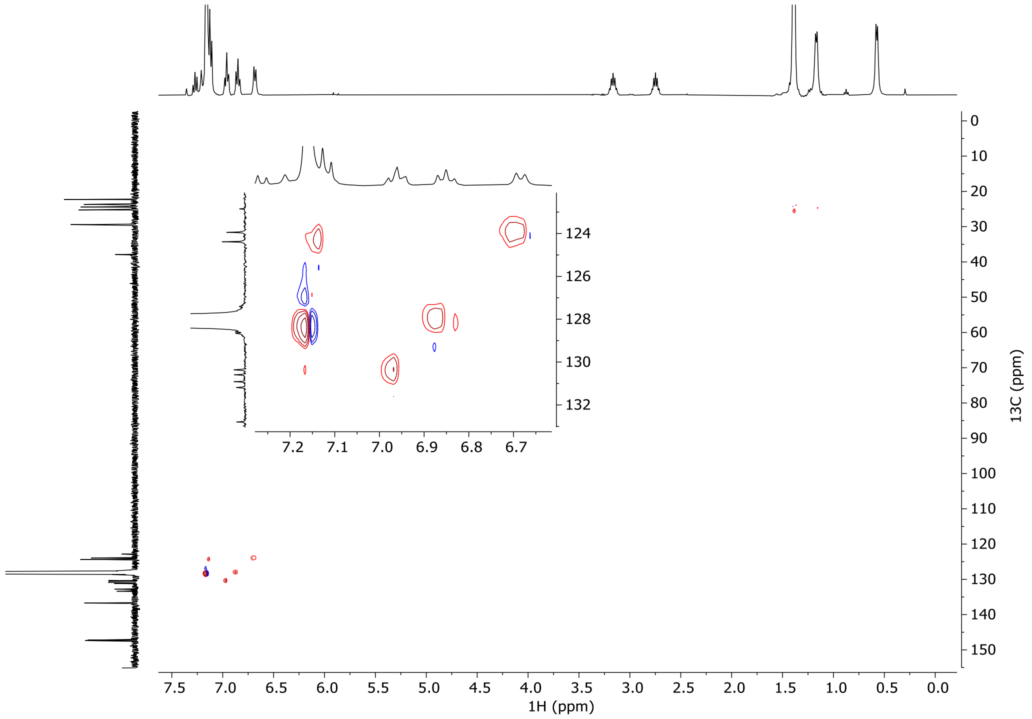

Figure S46. HSQC NMR (C_6D_6) spectrum of **10**.

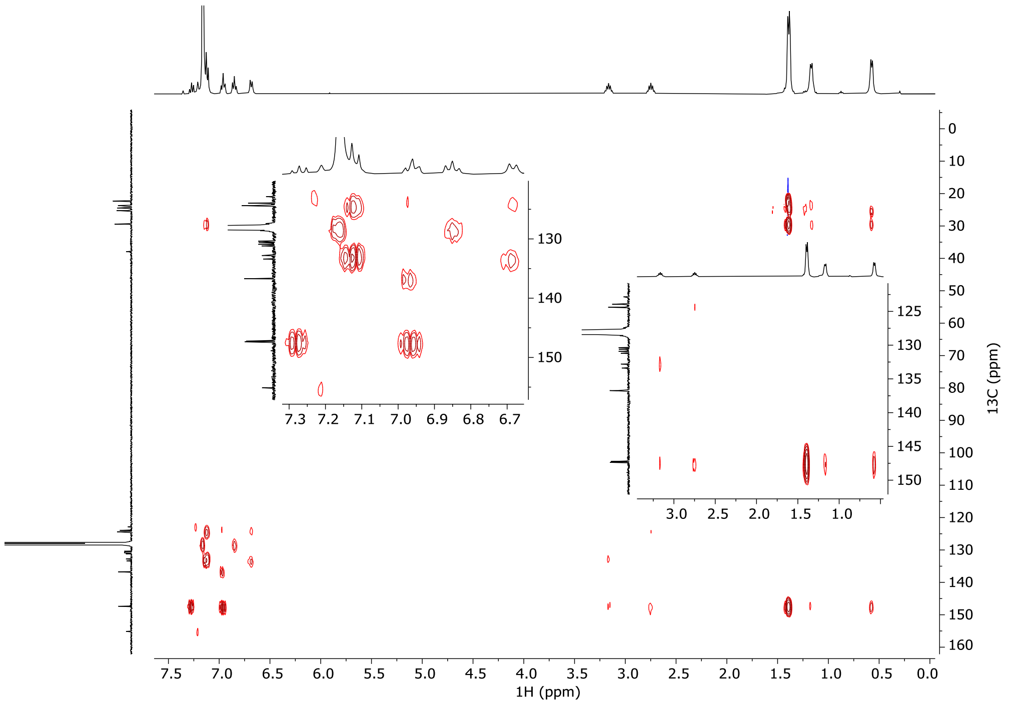

Figure S47. HMBC NMR (C_6D_6) spectrum of **10**.

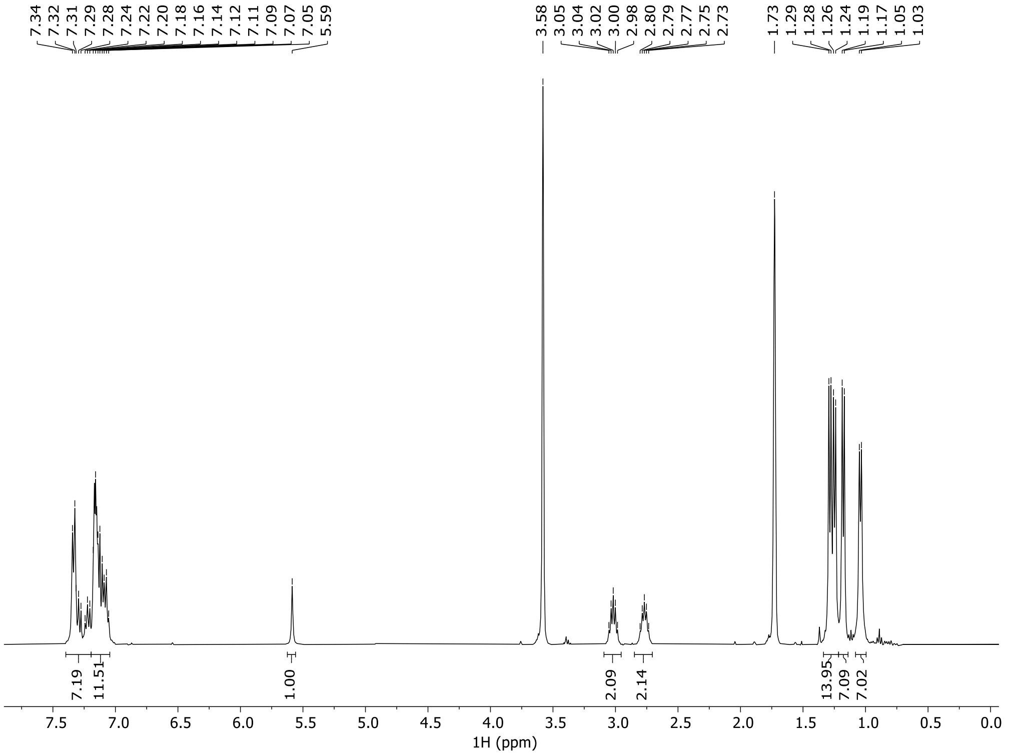

Figure S48. ^{1}H NMR (400 MHz, d_8-THF) spectrum of **11**.

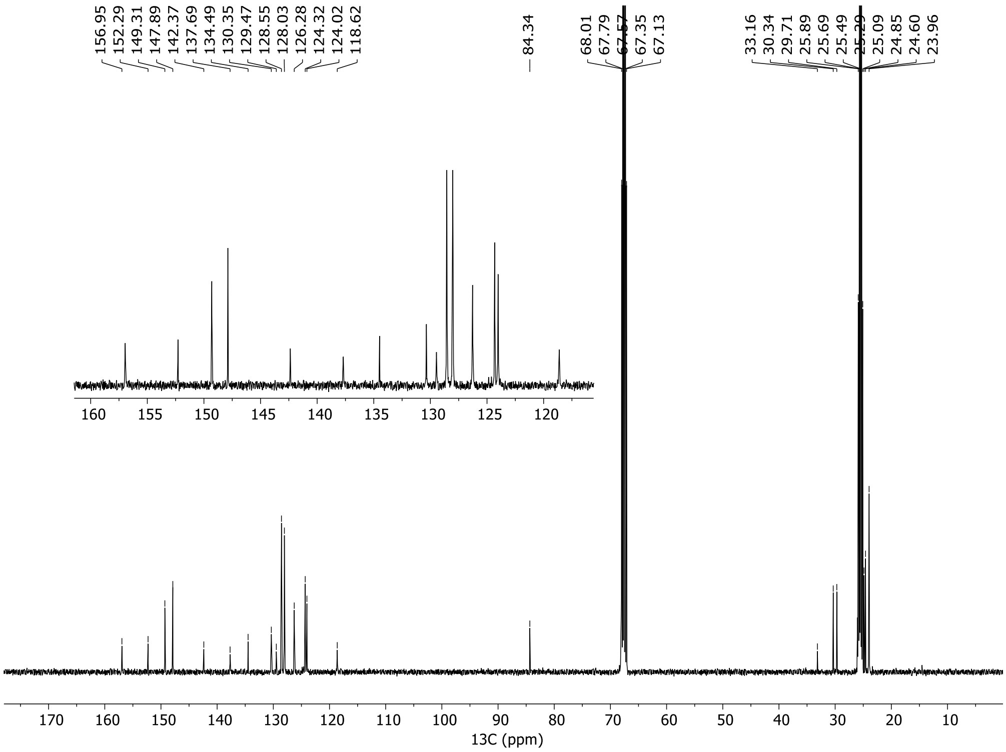

Figure S49. {^{1}H}^{13}C NMR (101 MHz, d_8-THF) spectrum of **11**.

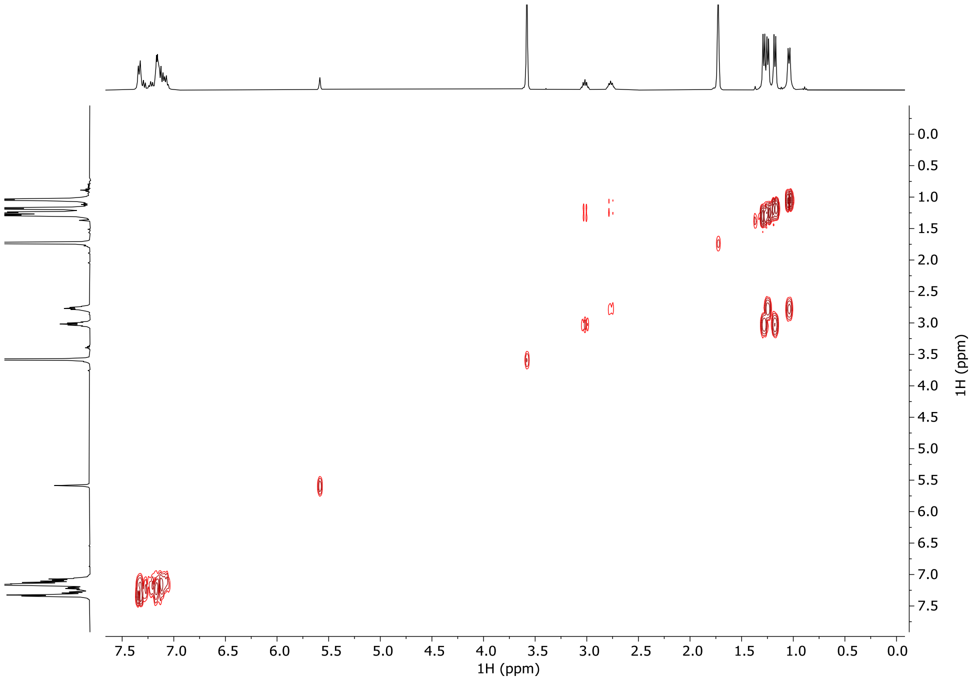

Figure S50. COSY NMR (d_8-THF) spectrum of **11**.

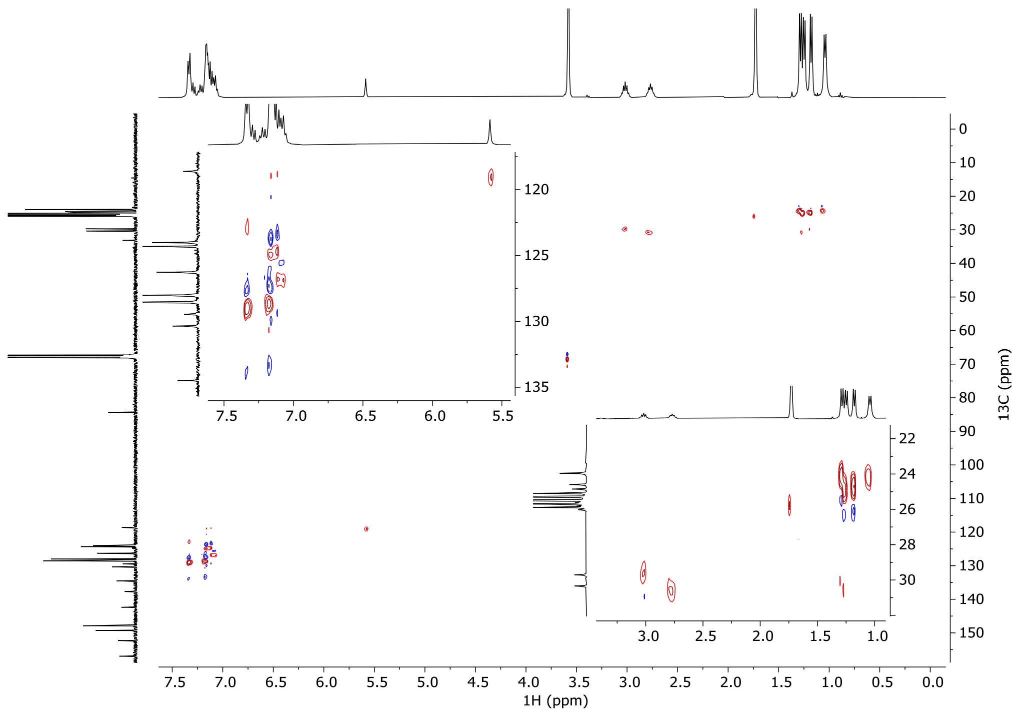

Figure S51. HSQC NMR (d_8-THF) spectrum of **11**.

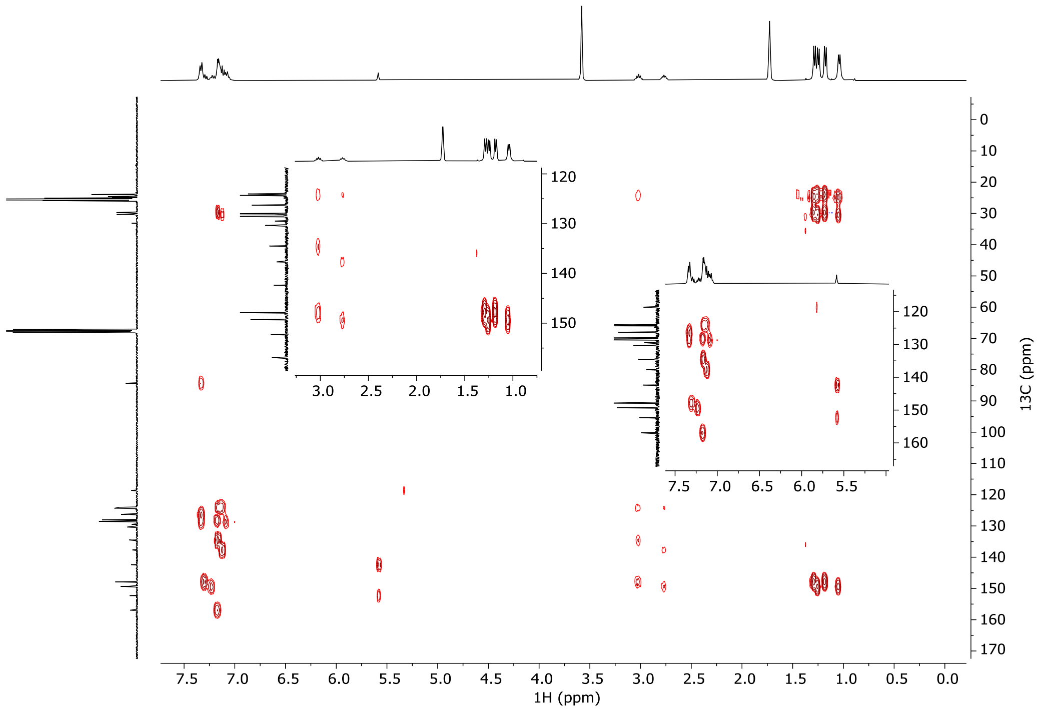

Figure S52. HMBC NMR (d_8-THF) spectrum of **11**.

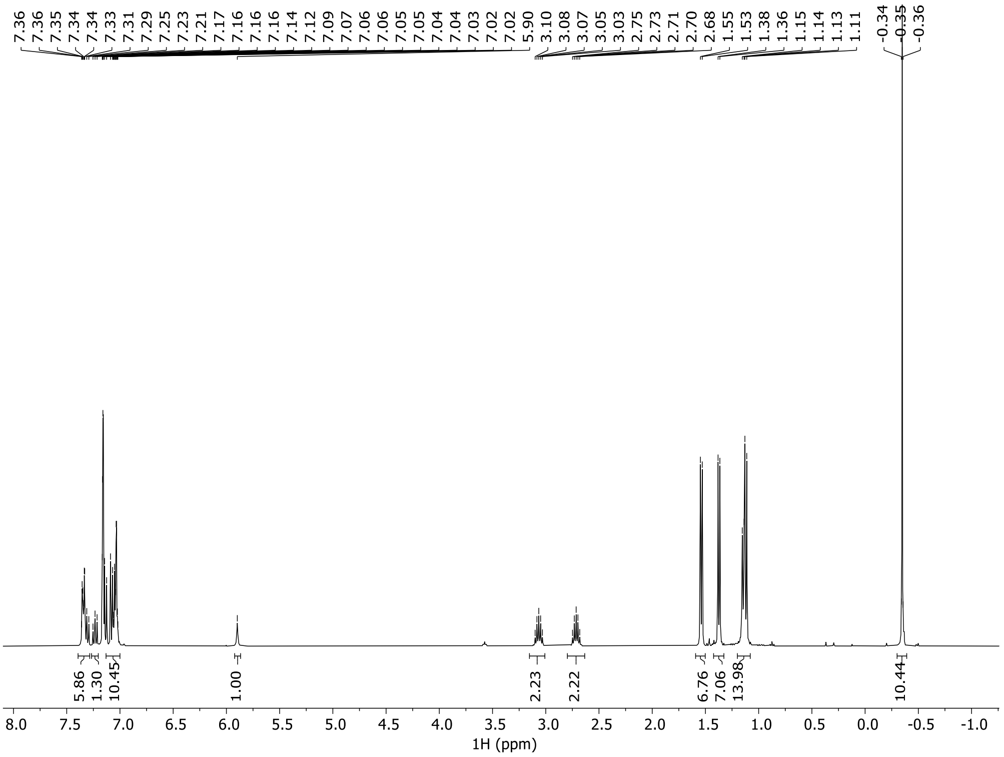

Figure S53. ^{1}H NMR (400 MHz, C_6D_6) spectrum of **12**.

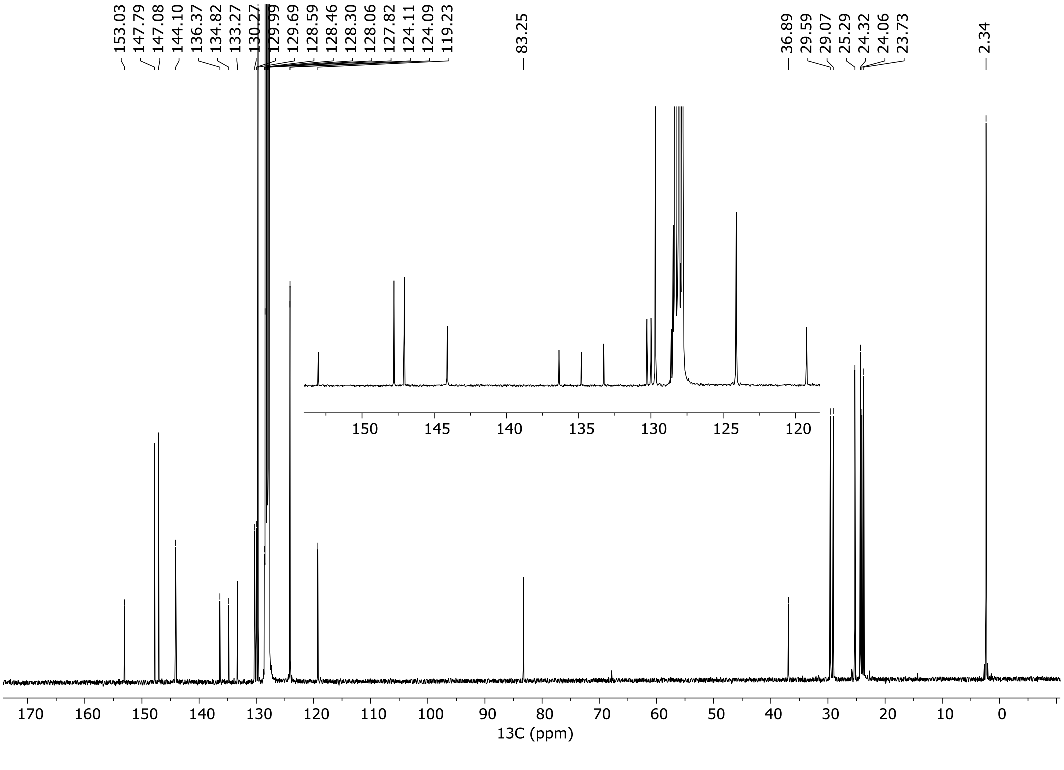

Figure S54. $\{^{1}H\}^{13}C$ NMR (101 MHz, C_6D_6) spectrum of **12**.

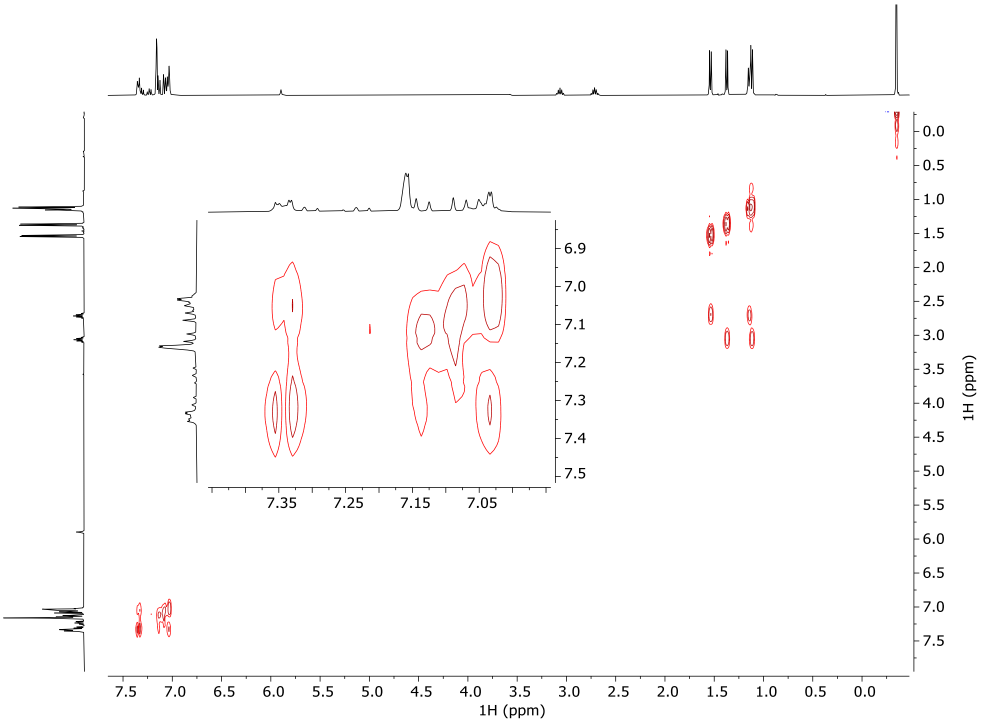

Figure S55. COSY NMR (C_6D_6) spectrum of **12**.

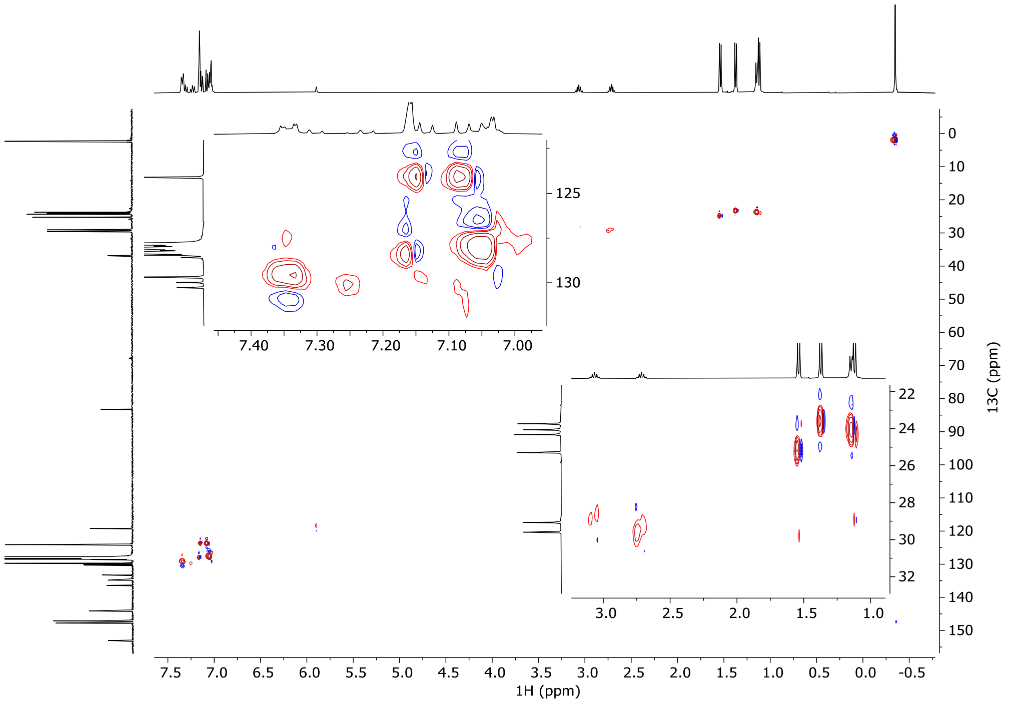

Figure S56. HSQC NMR (C_6D_6) spectrum of **12**.

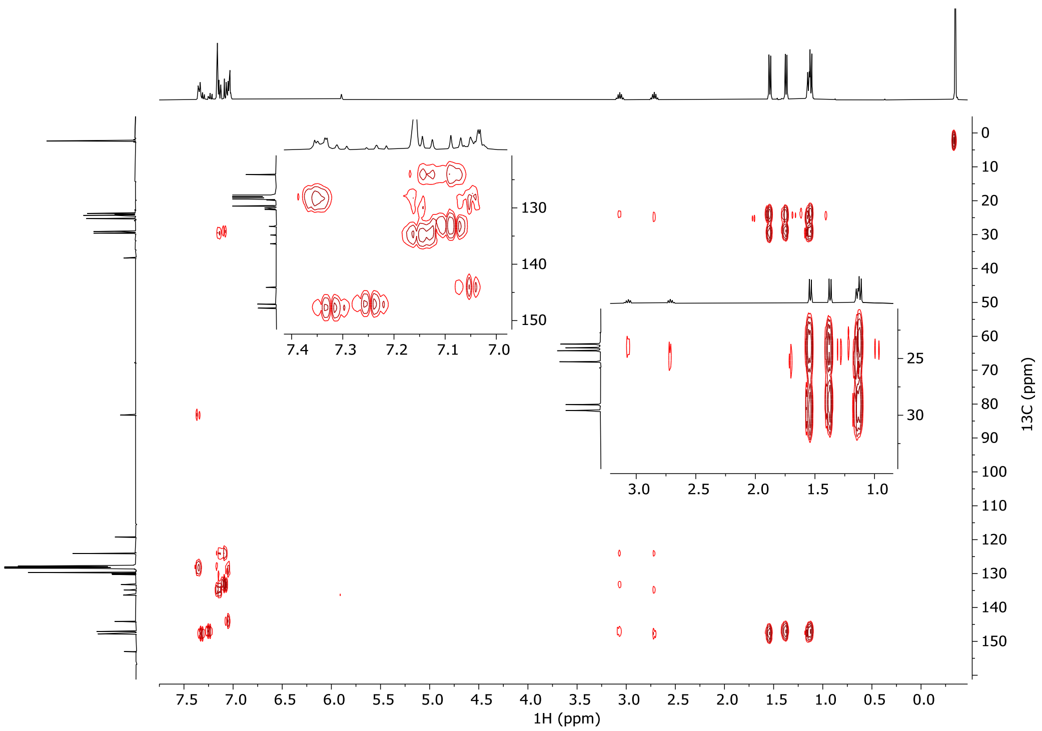

Figure S57. HMBC NMR (C_6D_6) spectrum of **12**.

4. ATR-IR Data

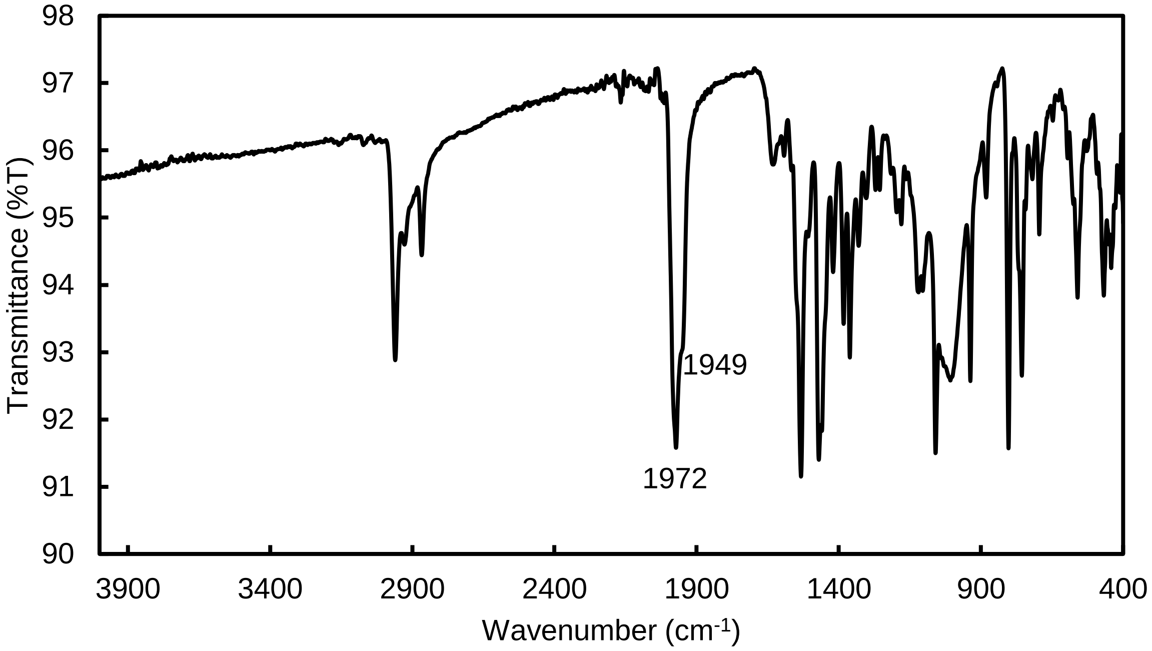

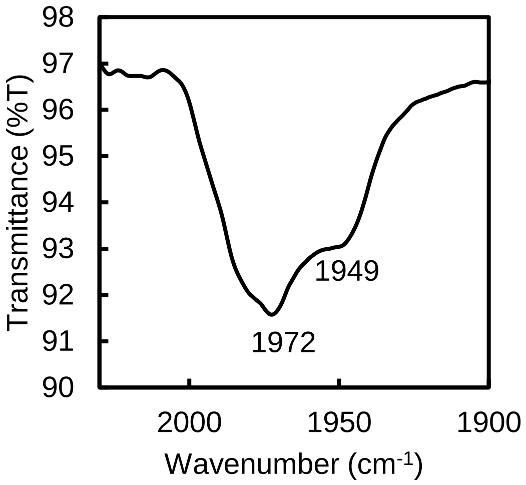

Figure **S58**. Solid-state ATR-IR spectrum of compound **2** (top) and an expanded view of the $\nu(N_2)$ band (bottom).

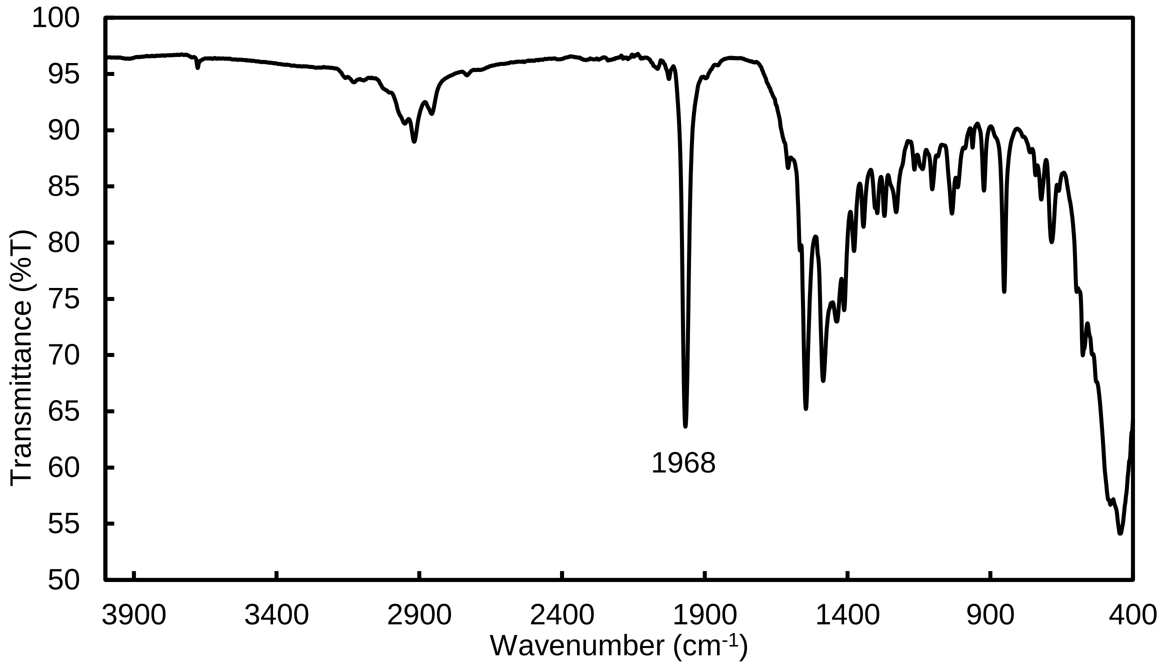

Figure **S59**. Solid-state ATR-IR spectrum of compound **3**.

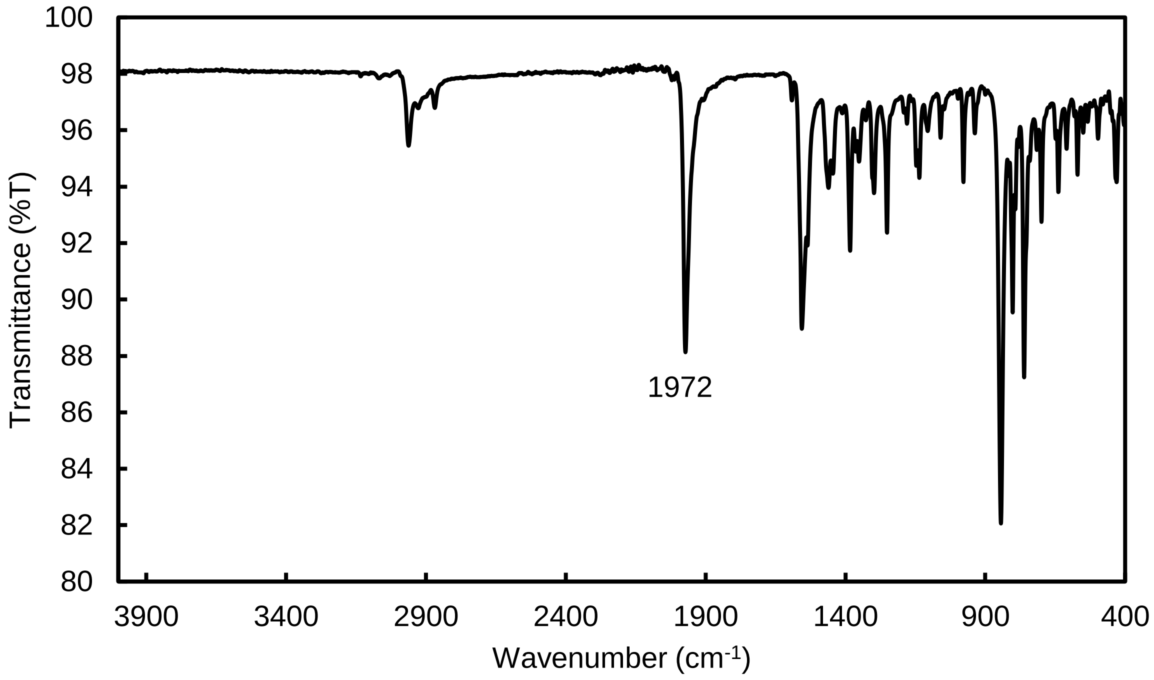

Figure **S60**. Solid-state ATR-IR spectrum of compound **4**.

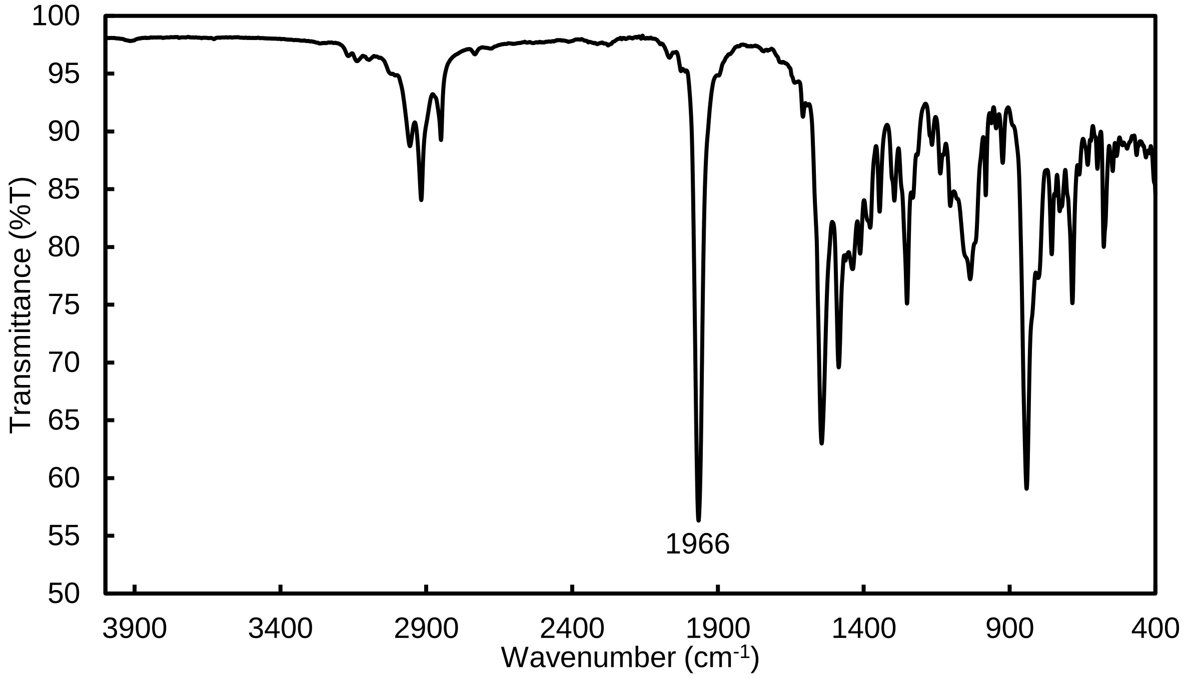

Figure **S61**. Solid-state ATR-IR spectrum of compound **5**.

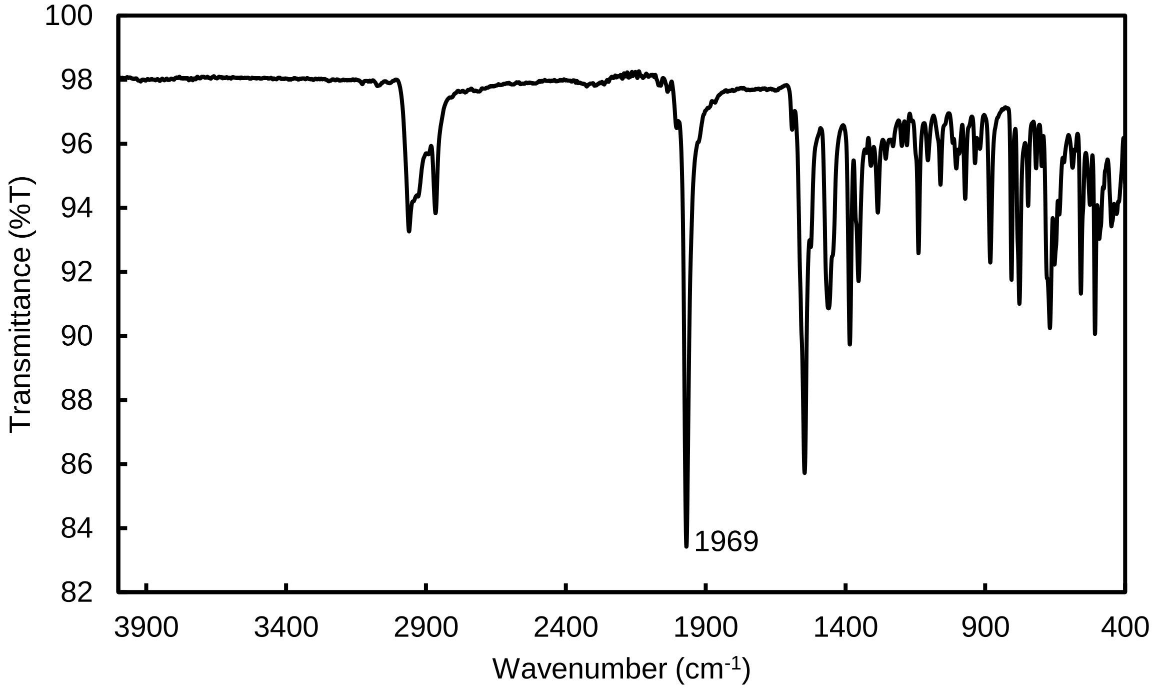

Figure **S62**. Solid-state ATR-IR spectrum of compound **6**.

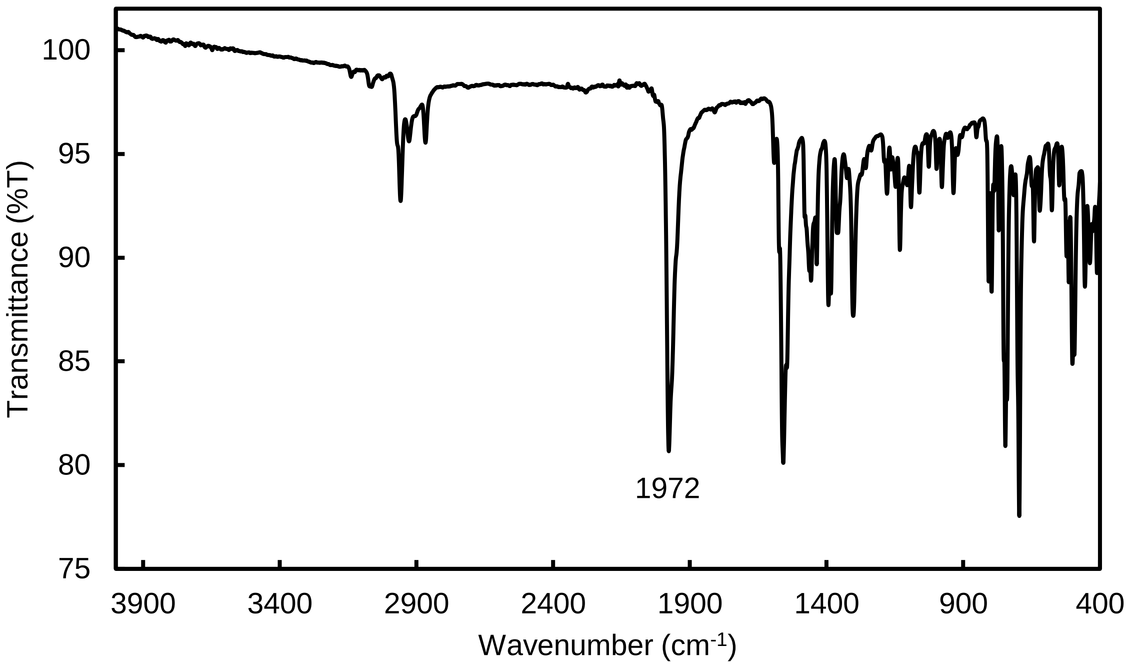

Figure **S63**. Solid-state ATR-IR spectrum of compound **7**.

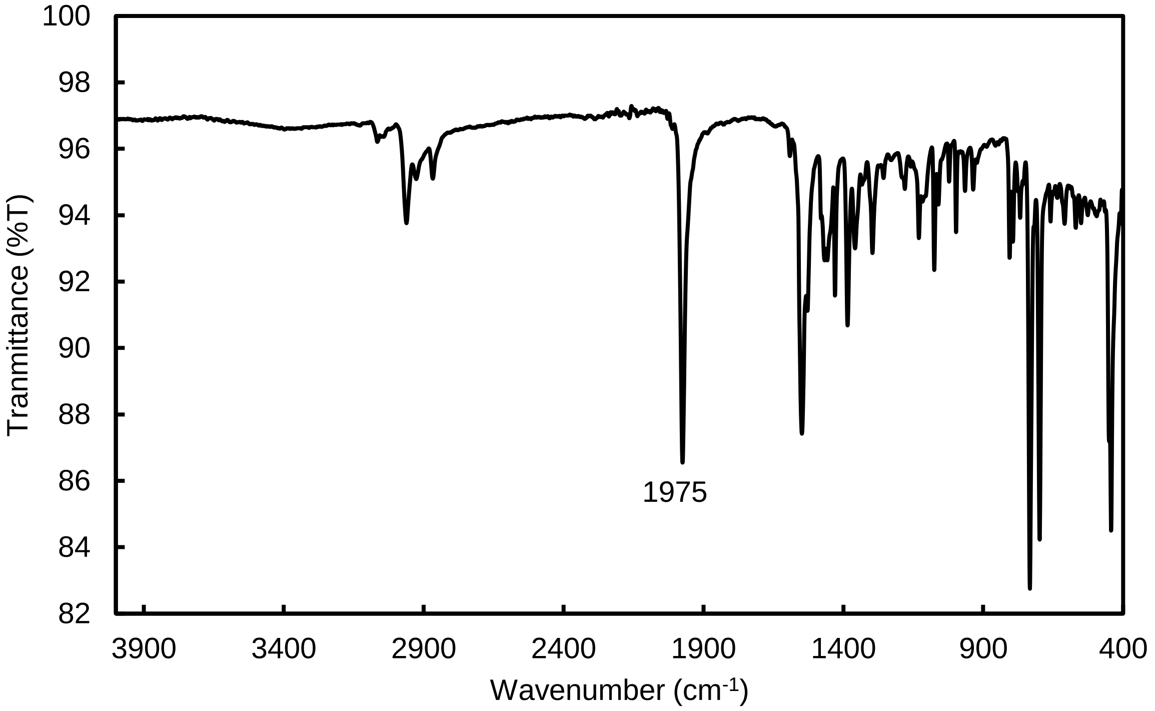

Figure **S64**. Solid-state ATR-IR spectrum of compound **8**.

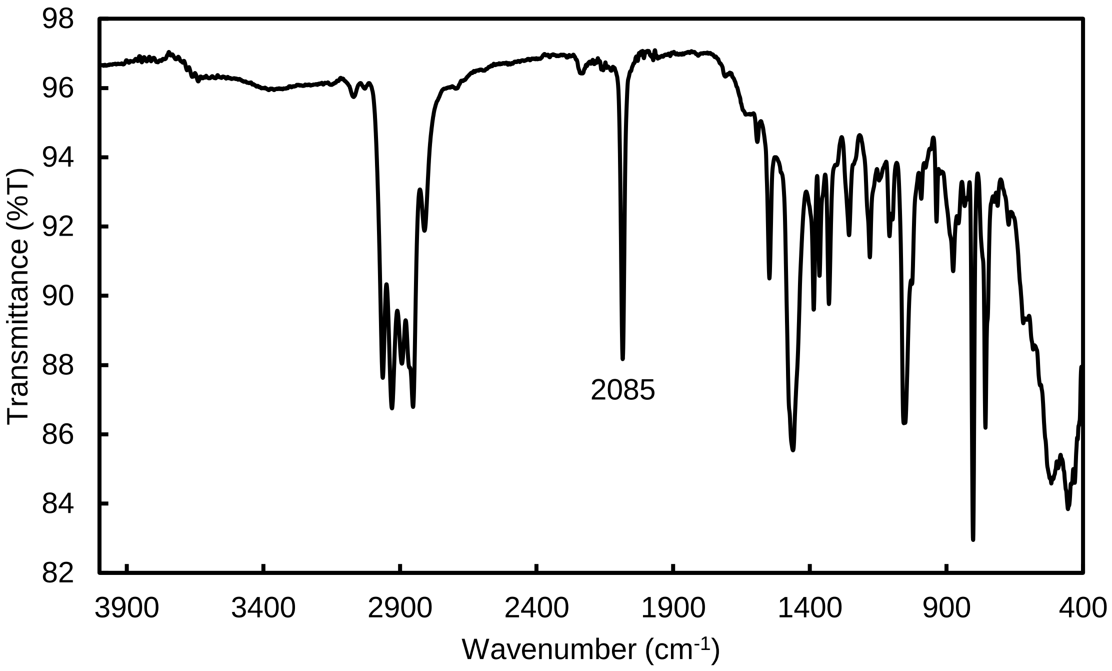

Figure **S65**. Solid-state ATR-IR spectrum of compound **9**.

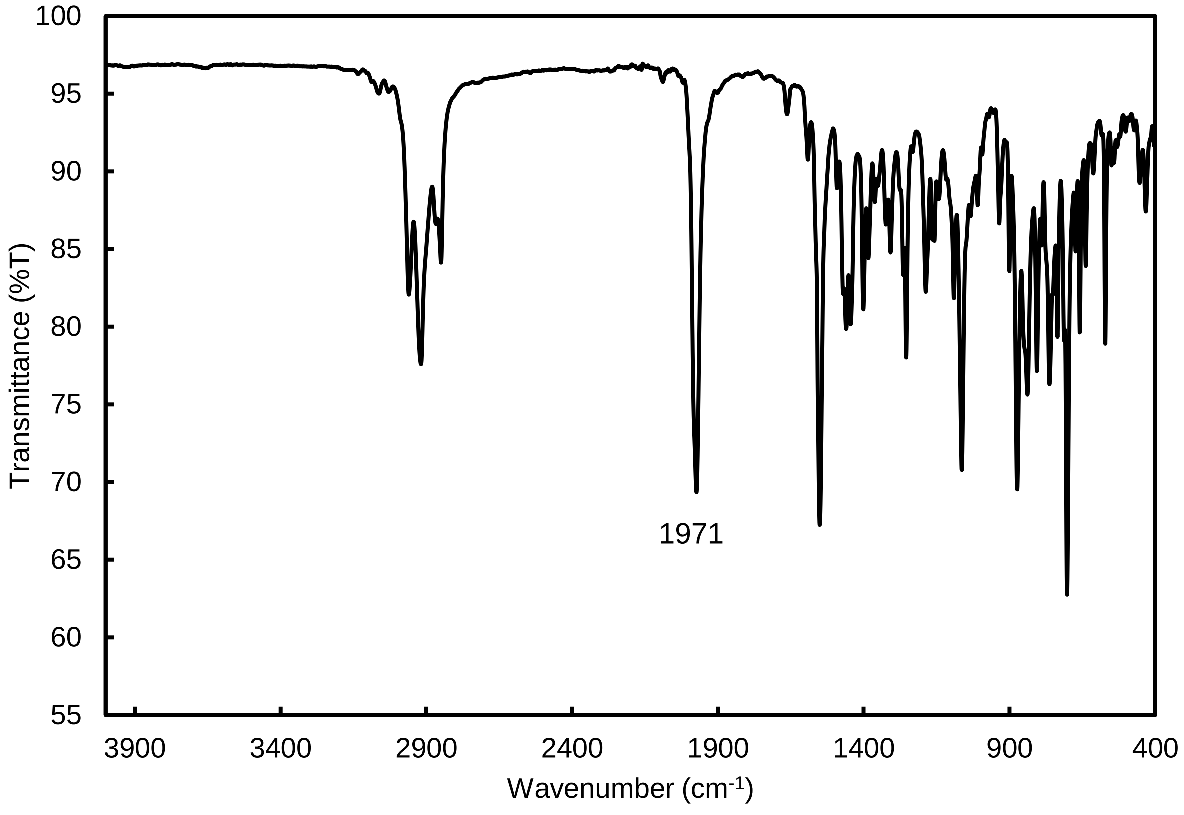

Figure **S66**. Solid-state ATR-IR spectrum of compound **10**.

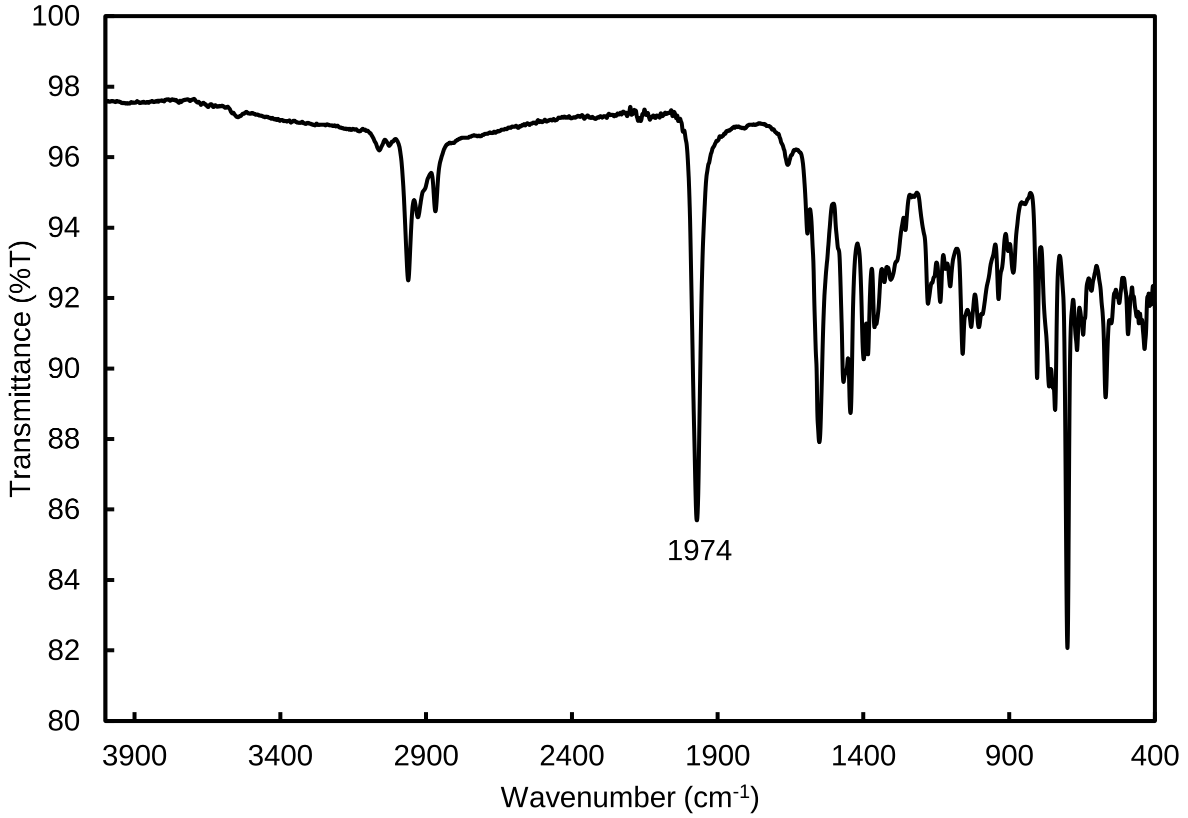

Figure **S67**. Solid-state ATR-IR spectrum of compound **11**.

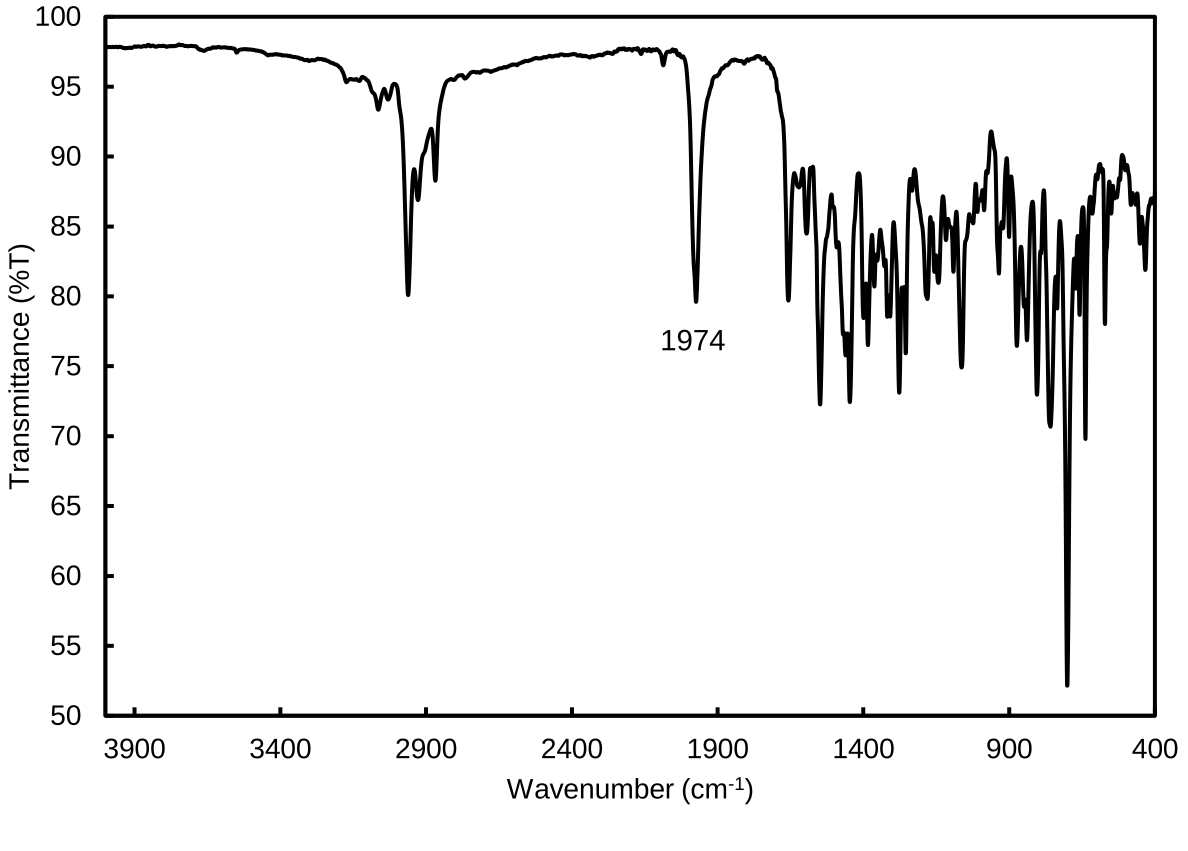

Figure **S68**. Solid-state ATR-IR spectrum of compound **12**.

5. XRD Analyses

Compound 2

Structure Quality Indicators

**Reflections:**	d min (CuKα) 2Θ=145.6° 0.81	I/σ(I) 46.1	Rint m=2.53 1.99%	Full 135.4° 97% to 145.6° 99.6
**Refinement:**	Shift 0.003	Max Peak 0.7	Min Peak -1.0	GooF 1.023

A clear intense yellow prism-shaped crystal with dimensions 0.27 × 0.20 × 0.18 mm was mounted. Data were collected using a SuperNova, Dual, Cu at home/near, AtlasS2 diffractometer operating at *T* = 139.99(10) K.

Data were measured using ω scans with Cu K_{α} radiation. The diffraction pattern was indexed, and the total number of runs and images was based on the strategy calculation from the program CrysAlis^{Pro} system (CCD 44.140a 64-bit (release 27-01-2026)).[4] The maximum resolution achieved was Θ = 72.716° (0.81 Å).

The unit cell was refined using CrysAlis^{Pro} on 13844 reflections, 38% of the observed reflections.

Data reduction, scaling and absorption corrections were performed using CrysAlis^{Pro}. The final completeness is 99.60 % out to 72.716° in Θ. An analytical absorption correction was performed using CrysAlis^{Pro} 1.171.44.142a. The analytical numeric absorption correction was done using a multifaceted crystal model based on expressions derived by R.C. Clark & J.S. Reid.[5] The empirical absorption correction was done using spherical harmonics, implemented in SCALE3 ABSPACK scaling algorithm. The absorption coefficient μ of this crystal is 1.506 mm^{-1} at this wavelength (λ = 1.54184Å) and the minimum and maximum transmissions are 0.749 and 0.813.

The structure was solved in the space group *I*2/*a* (# 15) by ShelXT using dual methods.[6] It was refined by full matrix least squares minimization on $|F|^2$ using version 2019/3 of ShelXL 2019/3.[7] All non-hydrogen atoms were refined anisotropically.

Hydrogen atom positions were calculated geometrically and refined using the riding model.

There is a single formula unit in the asymmetric unit, which is represented by the reported sum formula. In other words: Z is 8 and Z' is 1. The moiety formula is $C_{72}H_{102}K_2N_8O_4$, C_4H_8O.

Table S1. Crystal data and structure refinement for **2**.

**Compound**	**2**
Formula	$C_{76}H_{110}K_2N_8O_5$
$D_{calc.}$/ g cm^{-3}	1.131
m/mm^{-1}	1.506
Formula Weight	1293.91
Color	clear intense yellow
Shape	prism-shaped
Size/mm	0.27×0.20×0.18
T/K	139.99(10)
Crystal System	monoclinic
Space Group	*I*2/*a*
a/Å	46.4290(8)
b/Å	10.45085(12)
c/Å	33.2521(6)
α/°	90
β/°	109.5866(18)
γ/°	90
V/Å^3	15201.0(4)
Z	8
Z'	1
Wavelength/Å	1.54184
Radiation type	Cu K_{α}
Θ_{min}/°	3.984
Θ_{max}/°	72.716
Index range h	-56 ≤ h ≤ 54
Index range k	-12 ≤ k ≤ 10
Index range l	-41 ≤ l ≤ 41
Measured Refl's.	35959
Indep't Refl's	14713
Refl's I≥2σ(I)	12891
R_{int}	0.0199
Parameters	977
Restraints	636
Largest Peak/eÅ^3	0.226
Deepest Hole/eÅ^3	-0.275
GooF	1.029
R_1 (I≥2σ(I) / all)	0.0435 / 0.0494
wR_2 (I≥2σ(I) / all)	0.1169 / 0.1220
CCDC number	2530220

Compound 4

Structure Quality Indicators

**Reflections:**	d min (CuKα) 2Θ=151.5° 0.80	I/σ(I) 77.5	Rint m=7.16 2.29%	Full 135.4° 100	
**Refinement:**	Shift 0.000	Max Peak 0.1	Min Peak -0.2	GooF 1.045	Hooft .001(13)

A clear light yellow prism-shaped crystal with dimensions 0.83 × 0.29 × 0.21 mm was mounted. Data were collected using a SuperNova, Dual, Cu at home/near, AtlasS2 diffractometer operating at T = 140.00(10) K.

Data were measured using ω scans with Cu K_α radiation. The diffraction pattern was indexed and the total number of runs and images was based on the strategy calculation from the program CrysAlis^{Pro} 1.171.42.60a (release 2022). The maximum resolution achieved was Θ = 75.735° (0.80 Å).

The unit cell was refined using CrysAlis^{Pro} on 32226 reflections, 72% of the observed reflections.

Data reduction, scaling and absorption corrections were performed using CrysAlis^{Pro}. The final completeness is 100.00 % out to 75.735° in Θ. A gaussian absorption correction was performed using CrysAlis^{Pro}. The absorption coefficient μ of this crystal is 0.833 mm^{-1} at this wavelength (λ = 1.54184Å) and the minimum and maximum transmissions are 0.497 and 1.000.

The structure was solved in the space group $P6_1$ (# 169) by ShelXT using dual methods. It was refined by full matrix least squares minimization on $|F|^2$ using version 2019/3 of ShelXL. All non-hydrogen atoms were refined anisotropically.

Hydrogen atom positions were calculated geometrically and refined using the riding model.

_smtbx_masks_special_details: A solvent mask was calculated and 56 electrons were found in a volume of 266Å^3 in 1 void per unit cell. This is consistent with the presence of 0.22[C_5H_{12}] per Asymmetric Unit which account for 55 electrons per unit cell.

There is a single formula unit in the asymmetric unit, which is represented by the reported sum formula. In other words: Z is 6 and Z' is 1. The moiety formula is $C_{31}H_{44}N_4Si$, 0.22[C_5H_{12}].

The Flack parameter was refined to 0.001(13). Determination of absolute structure using Bayesian statistics on Bijvoet differences using the Olex2 results in 0.001(13).[8] There are no chiral atoms in this structure. Note: The Flack parameter is used to determine chirality of the crystal studied, the value should be near 0, a value of 1 means that the stereochemistry is

wrong and the model should be inverted. A value of 0.5 means that the crystal consists of a racemic mixture of the two enantiomers.

Table S2. Crystal data and structure refinement for **4**.

**Compound**	**4**
Formula	$C_{32.1}H_{46.64}N_4Si$
$D_{calc.}$/ g cm^{-3}	1.086
μ/mm^{-1}	0.833
Formula Weight	516.66
Color	clear light yellow
Shape	prism-shaped
Size/mm	0.83×0.29×0.21
T/K	140.00(10)
Crystal System	hexagonal
Flack Parameter	0.001(13)
Hooft Parameter	0.001(13)
Space Group	$P6_1$
a/Å	23.55455(12)
b/Å	23.55455(12)
c/Å	9.86204(7)
α/°	90
β/°	90
γ/°	120
V/Å^3	4738.56(6)
Z	6
Z'	1
Wavelength/Å	1.54184
Radiation type	Cu K_α
Θ_{min}/°	3.753
Θ_{max}/°	75.735
Index range h	-29 ≤ h ≤ 29
Index range k	-29 ≤ k ≤ 29
Index range l	-12 ≤ l ≤ 11
Measured Refl's.	44665
Indep't Refl's	6242
Refl's I≥2□(I)	6120
R_{int}	0.0229
Parameters	497
Restraints	604
Largest Peak/eÅ^3	0.132
Deepest Hole/eÅ^3	-0.200
GooF	1.042
R_1 (I≥2σ(I) / all)	0.0300 / 0.0307
wR_2 (I≥2σ(I) / all)	0.0822 / 0.0831
CCDC number	2497210

Compound 7

Structure Quality Indicators

**Reflections:**	d min (CuKα) 2Θ=150.6° 0.80	I/σ(I) 60.6	Rint m=4.98 1.88%	Full 135.4° 98% to 150.6° 99.6
**Refinement:**	Shift 0.001	Max Peak 0.2	Min Peak -0.3	GooF 1.062

A clear intense orange block-shaped crystal with dimensions 0.26 × 0.08 × 0.07 mm was mounted. Data were collected using a XtaLAB Synergy R, DW system, HyPix-Arc 150 diffractometer operating at T = 229.99(10) K.

Data were measured using ω scans with Cu K_α radiation. The diffraction pattern was indexed and the total number of runs and images was based on the strategy calculation from the program CrysAlis^{Pro} 1.171.42.90a (release 2023). The maximum resolution achieved was Θ = 75.311° .

The unit cell was refined using CrysAlis^{Pro} on 21966 reflections, 61% of the observed reflections.

Data reduction, scaling and absorption corrections were performed using CrysAlis^{Pro}. The final completeness is 99.60 % out to 75.311° in Θ. A Gaussian absorption correction was performed using CrysAlis^{Pro} 1.171.42.90a Numerical absorption correction based on Gaussian integration over a multifaceted crystal model. Empirical absorption correction using spherical harmonics as implemented in SCALE3 ABSPACK scaling algorithm. The absorption coefficient μ of this material is 0.916 mm^{-1} at this wavelength (λ = 1.54184Å) and the minimum and maximum transmissions are 0.657 and 1.000.

The structure was solved in the space group $P\bar{1}$ (# 2) by ShelXT 2018/2 using dual methods. It was refined by full matrix least squares minimization on $|F|^2$ using version 2019/3 of ShelXL 2019/3. All non-hydrogen atoms were refined anisotropically.

Hydrogen atom positions were calculated geometrically and refined using the riding model.

There is a single formula unit in the asymmetric unit, which is represented by the reported sum formula. In other words: Z is 2 and Z' is 1. The moiety formula is $C_{40}H_{45}N_4P$.

Table S3. Crystal data and structure refinement for **7**.

**Compound**	**7**
Formula	$C_{40}H_{45}N_4P$
$D_{calc.}$/ g cm^{-3}	1.137
μ/mm^{-1}	0.916
Formula Weight	612.77
Color	clear intense orange
Shape	block-shaped
Size/mm	0.26×0.08×0.07
T/K	229.99(10)
Crystal System	triclinic
Space Group	$P\bar{1}$
a/Å	10.82034(15)
b/Å	11.41209(15)
c/Å	16.03997(18)
α/°	76.8743(10)
β/°	85.0080(10)
γ/°	68.1283(12)
V/Å^3	1790.06(4)
Z	2
Z'	1
Wavelength/Å	1.54184
Radiation type	Cu K_{α}
Θ_{min}/°	2.829
Θ_{max}/°	75.311
Index range h	-13 ≤ h ≤ 13
Index range k	-13 ≤ k ≤ 14
Index range l	-20 ≤ l ≤ 19
Measured Refl's.	36171
Indep't Refl's	7269
Refl's I≥2σ(I)	6314
R_{int}	0.0188
Parameters	435
Restraints	39
Largest Peak/eÅ^3	0.204
Deepest Hole/eÅ^3	-0.267
GooF	1.062
R_1 (I≥2σ(I) / all)	0.0401 / 0.0457
wR_2 (I≥2σ(I) / all)	0.1137 / 0.1172
CCDC number	2533231

Compound 8

Structure Quality Indicators

**Reflections:**	d min (CuKα) 2Θ=149.5° 0.80	I/σ(I) 22.8	Rint m=3.37 3.64%	Full 135.4° 95% to 149.5° 99.1
**Refinement:**	Shift 0.001	Max Peak 3.5	Min Peak -1.7	GooF 1.016

A clear intense yellow prism-shaped crystal with dimensions 0.31 × 0.10 × 0.06 mm was mounted. Data were collected using a XtaLAB Synergy R, DW system, HyPix-Arc 150 diffractometer operating at T = 140.00(10) K.

Data were measured using ω scans with Cu K_α radiation. The diffraction pattern was indexed and the total number of runs and images was based on the strategy calculation from the program CrysAlis^{Pro} 1.171.42.100a (release 2023). The maximum resolution achieved was Θ = 74.738°.

The unit cell was refined using CrysAlis^{Pro} on 13343 reflections, 44% of the observed reflections.

Data reduction, scaling and absorption corrections were performed using CrysAlis^{Pro}. The final completeness is 99.10 % out to 74.738° in Q. A Gaussian absorption correction was performed using CrysAlis^{Pro} 1.171.42.100a Numerical absorption correction based on Gaussian integration over a multifaceted crystal model. Empirical absorption correction using spherical harmonics as implemented in SCALE3 ABSPACK scaling algorithm. The absorption coefficient μ of this material is 4.718 mm^{-1} at this wavelength (λ = 1.54184Å) and the minimum and maximum transmissions are 0.452 and 1.000.

The structure was solved in the space group $P\bar{1}$ (# 2) by ShelXT 2018/2 using dual methods. It was refined by full matrix least squares minimization on $|F|^2$ using version 2019/3 of ShelXL 2019/3. All non-hydrogen atoms were refined anisotropically.

Hydrogen atom positions were calculated geometrically and refined using the riding model.

The residual electron-density peak of 3.63 e Å^{-3}, located **1**.19 Å from Sn1, is attributed to Fourier truncation effects and minor series-termination errors associated with the presence of the heavy Sn atom. The crystal was a very thin platelet and diffracted weakly with Mo radiation; therefore, Cu radiation was used, although this is not ideal for a Sn-containing compound. The less-than-ideal crystal and data quality may also contribute to the observed residual density. No chemically reasonable disorder model or missing atom could account for this peak. The

structure was carefully checked, and all non-hydrogen atoms were refined anisotropically. The residual density is therefore considered acceptable for this structure.

There is a single formula unit in the asymmetric unit, which is represented by the reported sum formula. In other words: Z is 2 and Z' is 1. The moiety formula is $C_{46}H_{51}N_4Sn$, $C_4H_{10}O$.

Table S4. Crystal data and structure refinement for **8**.

**Compound**	**8**
Formula	$C_{50}H_{60}N_4OSn$
$D_{calc.}$/ g cm^{-3}	1.233
μ/mm^{-1}	4.718
Formula Weight	851.71
Color	clear intense yellow
Shape	prism-shaped
Size/mm	0.31×0.10×0.06
T/K	140.00(10)
Crystal System	triclinic
Space Group	$P\bar{1}$
a/Å	12.8834(3)
b/Å	12.9581(3)
c/Å	15.6017(4)
α/°	66.207(2)
β/°	81.991(2)
γ/°	74.455(2)
V/Å^3	2294.62(11)
Z	2
Z'	1
Wavelength/Å	1.54184
Radiation type	Cu K_α
Θ_{min}/°	3.098
Θ_{max}/°	74.738
Index range h	-15 ≤ h ≤ 15
Index range k	-16 ≤ k ≤ 15
Index range l	-18 ≤ l ≤ 11
Measured Refl's.	30116
Indep't Refl's	8924
Refl's I≥2σ(I)	7361
R_{int}	0.0364
Parameters	592
Restraints	177
Largest Peak/eÅ^3	3.481
Deepest Hole/eÅ^3	-1.708
GooF	1.033
R_1 (I≥2σ(I) / all)	0.0704 / 0.0831
wR_2 (I≥2σ(I) / all)	0.1841 / 0.1940
CCDC number	2533232

Compound 9

Structure Quality Indicators

**Reflections:**	d min (CuKα) 2Θ=133.2° 0.84	I/σ(I) 9.4	Rint m=3.45 11.57%	Full 133.2° 99.8
**Refinement:**	Shift 0.000	Max Peak 0.8	Min Peak -0.5	GooF 0.988

A clear light yellow plate-shaped crystal with dimensions 0.22 × 0.06 × 0.02 mm was mounted. Data were collected using a SuperNova, Dual, Cu at home/near, Atlas diffractometer operating at T = 140.00(10) K.

Data were measured using ω scans with Cu K_{α} radiation. The diffraction pattern was indexed and the total number of runs and images was based on the strategy calculation from the program CrysAlis^{Pro} 1.171.42.67a (release 2022). The maximum resolution achieved was Θ = 58.992°.

The unit cell was refined using CrysAlis^{Pro} on 1809 reflections, 9% of the observed reflections.

Data reduction, scaling and absorption corrections were performed using CrysAlis^{Pro}. The final completeness is 100 % out to 58.992° in Θ. A Gaussian absorption correction was performed using CrysAlis^{Pro}. The absorption coefficient μ of this material is 0.491 mm^{-1} at this wavelength (λ = 1.54184Å) and the minimum and maximum transmissions are 0.803 and 1.000.

The structure was solved in the space group *Pbca* (# 61) by ShelXT 2018/2 using dual methods. It was refined by full matrix least squares minimization on $|F|^2$ using version 2019/3 of ShelXL 2019/3. All non-hydrogen atoms were refined anisotropically.

Hydrogen atom positions were calculated geometrically and refined using the riding model.

The crystal used for data collection for bk259 was of limited quality and diffracted weakly; therefore, the data were truncated at 2θ = 118°. The refinement was complicated by solvent disorder, including disorder of the THF molecule containing O1 and a mixed diethyl ether/THF solvent region. Modelling this disorder improved the refinement and provided a more chemically reasonable description of the residual electron density. Despite the limited data quality, the refined model supports the proposed molecular structure.

The value of Z' is 0.5. This means that only half of the formula unit is present in the asymmetric unit, with the other half consisting of symmetry equivalent atoms. The moiety formula is $C_{84}H_{136}B_2Li_2N_8O_4$.

Table S5. Crystal data and structure refinement for **9**.

**Compound**	**9**
Formula	$C_{84}H_{136}B_2Li_2N_8O_4$
$D_{calc.}$/ g cm^{-3}	1.072
μ/mm^{-1}	0.491
Formula Weight	1357.50
Color	clear light yellow
Shape	plate-shaped
Size/mm	0.22×0.06×0.02
T/K	140.00(10)
Crystal System	orthorhombic
Space Group	*Pbca*
a/Å	19.6109(11)
b/Å	17.3927(19)
c/Å	24.660(2)
α/°	90
β/°	90
γ/°	90
V/Å^3	8411.2(12)
Z	4
Z'	0.5
Wavelength/Å	1.54184
Radiation type	Cu K_α
Θ_{min}/°	3.585
Θ_{max}/°	58.992
Index range h	-23 ≤ h ≤ 18
Index range k	-20 ≤ k ≤ 20
Index range l	-29 ≤ l ≤ 25
Measured Refl's.	21068
Indep't Refl's	6042
Refl's I≥2σ(I)	3449
R_{int}	0.1091
Parameters	579
Restraints	313
Largest Peak/eÅ^3	0.356
Deepest Hole/eÅ^3	-0.390
GooF	1.005
R_1 (I≥2σ(I) / all)	0.0741 / 0.1487
wR_2 (I≥2σ(I) / all)	0.1864 / 0.2413
CCDC number	2533230

Compound 10

Structure Quality Indicators

**Reflections:**	d min (CuKα) 2Θ=149.8° 0.80	I/σ(I) 35.0	Rint m=5.13 3.03%	Full 135.4° 98% to 149.8° 100	
**Refinement:**	Shift 0.004	Max Peak 0.4	Min Peak -0.3	GooF 1.010	Hooft 0.032(11)

A clear light yellow irregular-shaped crystal with dimensions 0.16 × 0.13 × 0.11 mm was mounted. Data were collected using a XtaLAB Synergy R, DW system, HyPix-Arc 150 diffractometer operating at T = 140.00(10) K.

Data were measured using ω scans with Cu K_{α} radiation. The diffraction pattern was indexed and the total number of runs and images was based on the strategy calculation from the program CrysAlis^{Pro} system (CCD 43.143a 64-bit (release 25-10-2024)). The maximum resolution achieved was Θ = 74.883° .

The unit cell was refined using CrysAlis^{Pro} on 41227 reflections, 35% of the observed reflections.

Data reduction, scaling and absorption corrections were performed using CrysAlis^{Pro}. The final completeness is 100.00 % out to 74.883° in Θ. A Gaussian absorption correction was performed using CrysAlis^{Pro} 1.171.43.141a Numerical absorption correction based on Gaussian integration over a multifaceted crystal model. Empirical absorption correction using spherical harmonics as implemented in SCALE3 ABSPACK scaling algorithm. The absorption coefficient μ of this material is 0.624 mm^{-1} at this wavelength (λ = 1.54184Å) and the minimum and maximum transmissions are 0.787 and 1.000.

The structure was solved in the space group $Pna2_1$ (# 33) by ShelXT 2018/2 using dual methods. It was refined by full matrix least squares minimization on $|F|^2$ using version 2019/3 of ShelXL 2019/3. All non-hydrogen atoms were refined anisotropically.

Hydrogen atom positions were calculated geometrically and refined using the riding model.

A solvent mask was calculated and 708 electrons were found in a volume of 3878Å^3 in 1 void per unit cell. This is consistent with the presence of 2.125[C_5H_{12}] per Asymmetric Unit which account for 714 electrons per unit cell.

The value of Z' is 2. This means that there are two independent molecules in the asymmetric unit. The moiety formula is $C_{68}H_{80}N_8Si$, 2.125[C_5H_{12}].

The Flack parameter was refined to 0.032(11). Determination of absolute structure using

Bayesian statistics on Bijvoet differences using the Olex2 results in 0.065(8). There are no chiral atoms in this structure. Note: The Flack parameter is used to determine chirality of the crystal studied, the value should be near 0, a value of 1 means that the stereochemistry is wrong and the model should be inverted. A value of 0.5 means that the crystal consists of a racemic mixture of the two enantiomers.

Table S6. Crystal data and structure refinement for **10**.

**Compound**	**10**
Formula	$C_{78.62}H_{105.5}N_8Si$
$D_{calc.}$/ g cm^{-3}	1.073
m/mm^{-1}	0.624
Formula Weight	1190.73
Color	clear light yellow
Shape	irregular-shaped
Size/mm	0.16×0.13×0.11
T/K	140.00(10)
Crystal System	orthorhombic
Flack Parameter	0.032(11)
Hooft Parameter	0.065(8)
Space Group	$Pna2_1$
a/Å	27.7844(2)
b/Å	14.65749(13)
c/Å	36.1886(2)
α/°	90
β/°	90
γ/°	90
V/Å^3	14737.75(19)
Z	8
Z'	2
Wavelength/Å	1.54184
Radiation type	Cu K_α
Θ_{min}/°	2.442
Θ_{max}/°	74.883
Index range h	-34 ≤ h ≤ 34
Index range k	-18 ≤ k ≤ 17
Index range l	-30 ≤ l ≤ 44
Measured Refl's.	119265
Indep't Refl's	24394
Refl's I≥2σ(I)	19971
R_{int}	0.0303
Parameters	1460
Restraints	967
Largest Peak/eÅ^3	0.384
Deepest Hole/eÅ^3	-0.298
GooF	1.017
R_1 (I≥2σ(I) / all)	0.0448 / 0.0562
wR_2 (I≥2σ(I) / all)	0.1166 / 0.1234
CCDC number	2533233

Compound 11

Structure Quality Indicators

**Reflections:**	d min (CuKα) 2Θ=130.2° 0.85	I/σ(I) 14.9	Rint m=4.67 9.64%	Full 130.2° 99.9
**Refinement:**	Shift 0.001	Max Peak 0.5	Min Peak -0.4	GooF 0.996

A clear intense orange prism-shaped crystal with dimensions 0.25 × 0.18 × 0.11 mm was mounted. Data were collected using a XtaLAB Synergy R, DW system, HyPix-Arc 150 diffractometer operating at T = 100.00(10) K.

Data were measured using ω scans with Cu K_α radiation. The diffraction pattern was indexed and the total number of runs and images was based on the strategy calculation from the program CrysAlis^{Pro} system (CCD 44.125a 64-bit (release 17-10-2025)). The maximum resolution achieved was Θ = 65.088° .

The unit cell was refined using CrysAlis^{Pro} on 13981 reflections, 21% of the observed reflections.

Data reduction, scaling and absorption corrections were performed using CrysAlis^{Pro}. The final completeness is 99.90 % out to 65.088° in Θ. A Gaussian absorption correction was performed using CrysAlis^{Pro} 1.171.44.120a Numerical absorption correction based on Gaussian integration over a multifaceted crystal model. Empirical absorption correction using spherical harmonics as implemented in SCALE3 ABSPACK scaling algorithm. The absorption coefficient μ of this material is 1.386 mm^{-1} at this wavelength (λ = 1.54184Å) and the minimum and maximum transmissions are 0.460 and 1.000.

The structure was solved in the space group $P\bar{1}$ (# 2) by ShelXT 2018/2 using dual methods. It was refined by full matrix least squares minimization on $|F|^2$ using version 2019/3 of ShelXL 2019/3. All non-hydrogen atoms were refined anisotropically.

Hydrogen atom positions were calculated geometrically and refined using the riding model.

A solvent mask was calculated and 131 electrons were found in a volume of 950Å^3 in 3 voids per unit cell. This is consistent with the presence of 1.5 [C_4H_8O] per Asymmetric Unit which account for 120 electrons per unit cell.

There is a single formula unit in the asymmetric unit, which is represented by the reported sum formula. In other words: Z is 2 and Z' is 1. The moiety formula is $C_{82}H_{90}K_2N_8O_2$, 1.5 [C_4H_8O].

Table S7. Crystal data and structure refinement for **11**.

**Compound**	**11**
Formula	$C_{88}H_{102}K_2N_8O_{3.5}$
$D_{calc.}$/ g cm^{-3}	1.107
m/mm^{-1}	1.386
Formula Weight	1405.97
Color	clear intense orange
Shape	prism-shaped
Size/mm	0.25×0.18×0.11
T/K	100.00(10)
Crystal System	triclinic
Space Group	$P\bar{1}$
a/Å	14.1522(4)
b/Å	16.5371(4)
c/Å	19.8875(5)
α/°	110.877(2)
β/°	96.539(2)
γ/°	99.312(2)
V/Å^3	4216.6(2)
Z	2
Z'	1
Wavelength/Å	1.54184
Radiation type	Cu K_α
Θ_{min}/°	2.420
Θ_{max}/°	65.088
Index range h	-16 ≤ h ≤ 16
Index range k	-19 ≤ k ≤ 19
Index range l	-21 ≤ l ≤ 23
Measured Refl's.	67210
Indep't Refl's	14382
Refl's I≥2σ(I)	9862
R_{int}	0.0964
Parameters	884
Restraints	42
Largest Peak/eÅ^3	0.460
Deepest Hole/eÅ^3	-0.395
GooF	1.008
R_1 (I≥2σ(I) / all)	0.0682 / 0.0915
wR_2 (I≥2σ(I) / all)	0.1924 / 0.2085
CCDC number	2533234

Compound 12

Structure Quality Indicators

**Reflections:**	d min (CuKα) 2Θ=144.8° 0.81	I/σ(I) 19.9	Rint m=2.06 4.24%	Full 135.4° 96% to 144.8° 98.3
**Refinement:**	Shift 0.000	Max Peak 0.5	Min Peak -0.4	GooF 1.051

A clear pale-yellow irregular-shaped crystal with dimensions 0.21 × 0.19 × 0.13 mm was mounted. Data were collected using a SuperNova, Dual, Cu at home/near, AtlasS2 diffractometer operating at T = 140.01(10) K.

Data were measured using ω scans with Cu K_a radiation. The diffraction pattern was indexed and the total number of runs and images was based on the strategy calculation from the program CrysAlis^{Pro} system (CCD 44.122a 64-bit (release 14-09-2025)). The maximum resolution achieved was θ = 72.376° (0.81 Å).

The unit cell was refined using CrysAlis^{Pro} on 8074 reflections, 52% of the observed reflections.

Data reduction, scaling and absorption corrections were performed using CrysAlis^{Pro}. The final completeness is 98.30 % out to 72.376° in Θ. A gaussian absorption correction was performed using CrysAlis^{Pro} 1.171.44.125a. The numerical absorption correction was based on gaussian integration over a multifaceted crystal model. The empirical absorption correction was done using spherical harmonics, implemented in SCALE3 ABSPACK scaling algorithm. The absorption coefficient μ of this crystal is 0.801 mm^{-1} at this wavelength (λ = 1.54184Å) and the minimum and maximum transmissions are 0.703 and 1.000.

The structure was solved in the space group P-1 (# 2) by ShelXT using dual methods. It was refined by full matrix least squares minimization on $|F|^2$ using version 2019/3 of ShelXL. All non-hydrogen atoms were refined anisotropically.

Hydrogen atom positions were calculated geometrically and refined using the riding model.

There is a single formula unit in the asymmetric unit, which is represented by the reported sum formula. In other words: Z is 2 and Z' is 1. The moiety formula is $C_{44}H_{54}N_4OSi$.

Table S8. Crystal data and structure refinement for **12**.

**Compound**	**12**
Formula	$C_{44}H_{54}N_4OSi$
$D_{calc.}$/ g cm^{-3}	1.141
μ/mm^{-1}	0.801
Formula Weight	683.00
Color	clear pale yellow
Shape	irregular-shaped
Size/mm	0.21×0.19×0.13
T/K	140.01(10)
Crystal System	triclinic
Space Group	*P*-1
a/Å	11.0088(4)
b/Å	11.0488(4)
c/Å	18.9987(5)
α/°	91.140(3)
β/°	103.103(3)
γ/°	116.898(4)
V/Å^3	1987.18(13)
Z	2
Z'	1
Wavelength/Å	1.54184
Radiation type	Cu K_a
Θ_{min}/°	4.533
Θ_{max}/°	72.376
Index range h	-13 ≤ h ≤ 13
Index range k	-13 ≤ k ≤ 13
Index range l	-12 ≤ l ≤ 22
Measured Refl's.	15512
Indep't Refl's	7530
Refl's I≥2*σ*(I)	6401
R_{int}	0.0424
Parameters	462
Restraints	0
Largest Peak/eÅ^3	0.543
Deepest Hole/eÅ^3	-0.427
GooF	1.051
R_1 (I≥2*σ*(I) / all)	0.0610 / 0.0700
wR_2 (I≥2*σ*(I) / all)	0.1579 / 0.1665
CCDC number	2497211

6. References

1 P. Varava, Z. Dong, R. Scopelliti, F. Fadaei-Tirani and K. Severin, *Nat. Chem.,* 2021, **13**, 1055–1060.

2 B. Kooij, Z. Dong, F. Fadaei-Tirani, R. Scopelliti and K. Severin, *Angew. Chem., Int. Ed.*, 2023, **62**, e202308625.

3 S. Kundu, S. Sinhababu, M. M. Siddiqui, A. V. Luebben, B. Dittrich, T. Yang, G. Frenking and H. W. Roesky, *J. Am. Chem. Soc.*, 2018, **140**, 9409–9412.

4 CrysAlis^{Pro} Software System, Rigaku Oxford Diffraction, 2022 to 2026.

5 R. C. Clark and J. S. Reid, 1995, *Acta Cryst.*, **A51**, 887-897.

6 G. M. Sheldrick, *Acta Cryst.*, 2015, **A71**, 3-8.

7 G. M. Sheldrick, *Acta Cryst.*, 2015, **C71**, 3-8.

8 O. V. Dolomanov, L. J. Bourhis, R. J. Gildea, J. A. K. Howard and H. Puschmann, *J. Appl. Cryst.*, 2009, **42**, 339-341.
